# Design and synthesis of antiproliferative 2-oxoindolin-3-ylidenes incorporating urea function with potential VEGFR-2 inhibitory properties

**DOI:** 10.1038/s41598-024-82005-6

**Published:** 2025-01-03

**Authors:** Dalia R. Aboshouk, M. Adel Youssef, Siva S. Panda, Benson M. Kariuki, Mohamed S. Bekheit, Ahmed R. Hamed, Walid Fayad, Ahmed A. F. Soliman, Adel S. Girgis

**Affiliations:** 1 Department of Pesticide Chemistry, National Research Centre, Dokki, 12622 Giza Egypt; 2 Department of Chemistry, Faculty of Science, Helwan University, Helwan, Egypt; 3 Department of Chemistry and Biochemistry & Department of Biochemistry and Molecular Biology, Augusta University, Augusta, GA 30912 USA; 4 School of Chemistry, Cardiff University, Main Building, Park Place, Cardiff, CF10 3AT UK; 5 Chemistry of Medicinal Plants Department, National Research Centre, Dokki, 12622 Giza Egypt; 6 Drug Bioassay-Cell Culture Laboratory, Pharmacognosy Department, National Research Centre, Dokki, 12622 Giza Egypt

**Keywords:** Cancer, 2-Oxoindolin-3-ylidenes, VEGFR-2, Molecular modeling, Cancer, Breast cancer

## Abstract

Targeted therapy is preferable over other therapeutics due to its limitation of drawbacks and better pharmaceutical outcomes. VEGF and its receptors have been observed to be hyper-activated in many cancer types and are considered promising targets for assigning anticancer agents. The current study is directed towards synthesis of novel antiproliferative 2-oxoindolin-3-ylidenes incorporating urea function with VEGFR-2 properties. The targeted agents were obtained through a two-step reaction. Addition of the appropriate 1-(acetylphenyl)-3-phenylurea **9a,b** to the corresponding isatin **10a–f** in ethanol containing a quantitative amount of Et_2_NH followed by acidic dehydration (AcOH/HCl) afforded the targeted agents **12a–j**. Promising antiproliferation properties (MTT assay) were observed for most of the synthesized agents against HCT116 (colon), MCF7 (breast) and PaCa2 (pancreatic) cancer cell lines relative to sunitinib. VEGFR-2 inhibitory properties are consistent with the antiproliferation properties exhibited against the tested cell lines. Compound **12b** (R = 4-NHCONHPh, R′ = H; % inhibition = 87.2) is the most promising/potent anti-VEGFR-2 agent synthesized with activity close to that of sunitinib (% inhibition = 89.4) at 10 μM. Molecular docking studies (PDB: 3WZE and 3AGD) support the antiproliferation effects against cancer cell lines tested with VEGFR-2 inhibitory properties. The results are consistent with collaboration of the pharmacophores considered (2-oxoindolyl heterocycle and urea) in improving the bio-properties.

## Introduction

One of the most dangerous diseases globally impacting human-kind is cancer^1^. Diverse techniques continue to be developed for diagnosis and treatment of different cancer types but control the disease remains elusive^2^. Targeted therapy is a clinically preferable approach over conventional/traditional treatment due to lower cytotoxicity (causing damage to normal cells and impacting vital body organs) and better efficacy. Many small and large molecule targeted therapeutical hits/leads have been the subject of different research studies, with small molecules being preferable due to the improved pharmacokinetics^3^.

The current work deals with construction of novel VEGFR-2 (vascular endothelial growth factor receptor) inhibitors adopting the molecular hybridization approach. Molecular hybridization is an approach involving structure modification in drug discovery and development. The reasoning is that combination of two or more bio-active pharmacophoric units/moieties may afford new potentially bio-active hybrids with elevated potency relative to the parents. This concept can be achieved through direct connection of two or more pharmacophoric units or through a linker/spacer^4–6^.

VEGF is an important class of tyrosine kinases. Tyrosine kinases are reported to be overexpressed in cancer cells and are linked to cancer proliferation and metastasis. This explains the efficiency of chemotherapeutical pathway of cancer proliferation associated with their inhibition^7,8^. Tyrosine kinases fall into two categories, receptors and non-receptors. The receptors are either trans-membrane, extracellular or intracellular whereas, the non-receptors are intracellular^9^. VEGF are essential growth factor protein kinases for angiogenesis, which is a vital process for the generation of new blood capillaries from the present vessels of the vascular system. Angiogenesis is a normal and important process for many functions, including wound healing and functional repair of many pathological disorders in addition to embryonic development. Cancer proliferation and development utilize angiogenesis for vital nutrients, oxygen supply and removal of waste^10–12^. Several VEGF have been identified (VEGF-A, B, C and D), capable of binding with different tyrosine kinase receptors (VEGFR-1, -2, and -3). VEGF and its receptors have been observed to be hyper-activated in many cancer types making them promising targets for anticancer agents. VEGFR-2 is reported to be an anti-angiogenic factor against many solid tumors (breast, ovary, colon, lung, renal, etc.)^10–12^.

**Fig. 1:**
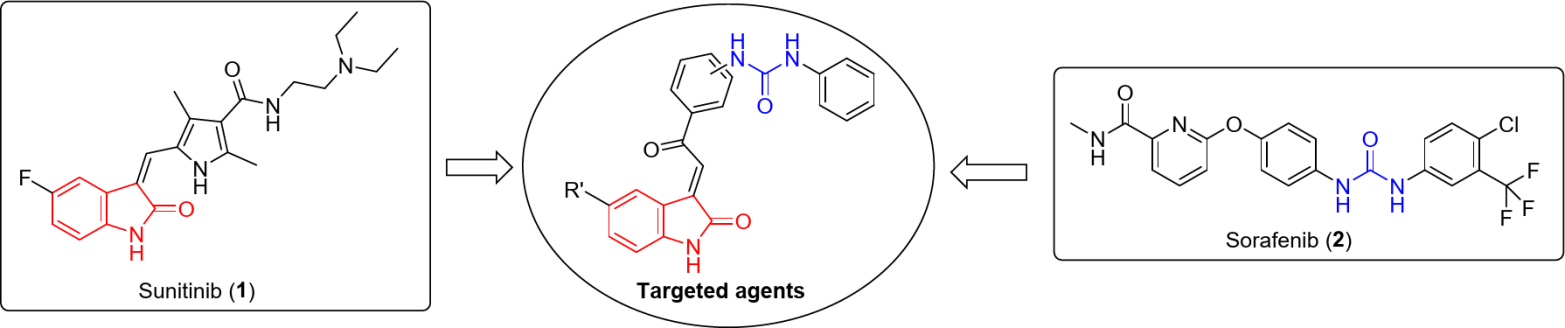
Design of the targeted agents via conjugation of pharmacophores derived from VEGF inhibitor FDA approved Sunitinib **1** (indoleyl-containing) and Sorafenib **2** (urea-containing) drugs.

Many indolyl heterocycles have been assessed as anticancer active agents^13–22^ and some have been clinically approved against different types of cancers. Sunitinib **1** (Fig. 1) is a multi-targeted tyrosine kinase inhibitor (VEGFR-1,-2, -3; PDGFR-α, -β “platelet derived growth-factor receptor”; c-kit “stem cell factor receptor”)^23–26^. It has been awarded FDA clinical approval against advanced renal and imatinib-resistant gastrointestinal (2006) and pancreatic (2011) cancers, as well as adjuvant treatment of adults at high risk of renal cancer (2017)^27,28^. Many urea-containing compounds have been reported as VEGFR-2 inhibitors^29–34^ and some have been approved against different cancer types. Sorafenib (Nexavar) **2** is a multi-targeted kinase inhibitor, FDA approved against advanced renal (2005), hepatocellular (2007) and thyroid (2013) cancers^35,36^ (Fig. 1).

Additionally, many indolyl-containing anticancer drugs are well known of which, Nintedanib **3** that is a multi-targeted tyrosine kinase inhibitor (VEGFR-1, -2, -3; PDGFR-α, -β and FGFR-1, -2, -3, -4 “fibroblast growth-factor receptor”)^37–41^. FDA approval for Nintedanib was received in 2014 against lung fibrosis and in 2019. It was awarded approval for non-small cell lung cancer “NSCLC” (accompanied with Docetaxel)^37,39–43^. Anlotinib **4** (against NSCLC and metastatic colon cancers) and Surufatinib **5** (against extrapancreatic neuroendocrine tumor) are multi-targeted tyrosine kinase inhibitors approved in China^44–47^.

**Fig. 2:**
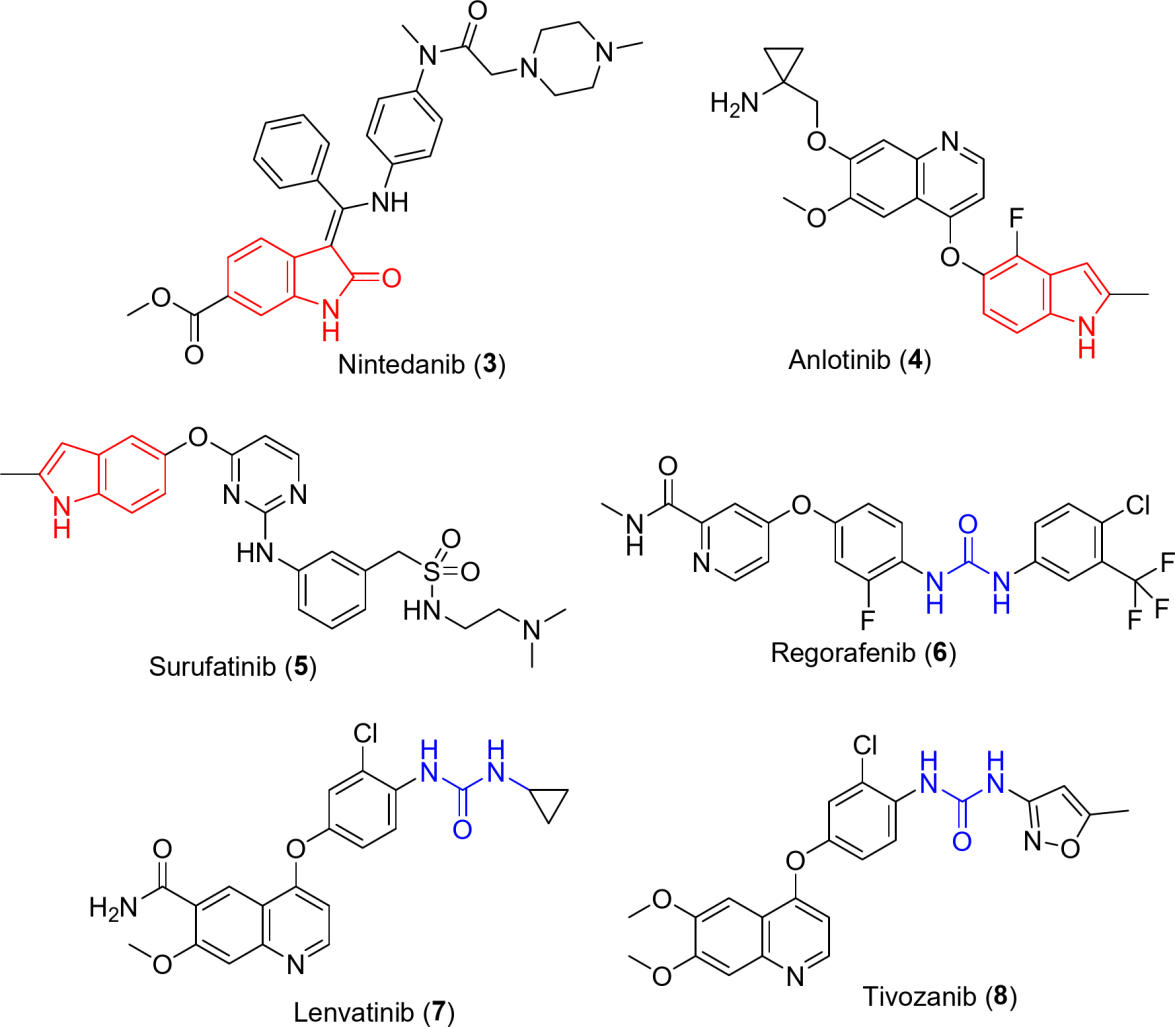
Antitumor drugs containing-indolyl or urea pharmacophores.

Moreover, many urea-containing drugs are well known of which, Regorafenib (Stivarga) **6**, that is FDA approved drug against metastatic colon (2012), advanced gastrointestinal (2013) and liver (2017) cancers^48,49^. Lenvatinib (Lenvima) **7** is multi-targeted tyrosine kinase inhibitor (VEGR-1, -2, -3; FGFR-1, -2, -3, -4 and PDGFR-α) FDA approved against thyroid (2015), advanced renal (2016), liver (2018) and endometrial (in combination with Keytruda, 2019, 2021) cancers^50,51^. Tivozanib (Fotivda) **8** is an anti-angiogenic VEGFR inhibitor, FDA approved (2021) for adult patient with relapsed or advanced renal cancer^52,53^ (Fig. 2).

The current study investigates the design and synthesis of novel agents with potential VEGFR-2 inhibitory properties via conjugation of the 2-oxoindolyl heterocyclic moiety with urea function. This is inspired by the clinically approved indole- and urea-containing anticancer drugs (Figs. 1 and 2). Molecular conjugation is an approach extensively applicable in medicinal chemistry for designing/optimizing effective hits/leads. Conjugation of one or more pharmacophores can assist in development of potent bio-active agents^54^. Many bio-active agents for diverse medicinal chemical purposes (for example, anticancer^54,55^, antimicrobial^56^, cardiovascular disorders^57,58^ and anti-inflammatory^56,59^) adopting molecular conjugation approach have been reported. Antiproliferation properties of the synthesized agents against a group of cancer cell lines (colon, breast and pancreatic) have been investigated. The VEGFR-2 inhibitory properties of the agents have also been determined and molecular modeling (computational studies) used for understanding and explaining the exhibited bio-properties.

Colorectal cancer (including colon and rectum) accounts for about 10% of all malignant cancer types (the third most common cancer) and is the second most deadly globally^60^. Colon cancers are of two main types, intraluminal and perforated^61^. Many lifestyle habits such of which; smoking, alcohol drinking and unhealthy food are connected with colon cancer^62^. It is also notable that the incidence of colon cancer is higher in elder people (aged 50 years and above) and it is also correlated with other factors such as family history of colon or bowel disease^63^. At the initial stage of the disease, the symptoms are mild or unnoticeable, explaining why medical advice is sought by many patients at an advanced stage/phase^63^. Surgery, chemo- and radiotherapy are the most well-known options for colon cancer treatment. Many chemotherapeutical drugs have been clinically approved, but due to their limited efficacy and severe side effects, alternative therapies continue to be in demand^64^.

Breast cancer is the second highest cause of mortality in women. It can be categorized into either the type with receptors (estrogen, progesterone and human epidermal growth factor) or the triple-negative type that lacks receptors^65^. The latter is an aggressive type, accounting for 15–20% of all breast cancers and is a greater treatment challenge than many other types, with high mortality rates^66,67^. Many approaches are taken towards treatment of breast cancer, including surgery, radiotherapy, chemotherapy, hormone therapy and immunotherapy^66,68^. Metastasis in breast cancer is unfortunately a serious challenge and may lead to transfer of the disease to many vital organs including lung, bone and lymph nodes^68^. Although various tools for diagnosis and many therapeutics have been developed for breast cancer, novel agents with high potencies are still needed^67,68^.

Pancreatic cancer is ranked 12th in terms of occurrence worldwide, with poor prognosis and a low 5-year survival rate (≈ 10.8%)^69,70^. It is predicted to become the second highest cause of death among all cancer types within the next decade unless improved therapeutics and advanced techniques/tools can treat and detect the disease before the advanced or metastatic stages^71^. The low survival rates of pancreatic cancer can be attributed to the lack of a clinical marker that can detect the malignancy in its early stage beside the asymptomatic features of this type of cancer^72^. Four stages of pancreatic cancer have been identified; resectable, borderline resectable, locally advanced and metastatic/advanced stage^71^. Smoking and family disease history are factors associated to this disease. Other contributors to increased risk include alcohol drinking, red meat as well as foods containing saturated fatty acids and fructose^69^. Surgery, chemo- and radiotherapy are the main options for tackling the disease^73^. New effective/selective therapeutic agents are also urgently in demand^74^.

## Results and discussion

### Chemical synthesis

**Fig. 3:**
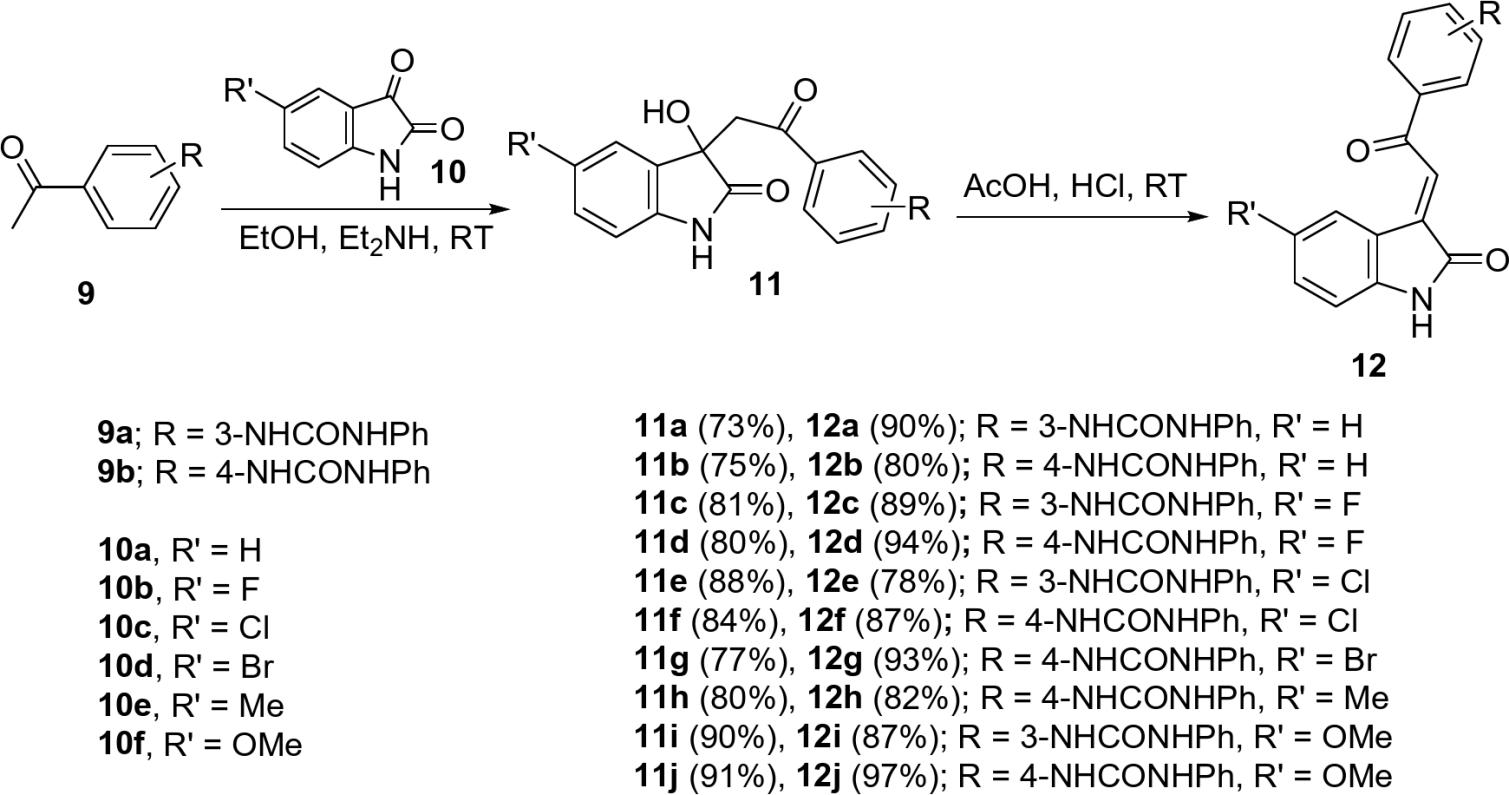
Synthetic route towards the targeted **12a–j**.

The targeted agents **12a–j** were synthesized in a two-step reaction as depicted in Fig. 3. In the first step, addition of the appropriate 1-(acetylphenyl)-3-phenylurea **9a,b**^75,76^ to the corresponding isatin **10a–f** was conducted in ethanol at room temperature in presence of a quantitative amount of diethylamine to afford the 1-[2-(3-hydroxy-2-oxoindolin-3-yl)acetyl]phenyl-3-phenylureas **11a–j** in good to excellent yields (73–91%) and reasonable purity. So, the next step of the reaction sequence was conducted without further purification. The diastereotopic methylene protons of **11a–f** appear in ^1^H-NMR spectra at δ_H_ = 3.51–3.63, 3.97–4.13 (*J* = 17.2–17.9 Hz). Additionally, the appearance of the methylene (δ_C_ = 45.2–45.7) and indolyl C-3 (δ_C_ = 72.9–73.4) signals also evidence the structure.

Acidic dehydration of **11a–f** in glacial acetic acid containing hydrochloric acid (35%) afforded the corresponding 1-[2-(2-oxoindolin-3-ylidene)acetyl]phenyl-3-phenylureas **12a–f** in high yield (78–97%). The stereoselectivity of the reaction was established due to the formation of the *E*-configuration as a sole product. The appearance of the olefinic proton at δ_H_ = 7.70–7.96 evidenced the stereochemical structure^21^ (Supplementary Figs. S1–S60). Single crystal X-ray **12a** provided an addition support for the structure (Fig. 4).

**Fig. 4:**
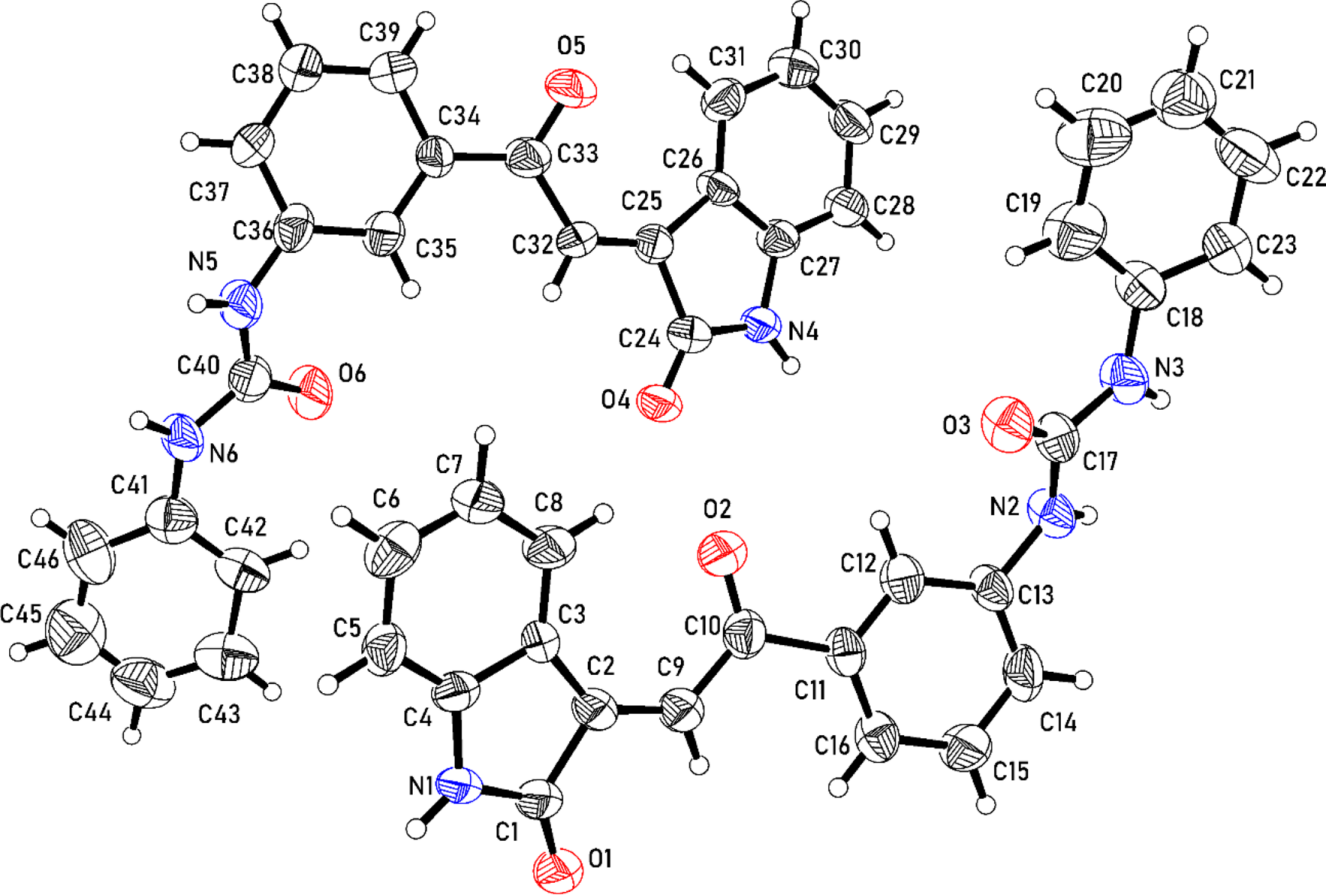
Ortep representation (50% probability ellipsoids) of the unique molecules in the crystal structure of compound **12a**.

### Single crystal X-ray structure of 12a

The crystal structure is orthorhombic, space group Pna2_1_ (Supplementary Table S1), and contains two independent molecules in the asymmetric unit (Fig. 4). Each molecule is composed of four segments, namely indolone [(indo_1_, C1–C8, N1, O1) and (indo_2_, C24–C31, N4, O4)], phenylethanone [(pheneth_1_, C9–C16, O2) and (phenth_2_, C32–C39, O5)], urea [(urea_1_, C17, N2, N3, O3) and (urea_2_, C40, N5, N6, O6)], phenyl [(phen_1_, C18–C23) and (phen_2_, C41–C46)] groups. The conformations of the two independent molecules are similar, as illustrated comparison of the relatively low twist angles indo_1_/pheneth_1_ of 18.47 (28)°, pheneth_1_/urea_1_ of 35.82 (29)°, and urea_1_/phen_1_ of 29.46 (46)° for the first type of molecule with angles indo_2_/pheneth_2_ of 20.66 (28)°, pheneth_2_/urea_2_ of 36.52 (32)° and urea_2_/phen_2_ of 30.96 (48)° for the second type of molecule.

In the crystal, hydrogen bonding of type N–H…O occurs between neighboring molecules of the same type. In the molecule of the first type, interaction between the indolone groups has a N1-H1…O1 bond angle of 142.1° and a N1…O1 distance of 2.989(12) Å and the corresponding N4-H4…O4 bond angle is 139.4° and the N4…O4 distance is 2.984(11) Å for the second type of molecule.

Each oxygen atom of the urea groups is an acceptor of two hydrogen bonds from the urea group of an adjacent molecule of the same type. Angles N2-H2…O3 of 158.3° and N3-H3…O3 of 156.0° are observed for the first type of molecule. The N2…O3 and N3…O3 distances are 2.936(11) Å and 2.976(11) Å respectively. For the second type of molecule, angles N5-H5A…O6 and N6-H6A…O6 are 157.9° and 155.9° respectively. The associated distances N5…O6 and N6…O6 distances are 2.876(11) Å and 2.996(11) Å respectively.

### Antiproliferation properties

Antiproliferation properties of the targeted 2-oxoindolin-3-ylidenes incorporating the urea function **12a–j** were determined by the standard MTT [3-(4,5-dimethylthiazol-2-yl)-2,5-diphenyl-tetrazolium bromide] technique^77,78^ against HCT116 (colon), MCF7 (breast) and PaCa2 (pancreatic) carcinoma cell lines (cell lines were kindly gifted by Prof. Stig Linder, Karolinska Institute, Stockholm, Sweden, originally purchased from ATCC) and compared with the standard reference (sunitinib). Antiproliferation properties against normal RPE1 (human immortalized retinal pigment epithelial) cell line were also considered for safety/selectivity index (SI) determination of each respective agent synthesized (Table 1, Supplementary Figs. S61–S64).

**Table 1:** Antiproliferation properties of **12a–j** and sunitinib.

Compd	IC_50_, μM ± SD (SI)			
	HCT116	MCF7	PaCa2	RPE1
**12a**	13.350 ± 1.352 (1.022)	3.132 ± 0.395 (4.355)	5.235 ± 0.502 (2.606)	13.640 ± 0.659
**12b**	6.545 ± 0.452 (1.277)	2.082 ± 0.126 (4.014)	4.092 ± 0.172 (2.043)	8.358 ± 0.374
**12c**	14.370 ± 1.472 (1.483)	5.808 ± 0.629 (3.669)	6.802 ± 0.514 (3.133)	21.310 ± 0.026
**12d**	5.779 ± 0.593 (1.898)	2.903 ± 0.300 (3.779)	4.955 ± 0.201 (2.214)	10.970 ± 0.429
**12e**	11.890 ± 2.531 (1.229)	3.908 ± 0.780 (3.738)	7.001 ± 1.114 (2.087)	14.610 ± 0.316
**12f**	6.577 ± 0.923 (1.630)	3.410 ± 0.761 (3.144)	5.811 ± 0.630 (1.845)	10.720 ± 0.766
**12g**	6.295 ± 0.912 (2.173)	4.411 ± 0.544 (3.101)	5.319 ± 0.228 (2.572)	13.680 ± 0.850
**12h**	5.581 ± 0.636 (1.523)	2.070 ± 0.081 (4.107)	3.995 ± 0.369 (2.128)	8.501 ± 0.466
**12i**	27.170 ± 2.950 (1.111)	8.958 ± 0.767 (3.370)	7.639 ± 0.080 (3.952)	30.190 ± 0.948
**12j**	6.145 ± 0.539 (1.400)	1.690 ± 0.067 (5.090)	4.488 ± 0.785 (1.917)	8.602 ± 0.334
**Sunitinib**	9.573 ± 0.949	11.770 ± 0.937	13.34 ± 0.091	–-

### HCT116 cell line

It noteworthy that some of the synthesized agents have antiproliferation properties higher than that of sunitinib (standard reference/drug). Compound **12h** (R = 4-NHCONHPh, R′ = Me) is the most potent agent synthesized with an IC_50_ value of 5.581 μM (the value for sunitinib, the standard drug, is 9.573 μM). Compound **12d** (R = 4-NHCONHPh, R′ = F; IC_50_ = 5.779 μM) also exhibits a potency close to that of **12h**. Compounds **12b** (R = 4-NHCONHPh, R′ = H), **12f** (R = 4-NHCONHPh, R′ = Cl), **12g** (R = 4-NHCONHPh, R′ = Br) and **12j** (R = 4-NHCONHPh, R′ = OMe) also show high potency (IC_50_ = 6.145–6.577 μM). Based on the exhibited antiproliferation properties, structure–activity relationships (SARs) can be identified. Compounds containing the 4-phenylurea group display higher anti-HCT116 properties than the 3-substituted analogs, as illustrated by the observations for pairs **12b**/**12a** (IC_50_ = 6.545/13.350 μM), **12d**/**12c** (IC_50_ = 5.779/14.370 μM), **12f**/**12e** (IC_50_ = 6.577/11.890 μM) and **12j**/**12i** (IC_50_ = 6.145/27.170 μM), respectively. The methylindolyl-containing compound has higher anti-HCT116 properties than the methoxy-substituted analog as seen for **12h**/**12j** (IC_50_ = 5.581/6.145 μM, respectively). Also, enhanced antiproliferation properties were noticed by the methylindolyl-containing compound relative to the halogenated substituted analogs as seen for **12d**/**12f**/**12g**/**12h** (IC_50_ = 5.779/6.577/6.295/5.581 μM, respectively).

### MCF7 cell line

All the synthesized agents exhibited better anti-MCF7 proliferation properties (IC_50_ = 1.690–8.958 μM) than that of sunitinib (IC_50_ = 11.770 μM). Compound **12j** (R = 4-NHCONHPh, R′ = OMe) is the most potent analog discovered against MCF7 (IC_50_ = 1.690 μM). Compounds **12b** (R = 4-NHCONHPh, R′ = H), **12d** (R = 4-NHCONHPh, R′ = F) and **12h** (R = 4-NHCONHPh, R′ = Me) also exhibit comparable efficacies (IC_50_ = 2.070–2.903 μM).

SARs associated with the anti-MCF7 properties include the observation that compounds with the *p*-phenylurea residue are more potent than the *m*-substituted analogs. This is supported by the observations for pairs **12b**/**12a** (IC_50_ = 2.082/3.132 μM), **12d**/**12c** (IC_50_ = 2.903/5.808 μM), **12f**/**12e** (IC_50_ = 3.410/3.908 μM) and **12j**/**12i** (IC_50_ = 1.690/8.958 μM), respectively. Substitution with electron-withdrawing elements (fluoro, chloro or bromo) reduces the anti-MCF7 properties as illustrated by compounds **12a**/**12c**/**12e** (IC_50_ = 3.132/5.808/3.908 μM) and **12b**/**12d**/**12f**/**12g** (IC_50_ = 2.082/2.903/3.410/4.411 μM), respectively. It is also discernible that, the methoxyindolyl-containing compound **12j** exhibits enhanced anti-MCF7 properties relative to the methylindolyl analog **12h** (IC_50_ = 1.690, 2.070 μM, respectively). Indolyl substituted by electron donating group (methyl/methoxy) can optimize better antiproliferative active agents than that substituted with halogen (fluorine/chlorine/bromine, electron-withdrawing function) as seen in compounds **12d**/**12f**/**12g**/**12h**/**12j** (IC_50_ = 2.903/3.410/4.411/2.070/1.690 μM, respectively).

### PaCa2 cell line

All the targeted agents synthesized **12a–j** display higher potency (IC_50_ = 3.995–7.639 μM) than sunitinib (IC_50_ = 13.34 μM), noting that it is an approved drug against pancreatic cancer^27,28^. Compound **12h** (R = 4-NHCONHPh, R′ = Me) is the most effective agent synthesized (IC_50_ = 3.995 μM), i.e., it is about 3.34 fold more potent than sunitinib. Compounds **12b** (R = 4-NHCONHPh, R′ = H; IC_50_ = 4.092 μM), **12d** (R = 4-NHCONHPh, R′ = F; IC_50_ = 4.955 μM) and **12j** (R = 4-NHCONHPh, R′ = OMe; IC_50_ = 4.488 μM) also show promising anti-PaCa2 properties.

SARs derived from the results indicate that the synthesized agents containing *p*-phenylurea have greater anti-PaCa2 properties than the *m*-substituted analogs. This appears to be a general rule applicable to all the tested analogs. The anti-PaCa2 properties of pairs **12b**/**12a** (IC_50_ = 4.092/5.235 μM), **12d**/**12c** (IC_50_ = 4.955/6.802 μM), **12f**/**12e** (IC_50_ = 5.811/7.001 μM) and **12j**/**12i** (IC_50_ = 4.488/7.639 μM), respectively, provide additional support for the correlation. It is also noted that the methylindole-containing compound **12h** (IC_50_ = 3.995 μM) has better anti-PaCa-2 efficacy than the methoxyindolyl analog **12j** (IC_50_ = 4.488 μM). Additionally, the methylindolyl-containing compound has higher anti-PaCa2 properties than the halogen-substituted analogs as seen for **12d**/**12f**/**12g**/**12h** (IC_50_ = 4.955/5.811/5.319/3.995 μM, respectively).

### VEGFR-2 inhibitory properties

VEGFR-2 inhibitory properties of **12a–f** were studied at 10 μM and compared to that of sunitinib (Table 2, Supplementary Fig. S65a,b). Compound **12b** (R = 4-NHCONHPh, R′ = H; % inhibition = 87.2) is the most promising agent synthesized with anti-VEGFR-2 properties close to that of sunitinib (% inhibition = 89.4). Compounds **12c** (R = 3-NHCONHPh, R′ = F) and **12h** (R = 4-NHCONHPh, R′ = Me) also show promising anti-VEGFR-2 properties (both with % inhibition = 85.6).

**Table 2:** % Inhibition of the **12a–j** and sunitinib against VEGFR-2 at 10 μM.

Compd	Relative quantification (RQ)	% Inhibition
**Control**	3.290	0.0
**12a**	0.746	77.3
**12b**	0.422	87.2
**12c**	0.472	85.6
**12d**	0.491	85.1
**12e**	0.538	83.6
**12f**	0.517	84.3
**12g**	0.500	84.8
**12h**	0.475	85.6
**12i**	0.634	80.7
**12j**	0.678	79.4
**Sunitinib**	0.350	89.4

It is manifested from the results that the fluoroindole-containing compounds have higher anti-VEGFR-2 potency than the other halogenated analogs. This is seen in the results for agents **12c**/**12e** (% inhibition = 85.6/83.6) and **12d**/**12f**/**12g** (% inhibition = 85.1/84.3/84.8), respectively. The methylindolyl analog **12h** also has higher anti-VEGFR-2 inhibitory properties than that of the corresponding methoxy-containing compound **12j** (% inhibition = 85.6 and 79.4 respectively). Generally, the anti-VEGFR-2 activities observed are comparable to the antiproliferation properties revealed against cancer cell lines with slight differences due to the applied technique. The antiproliferation properties exhibited are due in-vitro screening in living cancer cells whereas the anti-VEGFR-2 properties are due to biochemical interactions.

### Molecular modeling

Molecular docking of the synthesized agents **12a–j** was investigated using the CDOCKER method (Discovery Studio 2.5 software, Accelrys Inc.)^79^. The anti-VEGFR-2 properties were computationally studied adopting two proteins, PDB: 3WZE and 3AGD^80,81^ co-crystallized with clinically approved anti-angiogenesis drugs; sorafenib and sunitinib, respectively.

### PDB: 3WZE

From the CDOCKER observations (Table 3, Supplementary Fig. S66), it is noted that all the targeted/synthesized agents showed promising interaction docking scores (58.5395–62.4129 kcal mol^−1^) close to that of sorafenib (co-crystallized ligand, 70.6119 kcal mol^−1^). The narrow range of docking score observations is comparable to that shown in anti-VEGFR-2 properties (% inhibition = 77.3–87.2). This can be explained based on the high potency of the synthesized agents with little differences between them due to small substitution difference with the fixed skeletal chemical features being mainly responsible for the revealed bio-properties.

**Table 3:** CDOCKER interaction observations and energy scores (–kcal mol^−1^) of **12a–j** and co-crystallized ligands in the active site of PDB ID: 3WZE and 3AGD.

Compd	PDB: 3WZE		PDB: 3AGD	
	Docking interaction	Docking score	Docking interaction	Docking score
**12a**	H-bonding: urea C=O … ASP1046, 2 urea NH … GLU885 π-cation interaction: phenyl … LYS868 π-σ interaction: indole … LEU840	58.5395	H-bonding: indolyl C=O … CYS919, indolyl NH … GLU917	49.4643
**12b**	H-bonding: urea C=O … ASP1046, 2 urea NH … GLU885, indolyl C=O … CYS919, indolyl NH … CYS919 π-π interaction: indole … PHE918	60.0742	H-bonding: indolyl C=O … CYS919, indolyl NH … GLU917	46.864
**12c**	H-bonding: urea C=O … ASP1046, 2 urea NH … GLU885 π-cation interaction: phenyl … LYS868 π-σ interaction: indole … LEU840	59.5413	H-bonding: indolyl C=O … CYS919, indolyl NH … GLU917	51.2891
**12d**	H-bonding: urea C=O … ASP1046, urea NH … GLU885 π-cation interaction: phenyl … LYS868 π-σ interaction: indole … LEU840	59.2467	H-bonding: ketonic C=O … CYS919, indolyl C=O … ASN923	50.7476
**12e**	H-bonding: urea C=O … ASP1046, 2 urea NH … GLU885 π-cation interaction: phenyl … LYS868 π-σ interaction: indole … LEU840	60.7587	H-bonding: indolyl C=O … CYS919, indolyl NH … GLU917	49.61
**12f**	H-bonding: urea C=O … ASP1046, 2 urea NH … GLU885, indolyl NH … CYS919, indolyl C=O … CYS919	61.4707	H-bonding: indolyl NH … Pro839, urea NH … CYS919 π-σ interaction: phenyl … VAL848	43.1068
**12g**	H-bonding: urea C=O … ASP1046, urea NH … GLU885 π-cation interaction: phenyl … LYS868	61.5089	H-bonding: indolyl NH … CYS919, urea C=O … LYS838, 2 ketonic C=O … ASN923 π-cation interaction: phenyl … LYS838	44.3105
**12h**	H-bonding: urea C=O … ASP1046, urea NH … GLU885 π-cation interaction: phenyl … LYS868	60.744	H-bonding: indolyl C=O … CYS919, indolyl NH … GLU917 π-cation interaction: phenyl … LYS838	47.0129
**12i**	H-bonding: urea C=O … ASP1046, urea NH … GLU885 π-cation interaction: phenyl … LYS868 π-σ interaction: indole … LEU840	61.3951	H-bonding: indolyl NH … CYS919, urea NH … PRO839	42.0334
**12j**	H-bonding: urea C=O … ASP1046, 2 urea NH … GLU885, indolyl C=O … CYS919, indolyl NH … CYS919	62.4129	H-bonding: indolyl C=O … LYS838, ketonic C=O … LYS838, urea NH … CYS919	50.6682
**ligand**^**a**^	H-bonding: urea C=O … ASP1046, 2 urea NH … GLU885 π-cation interaction: phenyl … LYS868	70.6119	H-bonding: indolyl C=O … CYS919, indolyl NH … GLU917	53.2773

^a^Co-crystallized ligands of PDB: 3WZE, 3AGD are sorafenib and sunitinib, respectively.

It is also significant that all the tested compounds have bonding interactions with ASP1046 and GLU885 due to formation of hydrogen bonding with urea C=O and NH respectively. Some of the synthesized agents (**12b**, **12f** and **12j**) also exhibit extra bonding interactions with CYS919 due to hydrogen bonding of indolyl C=O, NH. π-cation and/or π-σ interactions were also revealed by some of the tested analogs (**12a**,**c–e**,**g–i**) with LYS868 and LEU840, respectively. Only compound **12b** showed π-π interaction with PHE918.

It is noted that the *p*-phenylurea-containing compounds (compound **12d** is an exception where it is so close to **12c**) have higher docking score values than the *m*-substituted analogs as revealed by pairs **12b**/**12a** (docking score = 60.0742/58.5395 kcal mol^−1^), **12f**/**12e** (docking score = 61.4707/60.7587 kcal mol^−1^) and **12j**/**12i** (docking score = 62.4129/61.3951 kcal mol^−1^), respectively. This correlation seems to apply generally in most of bio-properties observed (antiproliferation inhibitory properties). The same trend is observed in the VEGFR-2 inhibitory bio-assay. The methoxyindolyl analog **12j** also has a higher docking score than that of the corresponding methyl-containing compound **12h** (docking scores = 62.4129 and 60.744 kcal mol^−1^ respectively).

### PDB: 3AGD

Docking of the synthesized agents **12a–j** in PDB: 3AGD (Table 3, Supplementary Fig. S67), revealed that most of the targeted hits (compounds **12d**, **12f** and **12j** are the exceptions) show the same hydrogen bonding interactions to that of the co-crystallized ligand (sunitinb). The indolyl C=O and NH give hydrogen bonding interactions with CYS919 and GLU917 of the protein active site. Meanwhile, the ketonic C=O is involved in hydrogen interaction with CYS919 for compound **12d**. The urea NH in compounds **12f** and **12j** participates in hydrogen bonding interaction with CYS919. Compound **12c** (R = 3-NHCONHPh, R′ = F) reveals the highest docking score (51.2891 kcal mol^−1^) among the other tested agents, comparable to the co-crystallized ligand (sunitinib, 53.2773 kcal mol^−1^). Compounds **12d** and **12j** also show close docking score values (50.7476, 50.6682 kcal mol^−1^, respectively).

It is noted that, the fluoroindolyl analogs have higher docking score values than the other halogenated agents synthesized as revealed by compounds **12c**/**12e** (51.2891/49.61 kcal mol^−1^) and **12d**/**12f**/**12g** (50.7476/43.1068/44.3105 kcal mol^−1^), respectively. It is also notable that the synthesized methoxyindolyl analog **12j** has a higher docking score than the methylindolyl compound **12h** (50.6682, 47.0129 kcal mol^−1^).

Most of the molecular modeling observations support the exhibited bio-properties. The slight differences between the computational/theoretical studies and the experimental in-vitro (antiproliferation) and biochemical (VEGFR-2) results can be attributed to the fact that differences in the techniques applied can affect the overall outcome. The most fruitful outcome of these studies is that they support the proposed collaboration between the conjugated pharmacophores (indolyl heterocycle and urea function) to enhance antitumor potency.

## Conclusion

A set of 2-oxoindolin-3-ylidenes incorporating the urea function **12a–j** was prepared through a two-step reaction. Addition of the appropriate **9a,b** to the corresponding isatin **10a–f** in ethanol containing a quantitative amount of Et_2_NH, followed by acidic dehydration (AcOH/HCl) afforded the targeted agents in high yields. Some of the synthesized agents show higher efficacies against a group of cancer cell lines (HCT116, MCF7 and PaCa2) than that of sunitinib (clinically usable antitumor drug). VEGFR-2 inhibitory properties are comparable to the antiproferation properties revealed. CDOCKER studies (PDB ID: 3WZE and 3AGD) support the antiproliferation and biochemical observations exhibited. The most fruitful outcome of these studies is the support of assumption due to existence of collaboration between the conjugated pharmacophores (indolyl heterocycle and urea function) in optimizing potent antitumor hits. The current observations can be considered for optimizing more hits/leads that may be considered for more sophisticated pharmacological studies directed towards developing applicable agents.

## Experimental

### Chemical synthesis

Melting points were determined on a capillary point apparatus (Stuart SMP3) equipped with a digital thermometer. IR spectra (KBr) were recorded on a Shimadzu FT-IR 8400S spectrophotometer. Reactions were monitored using thin layer chromatography (TLC) on 0.2 mm silica gel F254 plates (Fluka) utilizing various solvents for elution. The chemical structures of the synthesized compounds were characterized by nuclear magnetic resonance spectra (^1^H-NMR, ^13^C-NMR) and determined on a Bruker NMR spectrometer (500 MHz, 125 MHz for ^1^H and ^13^C, respectively). ^13^C-NMR spectra are fully decoupled. Chemical shifts were reported in parts per million (ppm) using the deuterated solvent peak or tetramethylsilane as an internal standard. Elemental analyses were performed on a Carlo Erba EA-1108 instrument.

### Reaction of 9a,b with 10a–f (general procedure).

A mixture of equimolar amounts (5 mmol) of the appropriate **9a,b** with the corresponding **10a–f** in ethanol (20 ml) containing quantitative amount of diethylamine was stirred at room temperature (20–25 °C). The solid separated was collected, washed with benzene (10 ml) and used without any further purification in the next step for preparation of the targeted agents.

#### 1-{3-[2-(3-Hydroxy-2-oxoindolin-3-yl)acetyl]phenyl}-3-phenylurea (11a)

Obtained from the reaction of **9a** and **10a**, reaction time 24 h as colorless solid, mp 192–194 °C and yield 73% (1.46 g). IR: ν_max_/cm^−1^ 3402, 3306 (OH, NH), 1701, 1682 (C=O), 1636, 1593, 1558. ^1^H-NMR (DMSO-*d*_*6*_) δ (ppm): 3.59 (d, *J* = 17.6 Hz, 1H, upfield H of CH_2_CO), 4.04 (d, *J* = 17.6 Hz, 1H, downfield H of CH_2_CO), 6.10 (s, 1H, OH), 6.83 (d, *J* = 7.7 Hz, 1H, arom. H), 6.87 (t, *J* = 7.5 Hz, 1H, arom. H), 6.98 (t, *J* = 7.4 Hz, 1H, arom. H), 7.18 (t, *J* = 7.2 Hz, 1H, arom. H), 7.28 (d, *J* = 7.7 Hz, 1H, arom. H), 7.30 (d, *J* = 7.7 Hz, 2H, arom. H), 7.41 (t, *J* = 7.9 Hz, 1H, arom. H), 7.48 (d, *J* = 7.7 Hz, 2H, arom. H), 7.53 (d, *J* = 7.8 Hz, 1H, arom. H), 7.66 (d, *J* = 9.2 Hz, 1H, arom. H), 8.03 (s, 1H, arom. H), 8.76 (s, 1H, NH), 8.90 (s, 1H, NH), 10.30 (s, 1H, NH). ^13^C-NMR (DMSO-*d*_*6*_) δ (ppm): 45.7 (CH_2_CO), 72.9 (indolyl C-3), 109.3, 117.1, 118.3, 121.0, 121.4, 121.9, 122.9, 123.5, 128.7, 128.8, 129.0, 129.1, 131.6, 136.7, 139.4, 140.1, 142.9 (arom. C), 152.4, 178.2, 196.1 (CO). Anal. Calcd. for C_23_H_19_N_3_O_4_ (401.42): C, 68.82; H, 4.77; N, 10.47. Found: C, 68.93; H, 4.86; N, 10.65.

#### 1-{4-[2-(3-Hydroxy-2-oxoindolin-3-yl)acetyl]phenyl}-3-phenylurea (11b)

Obtained from the reaction of **9b** and **10a**, reaction time 48 h as colorless solid, mp 198–200 °C and yield 75% (1.50 g). IR: ν_max_/cm^−1^ 3283 (OH, NH), 1709, 1678 (C=O), 1636, 1597, 1562. ^1^H-NMR (DMSO-*d*_*6*_) δ (ppm): 3.51 (d, *J* = 17.3 Hz, 1H, upfield H of CH_2_CO), 3.99 (d, *J* = 17.3 Hz, 1H, downfield H of CH_2_CO), 6.02 (s, 1H, OH), 6.80 (d, *J* = 7.7 Hz, 1H, arom. H), 6.85 (t, *J* = 7.8 Hz, 1H, arom. H), 7.00 (t, *J* = 7.2 Hz, 1H, arom. H), 7.16 (t, *J* = 7.6 Hz, 1H, arom. H), 7.26 (d, *J* = 7.4 Hz, 1H, arom. H), 7.30 (t, *J* = 7.6 Hz, 2H, arom. H), 7.47 (d, *J* = 7.8 Hz, 2H, arom. H), 7.55 (d, *J* = 8.3 Hz, 2H, arom. H), 7.83 (d, *J* = 8.4 Hz, 2H, arom. H), 8.81 (s, 1H, NH), 9.09 (s, 1H, NH), 10.23 (s, 1H, NH). ^13^C-NMR (DMSO-*d*_*6*_) δ (ppm): 45.2 (CH_2_CO), 73.0 (indolyl C-3), 109.2, 116.98, 117.0, 118.3, 120.9, 122.1, 123.4, 128.7, 129.3, 129.6, 131.8, 139.14, 139.17, 142.9, 144.2, 144.4 (arom. C), 152.0, 178.3, 194.6 (CO). Anal. Calcd. for C_23_H_19_N_3_O_4_ (401.42): C, 68.82; H, 4.77; N, 10.47. Found: C, 69.01; H, 4.88; N, 10.55.

#### 1-{3-[2-(5-Fluoro-3-hydroxy-2-oxoindolin-3-yl)acetyl]phenyl}-3-phenylurea (11c)

Obtained from the reaction of **9a** and **10b**, reaction time 48 h as colorless solid, mp 209–211 °C and yield 81% (1.70 g). IR: ν_max_/cm^−1^ 3271 (OH, NH), 1710, 1682 (C=O), 1628, 1597, 1570. ^1^H-NMR (DMSO-*d*_*6*_) δ (ppm): 3.63 (d, *J* = 17.7 Hz, 1H, upfield H of CH_2_CO), 4.09 (d, *J* = 17.7 Hz, 1H, downfield H of CH_2_CO), 6.23 (s, 1H, OH), 6.82 (dd, *J* = 4.3, 8.5 Hz, 1H, arom. H), 6.97–7.03 (m, 2H, arom. H), 7.24 (dd, *J* = 2.6, 8.2 Hz, 1H, arom. H), 7.29 (t, *J* = 7.9 Hz, 2H, arom. H), 7.42 (t, *J* = 7.7 Hz, 1H, arom. H), 7.48 (d, *J* = 7.7 Hz, 2H, arom. H), 7.54 (d, *J* = 7.9 Hz, 1H, arom. H), 7.66 (d, *J* = 9.2 Hz, 1H, arom. H), 8.05 (s, 1H, arom. H), 8.77 (s, 1H, NH), 8.92 (s, 1H, NH), 10.32 (s, 1H, NH). ^13^C-NMR (DMSO-*d*_*6*_) δ (ppm): 45.7 (CH_2_CO), 73.2 (indolyl C-3), 109.90, 109.97, 111.4, 111.6, 114.7, 114.9, 117.1, 118.3, 121.4, 121.9, 123.0, 128.7, 129.05, 129.11, 133.4, 133.5, 136.5, 139.02, 139.03, 139.42, 139.44, 140.08, 140.10, 152.45, 152.48, 156.8 (arom. C), 158.7, 178.2, 196.2 (CO). Anal. Calcd. for C_23_H_18_FN_3_O_4_ (419.41): C, 65.87; H, 4.33; N, 10.02. Found: C, 66.03; H, 4.39; N, 10.19.

#### 1-{4-[2-(5-Fluoro-3-hydroxy-2-oxoindolin-3-yl)acetyl]phenyl}-3-phenylurea (11d)

Obtained from the reaction of **9b** and **10b**, reaction time 24 h as colorless solid, mp 211–213 °C and yield 80% (1.67 g). IR: ν_max_/cm^−1^ 3368, 3283 (OH, NH), 1701, 1667 (C=O), 1589, 1551. ^1^H-NMR (DMSO-*d*_*6*_) δ (ppm): 3.58 (d, *J* = 17.4 Hz, 1H, upfield H of CH_2_CO), 4.05 (d, *J* = 17.5 Hz, 1H, downfield H of CH_2_CO), 6.17 (s, 1H, OH), 6.79–6.82 (m, 1H, arom. H), 6.98–7.02 (m, 2H, arom. H), 7.21 (d, *J* = 9.6 Hz, 1H, arom. H), 7.30 (t, *J* = 7.7 Hz, 2H, arom. H), 7.48 (d, *J* = 7.9 Hz, 2H, arom. H), 7.58 (d, *J* = 8.6 Hz, 2H, arom. H), 7.85 (d, *J* = 8.5 Hz, 2H, arom. H), 8.81 (s, 1H, NH), 9.11 (s, 1H, NH), 10.28 (s, 1H, NH). ^13^C-NMR (DMSO-*d*_*6*_) δ (ppm): 45.3 (CH_2_CO), 73.4 (indolyl C-3), 109.9, 110.0, 111.4, 111.6, 114.7, 114.9, 117.10, 117.14, 118.5, 122.2, 122.3, 128.8, 129.4, 129.61, 129.63, 133.70, 133.76, 139.09, 139.11, 139.2, 139.3, 144.3, 144.6, 152.1, 152.2, 156.9 (arom. C), 158.7, 178.4, 194.7 (CO). Anal. Calcd. for C_23_H_18_FN_3_O_4_ (419.41): C, 65.87; H, 4.33; N, 10.02. Found: C, 66.08; H, 4.50; N, 10.08.

#### 1-{3-[2-(5-Chloro-3-hydroxy-2-oxoindolin-3-yl)acetyl]phenyl}-3-phenylurea (11e)

Obtained from the reaction of **9a** and **10c**, reaction time 24 h as colorless solid, mp 216–218 °C and yield 88% (1.92 g). IR: ν_max_/cm^−1^ 3325, 3264 (OH, NH), 1701, 1682 (C=O), 1651, 1628, 1597, 1558. ^1^H-NMR (DMSO-*d*_*6*_) δ (ppm): 3.63 (d, *J* = 17.9 Hz, 1H, upfield H of CH_2_CO), 4.13 (d, *J* = 17.9 Hz, 1H, downfield H of CH_2_CO), 6.23 (s, 1H, OH), 6.84 (d, *J* = 8.3 Hz, 1H, arom. H), 6.99 (t, *J* = 7.4 Hz, 1H, arom. H), 7.23 (dd, *J* = 2.2, 8.3 Hz, 1H, arom. H), 7.29 (t, *J* = 7.9 Hz, 2H, arom. H), 7.41–7.44 (m, 2H, arom. H), 7.47 (d, *J* = 7.7 Hz, 2H, arom. H), 7.53 (d, *J* = 7.8 Hz, 1H, arom. H), 7.66 (d, *J* = 9.2 Hz, 1H, arom. H), 8.03 (s, 1H, arom. H), 8.74 (s, 1H, NH), 8.89 (s, 1H, NH), 10.43 (s, 1H, NH). ^13^C-NMR (DMSO-*d*_*6*_) δ (ppm): 45.7 (CH_2_CO), 73.0 (indolyl C-3), 110.7, 117.1, 118.3, 121.4, 121.9, 123.0, 123.9, 125.1, 128.5, 128.7, 129.1, 133.8, 136.5, 139.4, 140.1, 141.8 (arom. C), 152.4, 177.9, 196.2 (CO). Anal. Calcd. for C_23_H_18_ClN_3_O_4_ (435.86): C, 63.38; H, 4.16; N, 9.64. Found: C, 63.52; H, 4.28; N, 9.86.

#### 1-{4-[2-(5-Chloro-3-hydroxy-2-oxoindolin-3-yl)acetyl]phenyl}-3-phenylurea (11f)

Obtained from the reaction of **9b** and **10c**, reaction time 24 h as colorless solid, mp 219–221 °C and yield 84% (1.83 g). IR: ν_max_/cm^−1^ 3372, 3283 (OH, NH), 1701, 1667 (C=O), 1616, 1589, 1551. ^1^H-NMR (DMSO-*d*_*6*_) δ (ppm): 3.58 (d, *J* = 17.5 Hz, 1H, upfield H of CH_2_CO), 4.09 (d, *J* = 17.6 Hz, 1H, downfield H of CH_2_CO), 6.19 (s, 1H, OH), 6.83 (d, *J* = 8.0 Hz, 1H, arom. H), 7.00 (t, *J* = 6.7 Hz, 1H, arom. H), 7.22 (d, *J* = 7.5 Hz, 1H, arom. H), 7.30 (t, *J* = 6.9 Hz, 2H, arom. H), 7.39 (s, 1H, arom. H), 7.47 (d, *J* = 7.2 Hz, 2H, arom. H), 7.57 (d, *J* = 8.1 Hz, 2H, arom. H), 7.85 (d, *J* = 8.0 Hz, 2H, arom. H) 8.83 (s, 1H, NH), 9.12 (s, 1H, NH), 10.40 (s, 1H, NH). ^13^C-NMR (DMSO-*d*_*6*_) δ (ppm): 45.3 (CH_2_CO), 73.1 (indolyl C-3), 110.7, 117.1, 118.4, 122.2, 123.9, 125.1, 128.5, 128.8, 129.4, 129.5, 129.6, 134.1, 139.2, 139.3, 141.9, 144.6 (arom. C), 152.1, 178.1, 194.7 (CO). Anal. Calcd. for C_23_H_18_ClN_3_O_4_ (435.86): C, 63.38; H, 4.16; N, 9.64. Found: C, 63.55; H, 4.26; N, 9.70.

#### 1-{4-[2-(5-Bromo-3-hydroxy-2-oxoindolin-3-yl)acetyl]phenyl}-3-phenylurea (11g)

Obtained from the reaction of **9b** and **10d**, reaction time 48 h as colorless solid, mp 208–210 °C and yield 77% (1.85 g). IR: ν_max_/cm^−1^ 3225 (OH, NH), 1678, 1616 (C=O), 1593, 1543. ^1^H-NMR (DMSO-*d*_*6*_) δ (ppm): 3.57 (d, *J* = 17.7 Hz, 1H, upfield H of CH_2_CO), 4.08 (d, *J* = 17.7 Hz, 1H, downfield H of CH_2_CO), 6.17 (s, 1H, OH), 6.78 (d, *J* = 8.2 Hz, 1H, arom. H), 7.00 (t, *J* = 7.2 Hz, 1H, arom. H), 7.30 (t, *J* = 7.6 Hz, 2H, arom. H), 7.35 (d, *J* = 8.2 Hz, 1H, arom. H), 7.47 (s, 1H, arom. H), 7.49 (d, *J* = 6.7 Hz, 2H, arom. H), 7.57 (d, *J* = 8.3 Hz, 2H, arom. H), 7.84 (d, *J* = 8.2 Hz, 2H, arom. H) 8.83 (s, 1H, NH), 9.13 (s, 1H, NH), 10.38 (s, 1H, NH). ^13^C-NMR (DMSO-*d*_*6*_) δ (ppm): 45.2 (CH_2_CO), 73.0 (indolyl C-3), 111.2, 112.7, 117.0, 118.4, 122.1, 126.5, 128.7, 129.4, 131.3, 134.4, 139.1, 142.2, 144.6 (arom. C), 152.0, 177.9, 194.7 (CO). Anal. Calcd. for C_23_H_18_BrN_3_O_4_ (480.32): C, 57.51; H, 3.78; N, 8.75. Found: C, 57.73; H, 3.70; N, 8.86.

#### 1-{4-[2-(3-Hydroxy-5-methyl-2-oxoindolin-3-yl]acetyl}phenyl)-3-phenylurea (11h)

Obtained from the reaction of **9b** and **10e**, reaction time 48 h as colorless solid, mp 213–215 °C and yield 80% (1.65 g). IR: ν_max_/cm^−1^ 3368, 3287 (OH, NH), 1694, 1667 (C=O), 1624, 1589, 1551. ^1^H-NMR (DMSO-*d*_*6*_) δ (ppm): 2.18 (s, 3H, ArCH_3_), 3.53 (d, *J* = 17.4 Hz, 1H, upfield H of CH_2_CO), 3.97 (d, *J* = 17.4 Hz, 1H, downfield H of CH_2_CO), 6.00 (s, 1H, OH), 6.70 (d, *J* = 7.7 Hz, 1H, arom. H), 6.96 (d, *J* = 7.7 Hz, 1H, arom. H), 7.00 (t, *J* = 7.2 Hz, 1H, arom. H), 7.09 (s, 1H, arom. H), 7.30 (t, *J* = 7.4 Hz, 2H, arom. H), 7.48 (d, *J* = 7.5 Hz, 2H, arom. H), 7.57 (d, *J* = 8.2 Hz, 2H, arom. H), 7.85 (d, *J* = 8.2 Hz, 2H, arom. H), 8.82 (s, 1H, NH), 9.11 (s, 1H, NH), 10.15 (s, 1H, NH). ^13^C-NMR (DMSO-*d*_*6*_) δ (ppm): 20.6 (ArCH_3_), 45.4 (CH_2_CO), 73.2 (indolyl C-3), 109.1, 117.08, 117.13, 118.4, 122.21, 122.23, 124.2, 128.8, 128.9, 129.4, 129.6, 129.72, 129.75, 131.9, 139.2, 139.3, 140.5, 144.5 (arom. C), 152.1, 178.4, 194.6 (CO). Anal. Calcd. for C_24_H_21_N_3_O_4_ (415.45): C, 69.39; H, 5.10; N, 10.11. Found: C, 69.23; H, 4.96; N, 10.04.

#### 1-{3-[2-(3-Hydroxy-5-methoxy-2-oxoindolin-3-yl)acetyl]phenyl}-3-phenylurea (11i)

Obtained from the reaction of **9a** and **10f**, reaction time 48 h as colorless solid, mp 202–204 °C and yield 90% (1.93 g). IR: ν_max_/cm^−1^ 3302 (OH, NH), 1694 (C=O), 1639, 1597, 1566. ^1^H-NMR (DMSO-*d*_*6*_) δ (ppm): 3.59 (d, *J* = 17.5 Hz, 1H, upfield H of CH_2_CO), 3.65 (s, 3H, OCH_3_), 4.06 (d, *J* = 17.5 Hz, 1H, downfield H of CH_2_CO), 6.12 (s, 1H, OH), 6.75 (s, 2H, arom. H), 6.97–7.00 (m, 2H, arom. H), 7.30 (t, *J* = 7.8 Hz, 2H, arom. H), 7.42 (t, *J* = 7.9 Hz, 1H, arom. H), 7.49 (d, *J* = 8.0 Hz, 2H, arom. H), 7.54 (d, *J* = 7.7 Hz, 1H, arom. H), 7.66 (d, *J* = 8.1 Hz, 1H, arom. H), 8.05 (s, 1H, arom. H), 8.75 (s, 1H, NH), 8.90 (s, 1H, NH), 10.14 (s, 1H, NH). ^13^C-NMR (DMSO-*d*_*6*_) δ (ppm): 45.7 (CH_2_CO), 55.3 (OCH_3_), 73.4 (indolyl C-3), 109.6, 110.9, 113.3, 117.1, 118.3, 121.4, 121.9, 122.9, 128.7, 129.06, 129.08, 132.8, 136.0, 136.7, 139.4, 140.1, 152.4, 152.5 (arom. C), 154.5, 178.2, 196.1 (CO). Anal. Calcd. for C_24_H_21_N_3_O_5_ (431.45): C, 66.81; H, 4.91; N, 9.74. Found: C, 66.86; H, 5.04; N, 9.58.

#### 1-{4-[2-(3-Hydroxy-5-methoxy-2-oxoindolin-3-yl)acetyl]phenyl}-3-phenylurea (11j)

Obtained from the reaction of **9b** and **10f**, reaction time 24 h as colorless solid, mp 208–210 °C and yield 91% (1.96 g). IR: ν_max_/cm^−1^ 3364, 3279 (OH, NH), 1697, 1667 (C=O), 1589, 1547. ^1^H-NMR (DMSO-*d*_*6*_) δ (ppm): 3.51 (d, *J* = 17.2 Hz, 1H, upfield H of CH_2_CO), 3.64 (s, 3H, OCH_3_), 4.01 (d, *J* = 17.3 Hz, 1H, downfield H of CH_2_CO), 6.04 (s, 1H, OH), 6.72 (s, 2H, arom. H), 6.95 (s, 1H, arom. H), 7.00 (t, *J* = 7.3 Hz, 1H, arom. H), 7.30 (t, *J* = 7.7 Hz, 2H, arom. H), 7.47 (d, *J* = 7.8 Hz, 2H, arom. H), 7.56 (d, *J* = 8.6 Hz, 2H, arom. H), 7.85 (d, *J* = 8.5 Hz, 2H, arom. H), 8.82 (s, 1H, NH), 9.11 (s, 1H, NH), 10.08 (s, 1H, NH). ^13^C-NMR (DMSO-*d*_*6*_) δ (ppm): 45.2 (CH_2_CO), 55.3 (OCH_3_), 73.4 (indolyl C-3), 109.5, 110.8, 113.1, 116.98, 117.03, 118.4, 122.1, 128.7, 129.3, 129.5, 129.7, 133.0, 136.0, 139.1, 139.2, 144.3, 144.4, 152.02, 152.06 (arom. C), 154.5, 178.2, 194.6 (CO). Anal. Calcd. for C_24_H_21_N_3_O_5_ (431.45): C, 66.81; H, 4.91; N, 9.74. Found: C, 66.70; H, 4.82; N, 9.63.

### Acidic dehydration of 11a–j (general procedure).

A solution of the appropriate **11a–j** (2.5 mmol) in glacial acetic acid (20 ml) containing hydrochloric acid (2 ml, 35%) was stirred at room temperature for the appropriate time. The separated solid was collected, washed with water and crystallized from a suitable solvent affording the corresponding **12a–j**.

#### (*E*)-1-{3-[2-(2-Oxoindolin-3-ylidene)acetyl]phenyl}-3-phenylurea (12a)

Obtained from acidic dehydration of **11a**, reaction time 48 h, orange crystals from n-butanol, mp 227–229 °C and yield 90% (0.86 g). IR: ν_max_/cm^−1^ 3341, 3314 (NH), 1724, 1690 (C=O), 1651, 1593, 1555. ^1^H-NMR (DMSO-*d*_*6*_) δ (ppm): 6.89 (d, *J* = 7.8 Hz, 1H, arom. H), 6.94–7.00 (m, 2H, arom. H), 7.29 (t, *J* = 7.9 Hz, 2H, arom. H), 7.34 (t, *J* = 7.7 Hz, 1H, arom. H), 7.48 (d, *J* = 9.0 Hz, 2H, arom. H), 7.52 (d, *J* = 7.9 Hz, 1H, arom. H), 7.68 (d, *J* = 8.1 Hz, 1H, arom. H), 7.71 (s, 1H, olefinic CH), 7.76 (d, *J* = 8.1 Hz, 1H, arom. H), 8.04 (d, *J* = 7.8 Hz, 1H, arom. H), 8.24 (s, 1H, arom. H), 8.73 (s, 1H, NH), 9.02 (s, 1H, NH), 10.81 (s, 1H, NH). ^13^C-NMR (DMSO-*d*_*6*_) δ (ppm): 110.3, 117.6, 118.4, 119.9, 121.7, 121.9, 122.0, 123.5, 125.9, 126.6, 128.7, 129.6, 132.8, 136.3, 137.6, 139.4, 140.5, 144.9 (arom. C + olefinic C), 152.5, 168.1, 191.0 (CO). Anal. Calcd. for C_23_H_17_N_3_O_3_ (383.41): C, 72.05; H, 4.47; N, 10.96. Found: C, 72.21; H, 4.55; N, 11.00.

#### (*E*)-1-{4-[2-(2-Oxoindolin-3-ylidene)acetyl]phenyl}-3-phenylurea (12b)

Obtained from acidic dehydration of **11b**, reaction time 24 h, red crystals from N,N-dimethylformamide (DMF) (80%), mp 259–261 °C and yield 91% (1.29 g). IR: ν_max_/cm^−1^ 3302 (NH), 1709 (C=O), 1647, 1585, 1555. ^1^H-NMR (DMSO-*d*_*6*_) δ (ppm): 6.89 (d, *J* = 7.7 Hz, 1H, arom. H), 6.95 (t, *J* = 7.3 Hz, 1H, arom. H), 7.02 (t, *J* = 7.3 Hz, 1H, arom. H), 7.30–7.35 (m, 3H, arom. H), 7.49 (d, *J* = 7.7 Hz, 2H, arom. H), 7.68 (d, *J* = 8.8 Hz, 2H, arom. H), 7.71 (s, 1H, olefinic CH), 7.99 (d, *J* = 7.7 Hz, 1H, arom. H), 8.05 (d, *J* = 8.8 Hz, 2H, arom. H), 8.85 (s, 1H, NH), 9.26 (s, 1H, NH), 10.79 (s, 1H, NH). ^13^C-NMR (DMSO-*d*_*6*_) δ (ppm): 110.2, 117.4, 118.4, 120.0, 121.6, 122.2, 126.5, 126.6, 128.7, 130.2, 130.4, 132.4, 135.5, 139.1, 144.6, 145.3 (arom. C + olefinic C), 152.0, 168.1, 189.4 (CO). Anal. Calcd. for C_23_H_17_N_3_O_3_ (383.41): C, 72.05; H, 4.47; N, 10.96. Found: C, 72.16; H, 4.61; N, 11.15.

#### (*E*)-1-{3-[2-(5-Fluoro-2-oxoindolin-3-ylidene)acetyl]phenyl}-3-phenylurea (12c)

Obtained from acidic dehydration of **11c**, reaction time 72 h, red crystals from n-butanol, mp 209–211 °C and yield 89% (0.89 g) IR: ν_max_/cm^−1^ 3283, 3198 (NH), 1713, 1659 (C=O), 1589, 1555. ^1^H-NMR (DMSO-*d*_*6*_) δ (ppm): 6.90 (dd, *J* = 4.5, 8.6 Hz, 1H, arom. H), 7.00 (t, *J* = 7.5 Hz, 1H, arom. H), 7.25 (dt, *J* = 2.7, 9.0 Hz, 1H, arom. H), 7.30 (t, *J* = 7.8 Hz, 2H, arom. H), 7.49 (d, *J* = 8.0 Hz, 2H, arom. H), 7.53 (t, *J* = 7.9 Hz, 1H, arom. H), 7.70 (d, *J* = 7.8 Hz, 1H, arom. H), 7.76 (dd, *J* = 2.1, 8.1 Hz, 1H, arom. H), 7.79 (s, 1H, olefinic CH), 7.92 (dd, *J* = 2.7, 9.5 Hz, 1H, arom. H), 8.26 (d, *J* = 2.3 Hz, 1H, arom. H), 8.75 (s, 1H, NH), 9.04 (s, 1H, NH), 10.86 (s, 1H, NH). ^13^C-NMR (DMSO-*d*_*6*_) δ (ppm): 111.17, 111.24, 113.6, 113.8, 117.7, 118.4, 119.3, 119.5, 120.76, 120.83, 122.1, 123.7, 126.9, 128.77, 128.78, 129.7, 136.36, 136.39, 137.6, 139.4, 140.6, 141.43, 141.44, 152.5, 156.4 (arom. C + olefinic C), 158.3, 168.2, 190.7 (CO). Anal. Calcd. for C_23_H_16_FN_3_O_3_ (401.40): C, 68.82; H, 4.02; N, 10.47. Found: C, 68.76; H, 3.91; N, 10.61.

#### (*E*)-1-{4-[2-(5-Fluoro-2-oxoindolin-3-ylidene)acetyl]phenyl}-3-phenylurea (12d)

Obtained from acidic dehydration of **11d**, reaction time 24 h, red crystals from n-butanol, mp 245–247 °C and yield 94% (0.94 g). IR: ν_max_/cm^−1^ 3294, 3175 (NH), 1709, 1651 (C=O), 1585, 1555. ^1^H-NMR (DMSO-*d*_*6*_) δ (ppm): 6.88 (dd, *J* = 4.5, 8.5 Hz, 1H, arom. H), 7.03 (t, *J* = 7.3 Hz, 1H, arom. H), 7.21 (dt, *J* = 2.5, 8.9 Hz, 1H, arom. H), 7.33 (t, *J* = 7.8 Hz, 2H, arom. H), 7.52 (d, *J* = 7.9 Hz, 2H, arom. H), 7.71 (d, *J* = 8.7 Hz, 2H, arom. H), 7.82 (s, 1H, olefinic CH), 7.95 (dd, *J* = 2.3, 9.4 Hz, 1H, arom. H), 8.08 (d, *J* = 8.7 Hz, 2H, arom. H), 8.87 (s, 1H, NH), 9.28 (s, 1H, NH), 10.83 (s, 1H, NH). ^13^C-NMR (DMSO-*d*_*6*_) δ (ppm): 110.9, 111.0, 113.7, 113.9, 117.5, 118.5, 118.9, 119.1, 120.9, 121.0, 122.3, 127.2, 128.8, 130.3, 130.5, 135.81, 135.83, 139.2, 141.2, 145.4, 152.0, 156.4 (arom. C + olefinic C), 158.3, 168.3, 188.8 (CO). Anal. Calcd. for C_23_H_16_FN_3_O_3_ (401.40): C, 68.82; H, 4.02; N, 10.47. Found: C, 68.96; H, 4.09; N, 10.53.

#### (*E*)-1-{3-[2-(5-Chloro-2-oxoindolin-3-ylidene)acetyl]phenyl}-3-phenylurea (12e)

Obtained from acidic dehydration of **11e**, reaction time 72 h, red crystals from n-butanol, mp 242–244 °C and yield 78% (0.81 g). IR: ν_max_/cm^−1^ 3291, 3163 (NH), 1717, 1651 (C=O), 1589, 1558. ^1^H-NMR (DMSO-*d*_*6*_) δ (ppm): 6.92 (d, *J* = 8.4 Hz, 1H, arom. H), 7.00 (t, *J* = 7.4 Hz, 1H, arom. H), 7.31 (t, *J* = 7.9 Hz, 2H, arom. H), 7.43 (dd, *J* = 2.1, 8.3 Hz, 1H, arom. H), 7.49 (d, *J* = 7.8 Hz, 2H, arom. H), 7.53 (t, *J* = 7.9 Hz, 1H, arom. H), 7.71 (d, *J* = 7.7 Hz, 1H, arom. H), 7.76 (d, *J* = 7.8 Hz, 1H, arom. H), 7.80 (s, 1H, olefinic CH), 8.16 (d, *J* = 1.9 Hz, 1H, arom. H), 8.27 (s, 1H, arom. H), 8.76 (s, 1H, NH), 9.04 (s, 1H, NH), 10.98 (s, 1H, NH). ^13^C-NMR (DMSO-*d*_*6*_) δ (ppm): 112.3, 118.2, 118.9, 121.9, 122.5, 124.2, 126.0, 126.8, 127.5, 129.3, 130.1, 132.9, 136.3, 138.0, 139.9, 141.1, 144.3 (arom. C + olefinic C), 153.0, 168.4, 191.2 (CO). Anal. Calcd. for C_23_H_16_ClN_3_O_3_ (417.85): C, 66.11; H, 3.86; N, 10.06. Found: C, 65.91; H, 3.68; N, 9.90.

#### (*E*)-1-{4-[2-(5-Chloro-2-oxoindolin-3-ylidene)acetyl]phenyl}-3-phenylurea (12f)

Obtained from acidic dehydration of **11f**, reaction time 48 h, red crystals from DMF (80%), mp 241–243 °C and yield 87% (0.90 g). IR: ν_max_/cm^−1^ 3348, 3175 (NH), 1717, 1659 (C=O), 1589, 1547. ^1^H-NMR (DMSO-*d*_*6*_) δ (ppm): 6.90 (dd, *J* = 2.2, 8.3 Hz, 1H, arom. H), 7.02 (t, *J* = 7.4 Hz, 1H, arom. H), 7.31 (t, *J* = 7.9 Hz, 2H, arom. H), 7.40 (d, *J* = 8.3 Hz, 1H, arom. H), 7.49 (d, *J* = 8.3 Hz, 2H, arom. H), 7.68 (dd, *J* = 1.6, 8.8 Hz, 2H, arom. H), 7.81 (d, *J* = 1.6 Hz, 1H, arom. H), 7.96 (s, 1H, olefinic CH), 8.07 (d, *J* = 1.5 Hz, 1H, arom. H), 8.13 (d, *J* = 2.0 Hz, 1H, arom. H), 8.86 (s, 1H, NH), 9.28 (s, 1H, NH), 10.92 (s, 1H, NH). ^13^C-NMR (DMSO-*d*_*6*_) δ (ppm): 111.6, 117.4, 118.4, 121.5, 122.3, 125.4, 126.3, 127.6, 128.7, 130.4, 132.0, 135.1, 139.1, 143.5, 145.4 (arom. C + olefinic C), 151.9, 167.9, 188.8 (CO). Anal. Calcd. for C_23_H_16_ClN_3_O_3_ (417.85): C, 66.11; H, 3.86; N, 10.06. Found: C, 66.00; H, 3.81; N, 10.02.

#### (*E*)-1-{4-[2-(5-Bromo-2-oxoindolin-3-ylidene)acetyl]phenyl}-3-phenylurea (12g)

Obtained from acidic dehydration of **11g**, reaction time 72 h, red crystals from n-butanol, mp 249–251 °C and yield 93% (1.07 g). IR: ν_max_/cm^−1^ 3310, 3167 (NH), 1717, 1659 (C=O), 1593, 1551. ^1^H-NMR (DMSO-*d*_*6*_) δ (ppm): 6.87 (d, *J* = 8.4 Hz, 1H, arom. H), 7.02 (t, *J* = 7.4 Hz, 1H, arom. H), 7.32 (t, *J* = 7.7 Hz, 2H, arom. H), 7.49 (d, *J* = 8.0 Hz, 2H, arom. H), 7.54 (dd, *J* = 2.1, 8.3 Hz, 1H, arom. H), 7.68 (d, *J* = 8.5 Hz, 2H, arom. H), 7.81 (s, 1H, olefinic CH), 8.07 (d, *J* = 8.5 Hz, 2H, arom. H), 8.27 (d, *J* = 2.2 Hz, 1H, arom. H), 8.86 (s, 1H, NH), 9.27 (s, 1H, NH), 10.94 (s, 1H, NH). ^13^C-NMR (DMSO-*d*_*6*_) δ (ppm): 112.2, 113.2, 117.5, 118.5, 122.0, 122.3, 127.7, 128.8, 129.1, 130.4, 130.5, 134.9, 135.0, 139.1, 144.0, 145.5 (arom. C + olefinic C), 152.0, 167.8, 188.9 (CO). Anal. Calcd. for C_23_H_16_BrN_3_O_3_ (462.30): C, 59.76; H, 3.49; N, 9.09. Found: C, 59.89; H, 3.38; N, 9.03.

#### (*E*)-1-{4-[2-(5-Methyl-2-oxoindolin-3-ylidene)acetyl]phenyl}-3-phenylurea (12h)

Obtained from acidic dehydraation of **11h**, reaction time 72 h, orange crystals from n-butanol, mp 226–228 °C and yield 82% (0.81 g). IR: ν_max_/cm^−1^ 3267 (NH), 1713, 1655 (C=O), 1589, 1535. ^1^H-NMR (DMSO-*d*_*6*_) δ (ppm): 2.23 (s, 3H, ArCH_3_), 6.78 (d, *J* = 7.9 Hz, 1H, arom. H), 7.02 (t, *J* = 7.4 Hz, 1H, arom. H), 7.15 (d, *J* = 7.9 Hz, 1H, arom. H), 7.31 (t, *J* = 7.9 Hz, 2H, arom. H), 7.49 (d, *J* = 7.8 Hz, 2H, arom. H), 7.68 (d, *J* = 8.8 Hz, 2H, arom. H), 7.69 (s, 1H, arom. H), 7.87 (s, 1H, olefinic CH), 8.04 (d, *J* = 8.8 Hz, 2H, arom. H), 8.83 (s, 1H, NH), 9.23 (s, 1H, NH), 10.66 (s, 1H, NH). ^13^C-NMR (DMSO-*d*_*6*_) δ (ppm): 20.6 (ArCH_3_), 109.9, 117.4, 118.4, 120.1, 122.2, 126.1, 127.1, 128.7, 130.2, 130.3, 130.5, 132.9, 136.0, 139.1, 142.4, 145.2 (arom. C + olefinic C), 152.0, 168.2, 189.3 (CO). Anal. Calcd. for C_24_H_19_N_3_O_3_ (397.43): C, 72.53; H, 4.82; N, 10.57. Found: C, 72.74; H, 4.89; N, 10.43.

#### (*E*)-1-{3-[2-(5-Methoxy-2-oxoindolin-3-ylidene)acetyl]phenyl}-3-phenylurea (12i)

Obtained from acidic dehydration of **11i**, reaction time 72 h, red crystals from n-butanol, mp 234–236 °C and yield 87% (0.90 g). IR: ν_max_/cm^−1^ 3267, 3190 (NH), 1713, 1651 (C=O), 1589, 1555. ^1^H-NMR (DMSO-*d*_*6*_) δ (ppm): 3.71 (s, 3H, OCH_3_), 6.81 (d, *J* = 8.5 Hz, 1H, arom. H), 6.97 (dd, *J* = 2.4, 8.5 Hz, 1H, arom. H), 7.00 (d, *J* = 7.4 Hz, 1H, arom. H), 7.30 (t, *J* = 7.9 Hz, 2H, arom. H), 7.48 (d, *J* = 7.8 Hz, 2H, arom. H), 7.52 (t, *J* = 7.9 Hz, 1H, arom. H), 7.69 (d, *J* = 9.9 Hz, 1H, arom. H), 7.70 (s, 1H, olefinic CH), 7.71 (d, *J* = 2.5 Hz, 1H, arom. H), 7.75 (d, *J* = 9.2 Hz, 1H, arom. H), 8.25 (s, 1H, arom. H), 8.73 (s, 1H, NH), 9.03 (s, 1H, NH), 10.63 (s, 1H, NH). ^13^C-NMR (DMSO-*d*_*6*_) δ (ppm): 55.4 (OCH_3_), 110.7, 112.7, 113.8, 117.6, 118.4, 120.5, 121.90, 121.98, 123.5, 125.9, 128.7, 129.6, 136.90, 136.94, 137.6, 138.6, 139.4, 140.5, 152.4 (arom. C + olefinic C), 154.3, 168.1, 191.0 (CO). Anal. Calcd. for C_24_H_19_N_3_O_4_ (413.43): C, 69.72; H, 4.63; N, 10.16. Found: C, 69.63; H, 4.50; N, 10.09.

#### (*E*)-1-{4-[2-(5-Methoxy-2-oxoindolin-3-ylidene)acetyl]phenyl}-3-phenylurea (12j)

Obtained from acidic dehydration of **11j**, reaction time 24 h, brownish red crystals from DMF (80%), mp 235–237 °C and yield 97% (1.00 g). IR: ν_max_/cm^−1^ 3325, 3287 (NH), 1717, 1655 (C=O), 1593, 1547. ^1^H-NMR (DMSO-*d*_*6*_) δ (ppm): 3.68 (s, 3H, OCH_3_), 6.80 (d, *J* = 8.5 Hz, 1H, arom. H), 6.95 (dd, *J* = 2.6, 8.5 Hz, 1H, arom. H), 7.01 (t, *J* = 7.4 Hz, 1H, arom. H), 7.31 (t, *J* = 7.9 Hz, 2H, arom. H), 7.48 (d, *J* = 7.7 Hz, 2H, arom. H), 7.65 (d, *J* = 2.6 Hz, 1H, arom. H), 7.67 (d, *J* = 8.9 Hz, 2H, arom. H), 7.70 (s, 1H, olefinic CH), 8.04 (d, *J* = 8.8 Hz, 2H, arom. H), 8.84 (s, 1H, NH), 9.24 (s, 1H, NH), 10.59 (s, 1H, NH). ^13^C-NMR (DMSO-*d*_*6*_) δ (ppm): 55.4 (OCH_3_), 110.6, 112.7, 117.4, 117.9, 118.4, 120.7, 122.2, 126.6, 128.7, 130.2, 130.4, 136.1, 138.4, 139.1, 145.3, 151.9 (arom. C + olefinic C), 154.3, 168.2, 189.4 (CO). Anal. Calcd. for C_24_H_19_N_3_O_4_ (413.43): C, 69.72; H, 4.63; N, 10.16. Found: C, 69.58; H, 4.54; N, 10.01.

### X-ray, antiproliferation, VEGFR-2 inhibitory properties and molecular modeling studies

Are mentioned in the supplementary file.

## Supplementary Information

Supplementary Information 1.

Supplementary Information 2.

## Data Availability

All data generated or analyzed during this study are included in this published article and its supplementary material files. The X-ray data have been deposited in the CSD with reference number CCDC 2333946 and the Check-CIF file is also attached as supplementary file to this article.
