# Supplementary material for: Design and synthesis of antiproliferative 2-oxoindolin-3-ylidenes incorporating urea function with potential VEGFR-2 inhibitory properties
Source: Sci Rep. 2025 Jan 3;15:618. doi: 10.1038/s41598-024-82005-6 (PMC11699130; doi:10.1038/s41598-024-82005-6)

checkCIF/PLATON report

Structure factors have been supplied for datablock(s) bmk2355

THIS REPORT IS FOR GUIDANCE ONLY. IF USED AS PART OF A REVIEW PROCEDURE FOR PUBLICATION, IT SHOULD NOT REPLACE THE EXPERTISE OF AN EXPERIENCED CRYSTALLOGRAPHIC REFEREE.

No syntax errors found. CIF dictionary Interpreting this report

Datablock: bmk2355

Bond precision:	C-C = 0.0176 A		Wavelength=0.71073
Cell:	a=9.2245(9)	b=12.9911(11)	c=30.476(3)
	alpha=90	beta=90	gamma=90
Temperature:	293 K		

	Calculated	Reported
Volume	3652.1(6)	3652.2(6)
Space group	P n a 21	P n a 21
Hall group	P 2c -2n	P 2c -2n
Moiety formula	C23 H17 N3 O3	C23 H17 N3 O3
Sum formula	C23 H17 N3 O3	C23 H17 N3 O3
Mr	383.40	383.39
Dx,g cm-3	1.395	1.395
Z	8	8
Mu (mm-1)	0.095	0.094
F000	1600.0	1600.0
F000’	1600.71	
h,k,lmax	12,18,42	12,17,42
Nref	10546[5365]	7604
Tmin,Tmax	0.993,0.997	0.811,1.000
Tmin’	0.978	

Correction method= # Reported T Limits: Tmin=0.811 Tmax=1.000
AbsCorr = GAUSSIAN

Data completeness= 1.42/0.72 Theta(max)= 29.885

R(reflections)= 0.0777(2702) wR2(reflections)= 0.2238(7604)

S = 0.971 Npar= 524

The following ALERTS were generated. Each ALERT has the format
test-name_ALERT_alert-type_alert-level.
Click on the hyperlinks for more details of the test.

Alert level B

```
PLAT026_ALERT_3_B Ratio Observed / Unique Reflections (too) Low ..        36% Check
PLAT340_ALERT_3_B Low Bond Precision on  C-C Bonds ..............    0.01757 Ang.
```

Alert level C

```
STRVA01_ALERT_4_C           Flack parameter is too small
           From the CIF: _refine_ls_abs_structure_Flack   -2.200
           From the CIF: _refine_ls_abs_structure_Flack_su    1.000
PLAT031_ALERT_4_C Refined Extinction Parameter Within Range of ...      2.500 Sigma
PLAT234_ALERT_4_C Large Hirshfeld Difference C6       --C7       .       0.17 Ang.
PLAT234_ALERT_4_C Large Hirshfeld Difference C7       --C8       .       0.17 Ang.
PLAT234_ALERT_4_C Large Hirshfeld Difference C20      --C21      .       0.20 Ang.
PLAT234_ALERT_4_C Large Hirshfeld Difference C21      --C22      .       0.23 Ang.
PLAT234_ALERT_4_C Large Hirshfeld Difference O6       --C40      .       0.18 Ang.
PLAT234_ALERT_4_C Large Hirshfeld Difference C25      --C26      .       0.16 Ang.
PLAT241_ALERT_2_C High   'MainMol' Ueq as Compared to Neighbors of        C22 Check
PLAT242_ALERT_2_C Low    'MainMol' Ueq as Compared to Neighbors of        C18 Check
PLAT331_ALERT_2_C Small Aver Phenyl C-C Dist C18      --C23      .       1.37 Ang.
PLAT334_ALERT_2_C Small <C-C> Benzene Dist.   C11      -C16      .       1.36 Ang.
PLAT334_ALERT_2_C Small <C-C> Benzene Dist.   C26      -C31      .       1.37 Ang.
PLAT369_ALERT_2_C Long   C(sp2)-C(sp2) Bond  C10      - C11      .       1.53 Ang.
PLAT369_ALERT_2_C Long   C(sp2)-C(sp2) Bond  C24      - C25      .       1.53 Ang.
PLAT905_ALERT_3_C Negative K value in the Analysis of Variance ...     -6.160 Report
PLAT905_ALERT_3_C Negative K value in the Analysis of Variance ...     -1.050 Report
PLAT910_ALERT_3_C Missing # of FCF Reflection(s) Below Theta(Min).          8 Note
                1  1  0,   0  2  0,   0  1  1,   1  1  1,   0  0  2,   1  1  2,
                0  1  3,   0  0  4,
```

Alert level G

```
PLAT003_ALERT_2_G Number of Uiso or Uij Restrained non-H Atoms ...          2 Report
PLAT007_ALERT_5_G Number of Unrefined Donor-H Atoms .............           6 Report
              H1    H2    H3    H4    H5A   H6A
PLAT032_ALERT_4_G Std. Uncertainty on Flack Parameter Value High .      1.000 Report
PLAT186_ALERT_4_G The CIF-Embedded .res File Contains ISOR Records          1 Report
PLAT199_ALERT_1_G Reported _cell_measurement_temperature ..... (K)        293 Check
PLAT200_ALERT_1_G Reported   _diffrn_ambient_temperature ..... (K)        293 Check
PLAT860_ALERT_3_G Number of Least-Squares Restraints .............         13 Note
PLAT883_ALERT_1_G No Info/Value for _atom_sites_solution_primary .     Please Do !
PLAT899_ALERT_4_G SHELXL2018 is Deprecated and Succeeded by SHELXL     2019/3 Note
PLAT912_ALERT_4_G Missing # of FCF Reflections Above STh/L=  0.600        678 Note
PLAT915_ALERT_3_G No Flack x Check Done: Low Friedel Pair Coverage         57 %
PLAT916_ALERT_2_G Hooft y and Flack x Parameter Values Differ by .       1.80 Check
PLAT941_ALERT_3_G Average HKL Measurement Multiplicity ..........         3.6 Low
PLAT969_ALERT_5_G The 'Henn et al.' R-Factor-gap value ..........        1.63 Note
              Predicted wR2: Based on SigI**2 13.74 or SHELX Weight 23.88
PLAT978_ALERT_2_G Number C-C Bonds with Positive Residual Density.          0 Info
```

```
 0 ALERT level A = Most likely a serious problem - resolve or explain
 2 ALERT level B = A potentially serious problem, consider carefully
18 ALERT level C = Check. Ensure it is not caused by an omission or oversight
15 ALERT level G = General information/check it is not something unexpected

 3 ALERT type 1 CIF construction/syntax error, inconsistent or missing data
10 ALERT type 2 Indicator that the structure model may be wrong or deficient
 8 ALERT type 3 Indicator that the structure quality may be low
12 ALERT type 4 Improvement, methodology, query or suggestion
 2 ALERT type 5 Informative message, check
```

It is advisable to attempt to resolve as many as possible of the alerts in all categories. Often the minor alerts point to easily fixed oversights, errors and omissions in your CIF or refinement strategy, so attention to these fine details can be worthwhile. In order to resolve some of the more serious problems it may be necessary to carry out additional measurements or structure refinements. However, the purpose of your study may justify the reported deviations and the more serious of these should normally be commented upon in the discussion or experimental section of a paper or in the "special_details" fields of the CIF. checkCIF was carefully designed to identify outliers and unusual parameters, but every test has its limitations and alerts that are not important in a particular case may appear. Conversely, the absence of alerts does not guarantee there are no aspects of the results needing attention. It is up to the individual to critically assess their own results and, if necessary, seek expert advice.

Publication of your CIF in IUCr journals

A basic structural check has been run on your CIF. These basic checks will be run on all CIFs submitted for publication in IUCr journals (*Acta Crystallographica*, *Journal of Applied Crystallography*, *Journal of Synchrotron Radiation*); however, if you intend to submit to *Acta Crystallographica Section C* or *E* or *IUCrData*, you should make sure that full publication checks are run on the final version of your CIF prior to submission.

Publication of your CIF in other journals

Please refer to the *Notes for Authors* of the relevant journal for any special instructions relating to CIF submission.

PLATON version of 06/01/2024; check.def file version of 05/01/2024

Datablock bmk2355 - ellipsoid plot

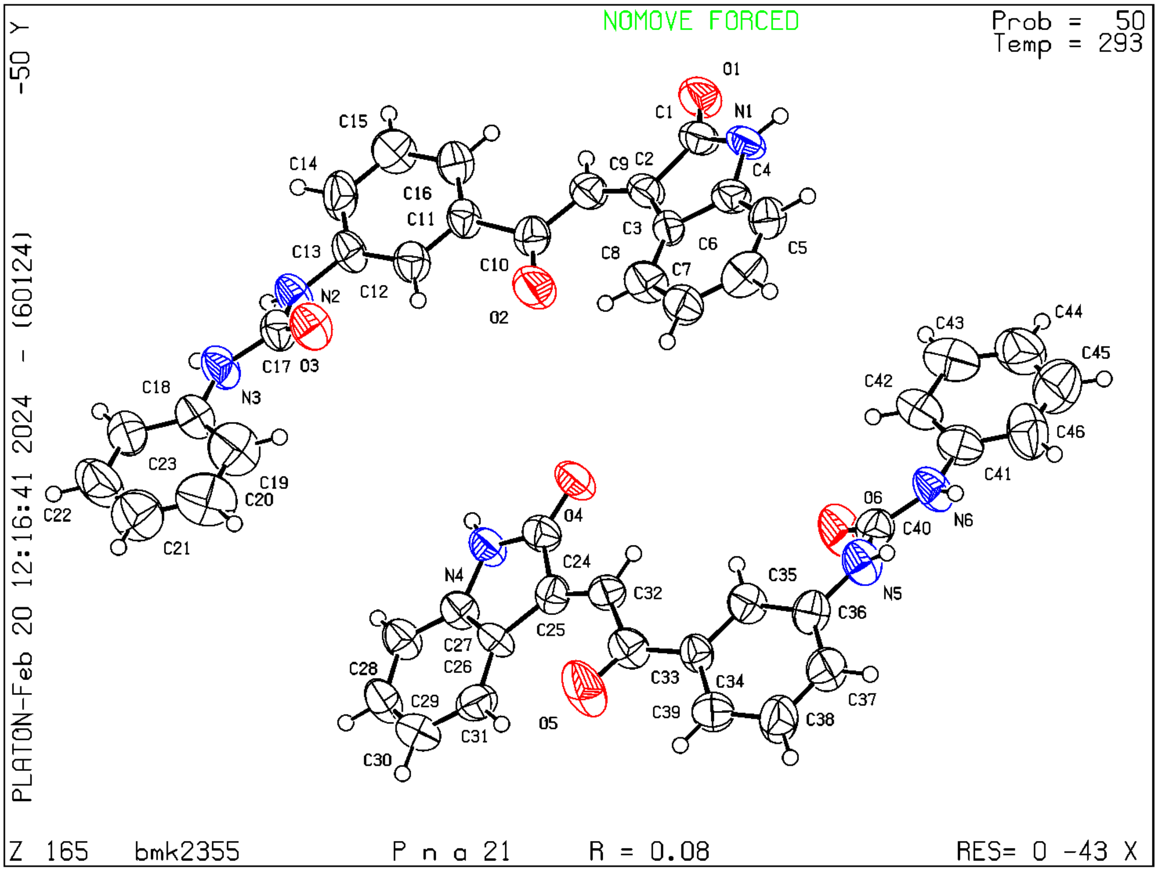

Supplement: Supplementary file 1 — Supplementary Information 1. [file 41598_2024_82005_MOESM1_ESM.pdf]
