# Supplementary material for: Design and synthesis of antiproliferative 2-oxoindolin-3-ylidenes incorporating urea function with potential VEGFR-2 inhibitory properties
Source: Sci Rep. 2025 Jan 3;15:618. doi: 10.1038/s41598-024-82005-6 (PMC11699130; doi:10.1038/s41598-024-82005-6)
Supplement: Supplementary file 2 — Supplementary Information 2. [file 41598_2024_82005_MOESM2_ESM.pdf]

Design and synthesis of antiproliferative 2-oxoindolin-3-ylidenes incorporating urea function with potential VEGFR-2 inhibitory properties

Dalia R. Aboshouk[1], M. Adel Youssef[2], Siva S. Panda[3] , Benson M. Kariuki[4], Mohamed S. Bekheit[1], Ahmed R. Hamed[5], Walid Fayad[6], Ahmed A. F. Soliman[6], Adel S. Girgis[1,]*

[1]Department of Pesticide Chemistry, National Research Centre, Dokki, Giza 12622, Egypt

[2]Department of Chemistry, Faculty of Science, Helwan University, Helwan, Egypt

[3]Department of Chemistry and Biochemistry, Augusta University, Augusta, GA,30912, USA

[4]School of Chemistry, Cardiff University, Main Building, Park Place, Cardiff, CF10 3AT, UK

[5]Chemistry of Medicinal Plants Department, National Research Centre, Dokki, Giza 12622, Egypt

[6]Drug Bioassay-Cell Culture Laboratory, Pharmacognosy Department, National Research Centre, Dokki, Giza, 12622. Egypt

* Corresponding author: girgisas10@yahoo.com, as.girgis@nrc.sci.eg

Supplementary material

Table titles

Table S1. Crystal data and structure refinement for compound **12a**.

Figure captions

Fig. S1. IR spectrum of compound **11a** (KBr pellet).

Fig. S2. ^{1}H-NMR spectrum of compound **11a** in DMSO-d_6.

Fig. S3. ^{13}C-NMR spectrum of compound **11a** in DMSO-d_6.

Fig. S4. IR spectrum of compound **11b** (KBr pellet).

Fig. S5. ^{1}H-NMR spectrum of compound **11b** in DMSO-d_6.

Fig. S6. ^{13}C-NMR spectrum of compound **11b** in DMSO-d_6.
Fig. S7. IR spectrum of compound **11c** (KBr pellet).
Fig. S8. ^{1}H-NMR spectrum of compound **11c** in DMSO-d_6.
Fig. S9. ^{13}C-NMR spectrum of compound **11c** in DMSO-d_6.
Fig. S10. IR spectrum of compound **11d** (KBr pellet).
Fig. S11. ^{1}H-NMR spectrum of compound **11d** in DMSO-d_6.
Fig. S12. ^{13}C-NMR spectrum of compound **11d** in DMSO-d_6.
Fig. S13. IR spectrum of compound **11e** (KBr pellet).
Fig. S14. ^{1}H-NMR spectrum of compound **11e** in DMSO-d_6.
Fig. S15. ^{13}C-NMR spectrum of compound **11e** in DMSO-d_6.
Fig. S16. IR spectrum of compound **11f** (KBr pellet).
Fig. S17. ^{1}H-NMR spectrum of compound **11f** in DMSO-d_6.
Fig. S18. ^{13}C-NMR spectrum of compound **11f** in DMSO-d_6.
Fig. S19. IR spectrum of compound **11g** (KBr pellet).
Fig. S20. ^{1}H-NMR spectrum of compound **11g** in DMSO-d_6.
Fig. S21. ^{13}C-NMR spectrum of compound **11g** in DMSO-d_6.
Fig. S22. IR spectrum of compound **11h** (KBr pellet).
Fig. S23. ^{1}H-NMR spectrum of compound **11h** in DMSO-d_6.
Fig. S24. ^{13}C-NMR spectrum of compound **11h** in DMSO-d_6.
Fig. S25. IR spectrum of compound **11i** (KBr pellet).
Fig. S26. ^{1}H-NMR spectrum of compound **11i** in DMSO-d_6.
Fig. S27. ^{13}C-NMR spectrum of compound **11i** in DMSO-d_6.
Fig. S28. IR spectrum of compound **11j** (KBr pellet).
Fig. S29. ^{1}H-NMR spectrum of compound **11j** in DMSO-d_6.
Fig. S30. ^{13}C-NMR spectrum of compound **11j** in DMSO-d_6.
Fig. S31. IR spectrum of compound **12a** (KBr pellet).
Fig. S32. ^{1}H-NMR spectrum of compound **12a** in DMSO-d_6.
Fig. S33. ^{13}C-NMR spectrum of compound **12a** in DMSO-d_6.
Fig. S34. IR spectrum of compound **12b** (KBr pellet).
Fig. S35. ^{1}H-NMR spectrum of compound **12b** in DMSO-d_6.
Fig. S36. ^{13}C-NMR spectrum of compound **12b** in DMSO-d_6.

Fig. S37. IR spectrum of compound **12c** (KBr pellet).

Fig. S38. ^{1}H-NMR spectrum of compound **12c** in DMSO-d_6.

Fig. S39. ^{13}C-NMR spectrum of compound **12c** in DMSO-d_6.

Fig. S40. IR spectrum of compound **12d** (KBr pellet).

Fig. S41. ^{1}H-NMR spectrum of compound **12d** in DMSO-d_6.

Fig. S42. ^{13}C-NMR spectrum of compound **12d** in DMSO-d_6.

Fig. S43. IR spectrum of compound **12e** (KBr pellet).

Fig. S44. ^{1}H-NMR spectrum of compound **12e** in DMSO-d_6.

Fig. S45. ^{13}C-NMR spectrum of compound **12e** in DMSO-d_6.

Fig. S46. IR spectrum of compound **12f** (KBr pellet).

Fig. S47. ^{1}H-NMR spectrum of compound **12f** in DMSO-d_6 [singals at δ = 2.74, 2.89, 7.96 are for DMF, solvent of crystallization, Organometallics 29, 2176–2179 (2010)].

Fig. S48. ^{13}C-NMR spectrum of compound **12f** in DMSO-d_6 [singals at δ = 30.7, 35.7, 162.2 are for DMF, solvent of crystallization, Organometallics 29, 2176–2179 (2010)].

Fig. S49. IR spectrum of compound **12g** (KBr pellet).

Fig. S50. ^{1}H-NMR spectrum of compound **12g** in DMSO-d_6.

Fig. S51. ^{13}C-NMR spectrum of compound **12g** in DMSO-d_6.

Fig. S52. IR spectrum of compound **12h** (KBr pellet).

Fig. S53. ^{1}H-NMR spectrum of compound **12h** in DMSO-d_6.

Fig. S54. ^{13}C-NMR spectrum of compound **12h** in DMSO-d_6.

Fig. S55. IR spectrum of compound **12i** (KBr pellet).

Fig. S56. ^{1}H-NMR spectrum of compound **12i** in DMSO-d_6.

Fig. S57. ^{13}C-NMR spectrum of compound **12i** in DMSO-d_6.

Fig. S58. IR spectrum of compound **12j** (KBr pellet).

Fig. S59. ^{1}H-NMR spectrum of compound **12j** in DMSO-d_6.

Fig. S60. ^{13}C-NMR spectrum of compound **12j** in DMSO-d_6.

Fig. S61. Dose response curves of **12a–j** and sunitinib against HCT116 (colon) cancer cell line.

Fig. S62. Dose response curves of **12a–j** and sunitinib against MCF7 (breast) cancer cell line.

Fig. S63. Dose response curves of **12a–j** and sunitinib against PaCa2 (pancreatic) cancer cell line.

Fig. S64. Dose response curves of **12a–j** against PaCa2 (pancreatic) cancer cell line.

Fig. S65a. VEGFR-2 inhibitory properties of **12a–j** and sunitinib utilizing Western Blotting technique (uncropped).

Fig. S65b. VEGFR-2 inhibitory properties of **12a–j** and sunitinib utilizing Western Blotting technique (cropped).

Fig. S66. Docking poses of the **12a–j** and sorafenib (co-crystallized ligand) in PDB ID: 3WZE.

Fig. S67. Docking poses of the **12a–j** and sunitinib (co-crystallized ligand) in PDB ID: 3AGD.

Single crystal X-ray studies

Single-crystal XRD data for compound **12a** were collected at room temperature on an Agilent SuperNova Dual Atlas diffractometer with a mirror monochromator using Mo radiation. The crystal structure was solved using SHELXT[1] and refined using SHELXL.[2] Non-hydrogen atoms were refined with anisotropic displacement parameters. Hydrogen atoms were inserted in idealized positions, and a riding model was used with Uiso set at 1.2 or 1.5 times the value of Ueq for the atom to which they are bonded. Table S1 shows crystal and structure refinement data. The crystal structure has been deposited in the Cambridge Structure Database with reference CCDC 2333946.

Table S1. Crystal data and structure refinement for compound **12a**.

Empirical formula	$C_{23}H_{17}N_3O_3$
Formula weight	383.39
Temperature	293(2) K
Wavelength	0.71073 Å
Crystal system	Orthorhombic
Space group	$Pna2_1$
a	9.2245(9) Å
b	12.9911(11) Å
c	30.476(3) Å
α	90°
β	90°
γ	90°
Volume	3652.2(6) Å^3
Z	8
Density (calculated)	1.395 Mg/m^3
Absorption coefficient	0.094 mm^{-1}
F(000)	1600

Crystal size	0.240 x 0.060 x 0.030 mm^3
Theta range for data collection	3.370 to 29.885°.
Index ranges	-12<=h<=8, -16<=k<=17, -26<=l<=42
Reflections collected	16630
Independent reflections	7604 [R(int) = 0.0970]
Completeness to theta = 25.242°	99.8 %
Refinement method	Full-matrix least-squares on F^2
Data / restraints / parameters	7604 / 13 / 524
Goodness-of-fit on F^2	0.971
Final R indices [I>2sigma(I)]	R1 = 0.0777, wR2 = 0.1556
R indices (all data)	R1 = 0.2274, wR2 = 0.2238
Absolute structure parameter	-2.2(10)
Extinction coefficient	0.0010(4)
Largest diff. peak and hole	0.276 and -0.225 e.$Å^{-3}$

Biological studies

All the biological studies conducted obey the standards and approved by the Research Ethics Committee, National Research Centre, Egypt (associated with project ID: 13060103).

Antiproliferation properties

The synthesized compounds **12a–j** were screened for their antiproliferation properties against HCT116 (colon), MCF7 (breast) and PaCa2 (pancreatic) cancer cell lines by the standard mitochondrial dependent reduction of yellow MTT [3-(4,5-dimethylthiazol-2-yl)-2,5-diphenyl-tetrazolium bromide] to purple formazan technique.[3,4] Sunitinib was considered as standard reference/drug. Cells were suspended in DMEM medium for MCF7, PaCa-2 and McCoy's 5A for HCT116 in addition to 1% antibiotic–antimycotic mixture (10000 μg ml^{-1} potassium penicillin, 10000 μg ml^{-1} streptomycin sulfate and 25 μg ml^{-1} amphotericin B), 10% fetal bovine serum and 1% L-glutamine at 37 °C, under 5% CO_2 and 95% humidity. Cells were seeded at concentration of 30000 cells per well in fresh complete growth medium in 96-well tissue culture microtiter plates for 24 h. Media

was aspirated, fresh complete medium was added and cells were incubated with different concentrations of the tested compound to give a final concentration of [50, 25, 12.5 and 6.25 μM “in addition to 3.125, 1.56 and 0.78 μM in case of high potent analogs”). 0.5% DMSO was used as a negative control. Triplicate wells were prepared for each individual dose. After 72 h of incubation, medium was aspirated, 40 μl MTT salt (2.5 mg ml^{-1}) was added to each well and incubated for further 4 h at 37 °C. To stop the reaction and dissolve the formed crystals, 150 μl of 10% sodium dodecyl sulfate (SDS) in deionized water was added to each well and incubated overnight at 37 °C. The absorbance was then measured at 570 nm and a reference wavelength of 595 nm.

Data were collected as mean values for experiments performed in triplicates for each individual dose which had been measured by MTT assay. Control experiments did not exhibit significant change compared to the DMSO vehicle. The cell surviving fraction was calculated according to the following equation.

$$Surviving\ fraction = \frac{Optical density (O.D.) of treated cells}{O.D. of control cells}$$

The agents synthesized were also tested against RPE1 (normal human immortalized retinal pigment epithelial cell line) cell (in DMEM-F12 medium) to determine the toxicity/selectivity towards normal cells relative to the cancer cell lines utilized.

The IC_{50} (concentration required to produce 50% inhibition of cell growth compared to the control experiment) was determined using Graph-Pad PRISM version-5 software. Statistical calculations for determination of the mean and standard division (SD) values were determined by SPSS 16 software. The observed anti-proliferative properties are presented in Table 1 (Supplementary Figs. S61–S64).

VEGFR-2 inhibitory properties

The VEGFR-2 properties of the synthesized agents (**12a–j**) and sunitinib (reference standard/drug) was determined at 10 μM by the western blotting technique obeying the manufacturer’s instruction (Santa Cruz Biotechnology, Inc.).[5]

Protein extraction procedure

The ReadyPrepTM protein extraction kit (total protein) provided by Bio-Rad Inc. (Catalog #163-2086) employed by the manufacturer, was added to each sample of the lysed cells. Bradford Protein Assay Kit (SK3041) for quantitative protein analysis was provided by Bio-basic Inc. (Markham Ontario L3R 8T4 Canada). A Bradford assay was performed according to manufacturer's instructions to determine protein concentration in each sample. 20 µg protein concentration of each sample was then loaded with an equal volume of 2x Laemmli sample buffer containing 4% SDS, 10% 2-mercaptoehtanol, 20% glycerol, 0.004% bromophenol blue and 0.125 M Tris HCl. The pH was checked and brought to 6.8. Each previous mixture was boiled at 95°C for 5 min to ensure denaturation of protein before loading on polyacrylamide gel electrophoresis.

Protein separation by electrophoresis

Samples were separated on a polyacrylamide gel; the procedure was abbreviated as SDS-PAGE form Sodium Dodecyl Sulfate PolyAcrylamide Gel Electrophoresis, which is a standard technique for separating proteins according to their molecular weight. Polyacrylamide gels were performed using TGX Stain-Free™ FastCast™ Acrylamide Kit (SDS-PAGE), which was provided by Bio-Rad Laboratories inc Cat # 161-0181. The SDS-PAGE TGX Stain-Free FastCast was prepared according to manufacturer's instructions.

Protein blotting (transfer of proteins from the gel to the membrane)

The gel was assembled in transfer sandwich from below to above (filter paper, PVDF membrane, gel and filter paper). The sandwich was placed in the transfer tank with 1x transfer buffer, which is composed of 25 mM Tris and 190 mM glycine and 20% methanol. Then, the blot was run for 7 min. at 25 V to allow protein bands transfer from gel to membrane using BioRad Trans-Blot Turbo.

Blocking the membrane

The membrane was blocked in tris-buffered saline with Tween 20 (TBST) buffer and 3% bovine serum albumin (BSA) at room temperature for 1 hr. The components of blocking

buffer were as follow; 20 mM Tris pH 7.5, 150 mM NaCl, 0.1% Tween 20 and 3% bovine serum albumin (BSA).

Incubation with the primary antibody

Primary antibody of VEGFR2 was purchased. Primary antibody was diluted in TBST according to manufactured instructions. Incubation was done overnight in each primary antibody solution, against the blotted target protein, at 4 °C. The blot was rinsed 3–5 times for 5 min with TBST. Incubation was done in the HRP-conjugated secondary antibody (Goat anti-rabbit IgG- HRP-1mg Goat mab -Novus Biologicals) solution against the blotted target protein for 1 h at room temperature. The blot was rinsed 3–5 times for 5 min. with TBST.

Imaging and data analysis quantitation:

The chemiluminescent substrate (ClarityTM Western ECL substrate Bio-Rad cat#170-5060) was applied to the blot according to the manufacturer's recommendation. Briefly, equal volumes were added from solution A (Clarity western luminal/enhancer solution) and solution B (peroxidase solution). The chemiluminescent signals were captured using a CCD camera-based imager. Image analysis software was used to read the band intensity of the target proteins against control sample beta actin (housekeeping protein) by protein normalization on the ChemiDoc MP imager.

Molecular modeling

The synthesized agents **12a–j** were considered for molecular modeling (docking) studies utilizing PDB ID: 3WZE and 3AGD[6,7] by Discovery Studio 2.5 software (RMS gradient: 0.0804, 0.0872, respectively). The standard CDOCKER technique was adopted after protein and ligand optimization (force field: CHARMm, Partial charge: MMFF94, radius of the active site employed: 8.0368 Å).[8]

References

1. Sheldrick, G. M. SHELXT - Integrated space-group and crystal-structure determination. *Acta Crystallogr., Sect. A* **71**(Pt 1), 3–8 (2015).

DOI:10.1107/S2053273314026370.

2. Sheldrick, G. M. Crystal structure refinement with SHELXL. *Acta Crystallogr., Sect. C* **71**, 3–8 (2015). DOI:10.1107/S2053229614024218.
3. Youssef, M. A., Panda, S. S., Aboshouk, D. R., Said, M. F., El Taweel, A., GabAllah, M., Fayad, W., Soliman, A. A. F., Mostafa, A., Fawzy, N. G., Girgis, A. S. Novel curcumin mimics: Design, synthesis, biological properties and computational studies of piperidone-piperazine conjugates. *ChemistrySelect* **7**, e202201406 (2022). DOI:10.1002/slct.202201406.
4. Fawzy, N. G., Panda, S. S., Fayad, W., Shalaby, E. M., Srour, A. M., Girgis, A. S. Synthesis, human topoisomerase IIα inhibitory properties and molecular modeling studies of anti-proliferative curcumin mimics. *RSC Adv.* **9**, 33761–33774 (2019). DOI:10.1039/c9ra05661k.
5. VEGFR2 (A-3): sc-6251, SANTA CRUZ BIOTECHNOLOGY, INC. (www.scbt.com).
6. https://www.rcsb.org/structure/3WZE (accessed on Jul. 28, 2024)
7. https://www.rcsb.org/structure/3AGD (accessed on Jul. 28, 2024)
8. Bekheit, M. S., Panda, S. S., Kariuki, B. M., Mahmoud, S. H., Mostafa, A., Girgis, A. S. Spiroindole-containing compounds bearing phosphonate group of potential M^{pro}-SARS-CoV-2 inhibitory properties. *Eur. J. Med. Chem.* **258**, 115563 (2023). DOI:10.1016/j.ejmech.2023.115563.

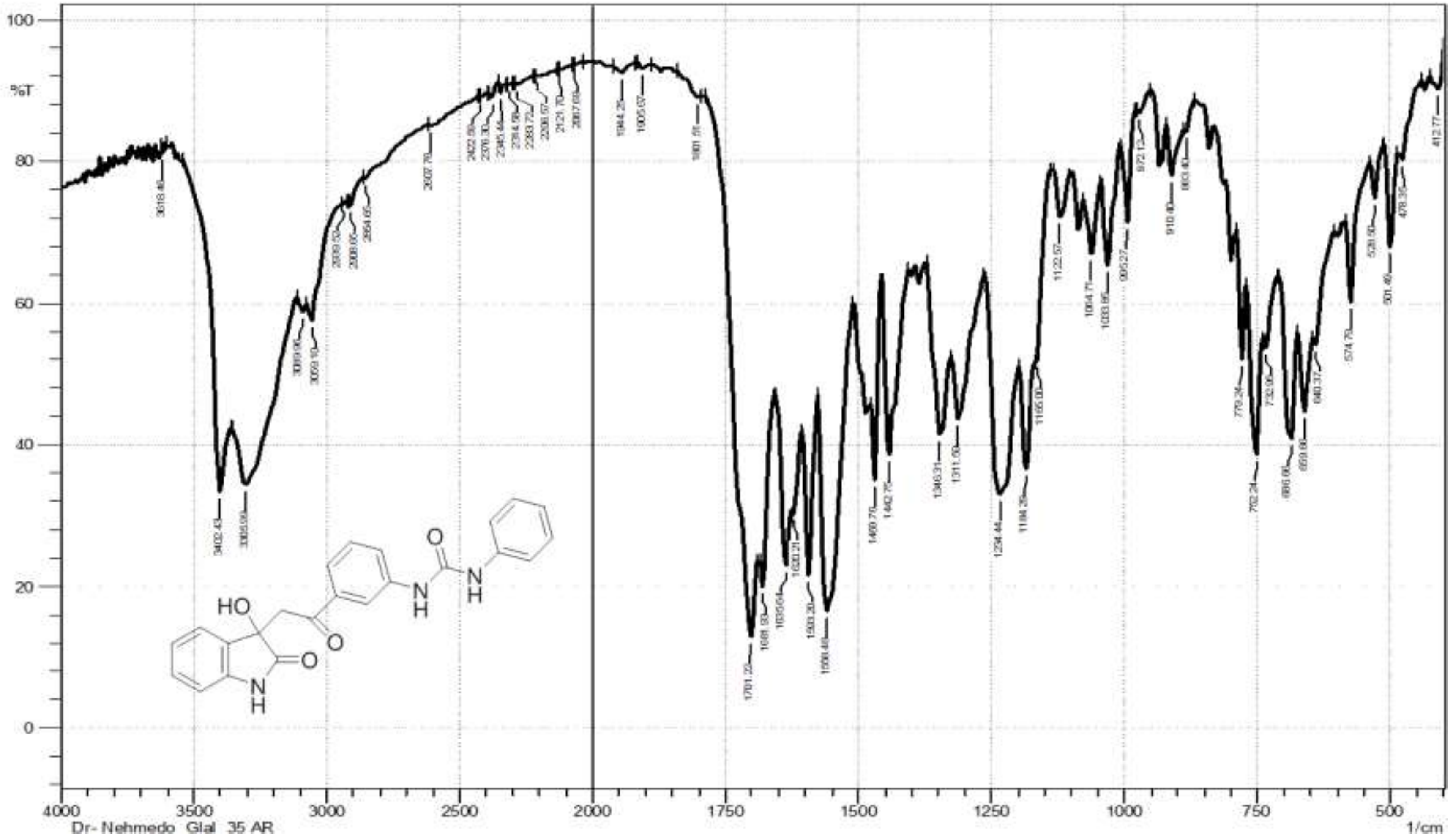

Fig. S1. IR spectrum of compound **11a** (KBr pellet).

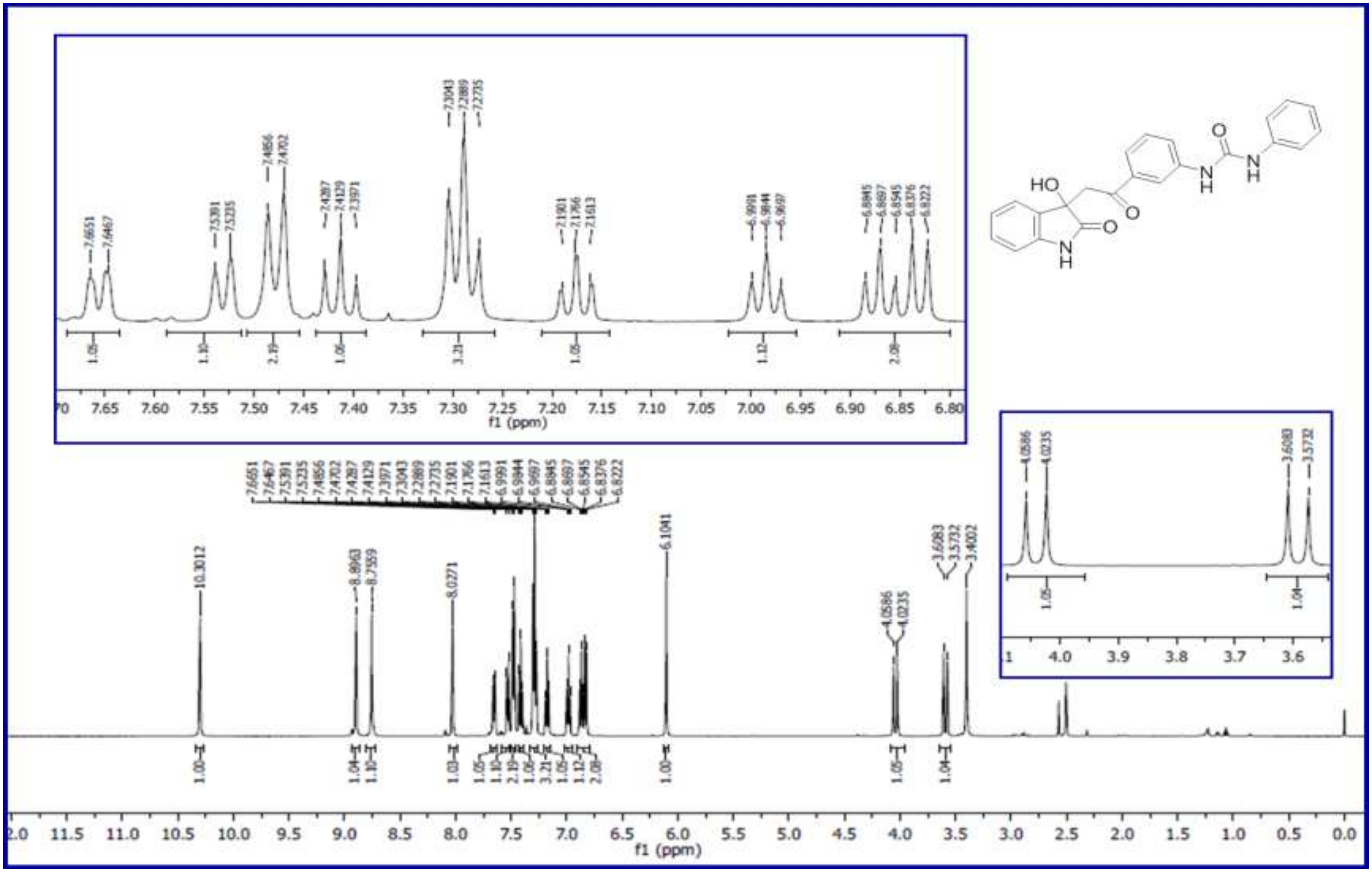

Fig. S2. ^{1}H-NMR spectrum of compound **11a** in DMSO-d_6.

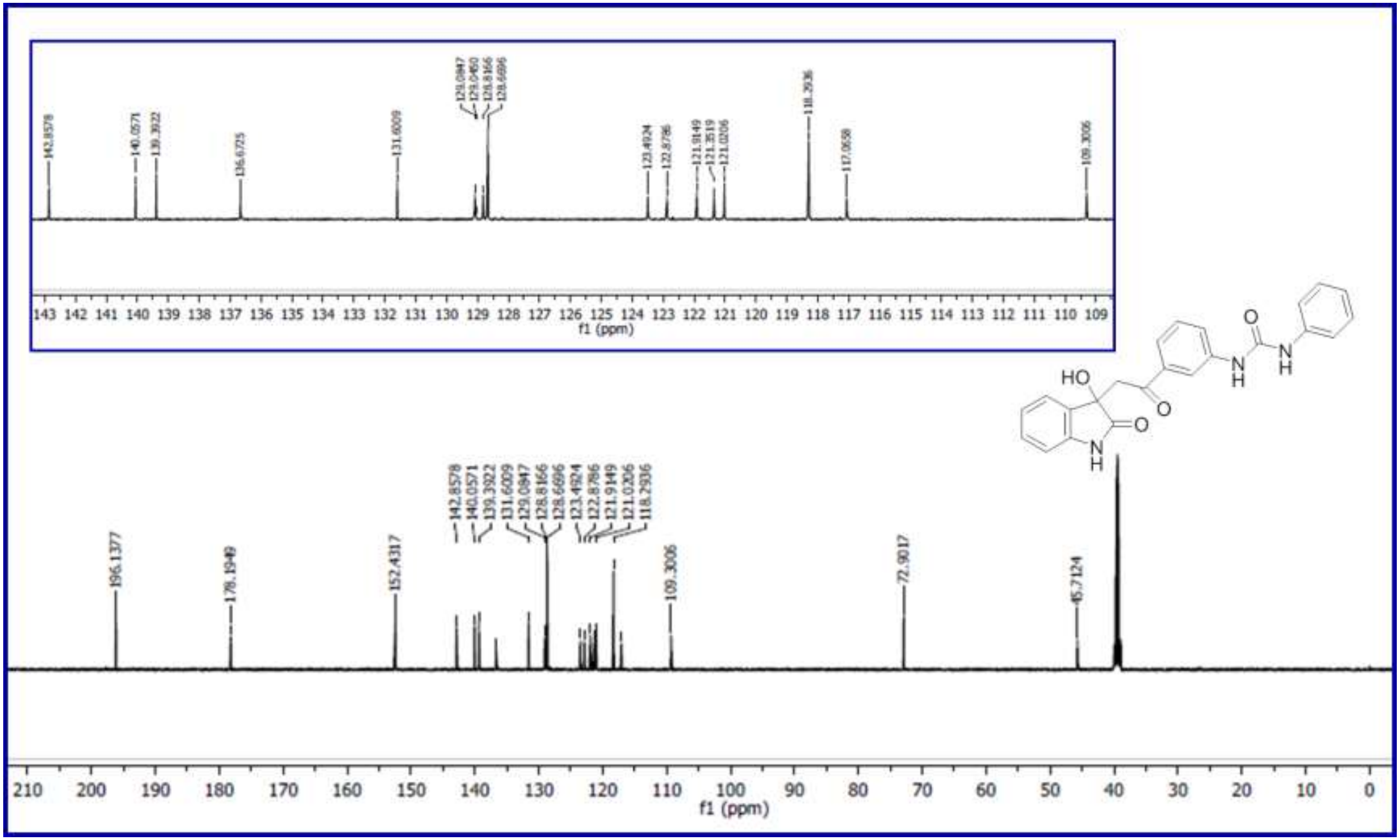

Fig. S3. ^{13}C-NMR spectrum of compound **11a** in DMSO-d_6.

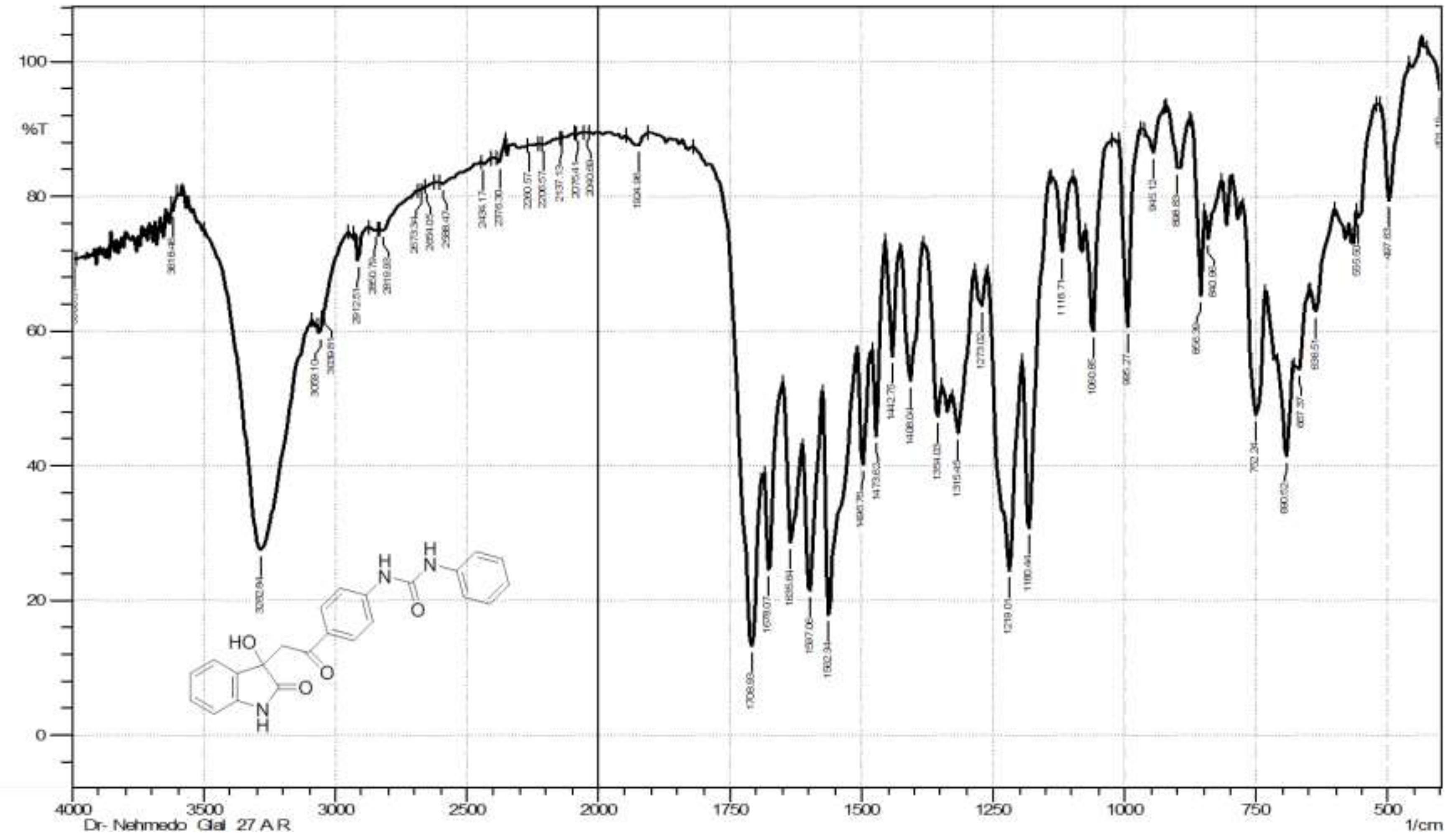

Fig. S4. IR spectrum of compound **11b** (KBr pellet).

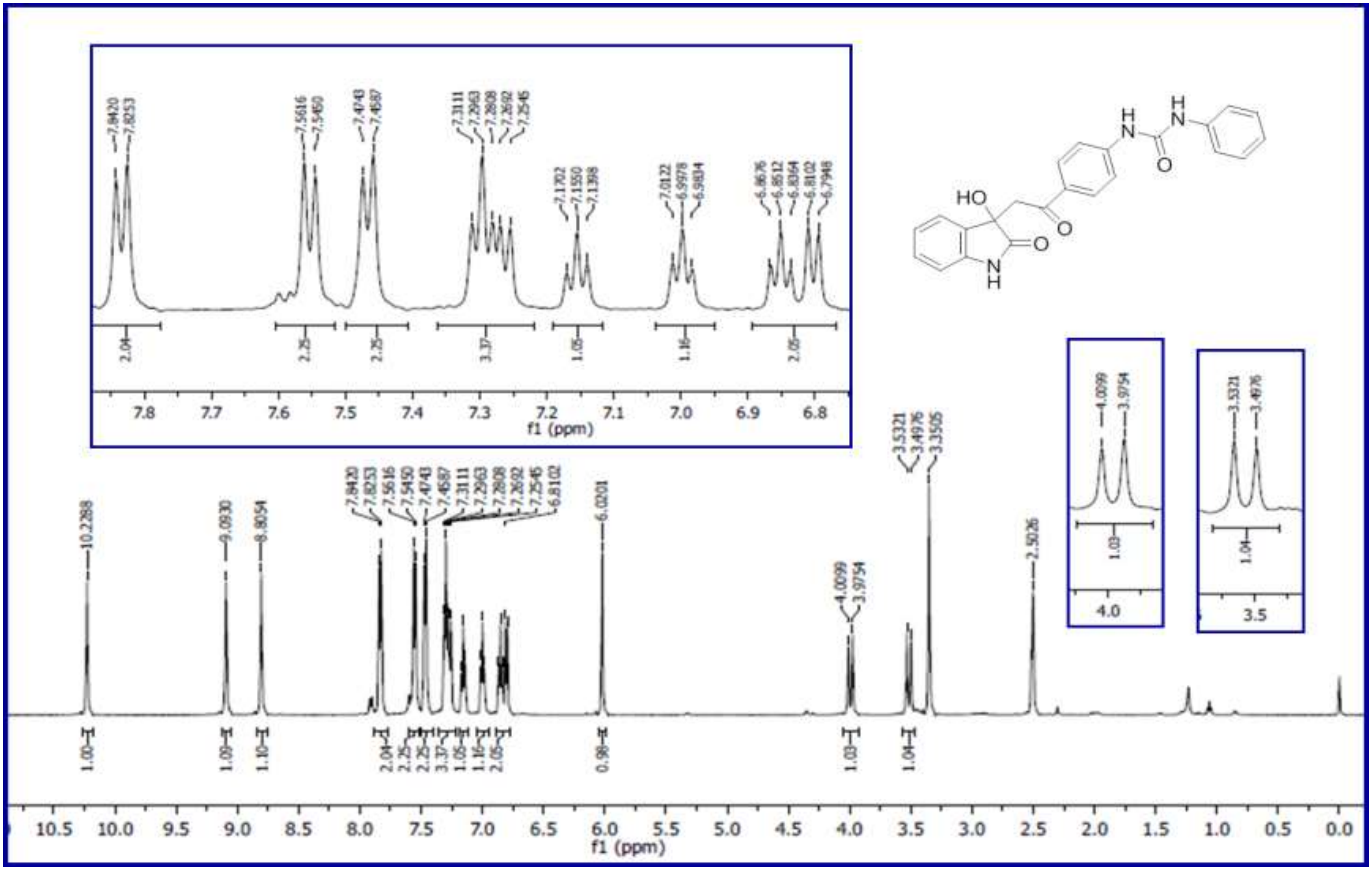

Fig. S5. ^{1}H-NMR spectrum of compound **11b** in DMSO-d_6.

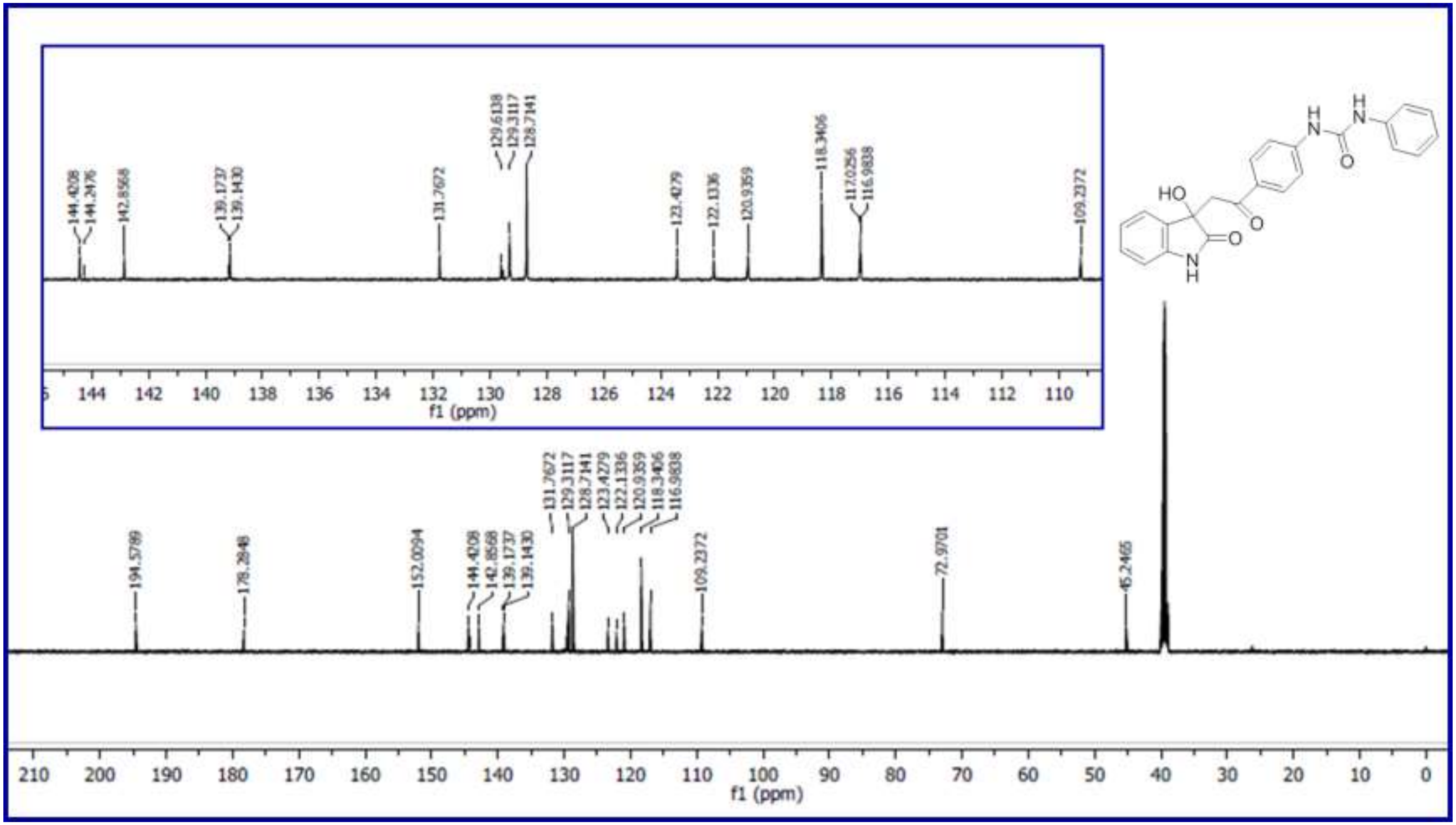

Fig. S6. ^{13}C-NMR spectrum of compound **11b** in DMSO-d_6.

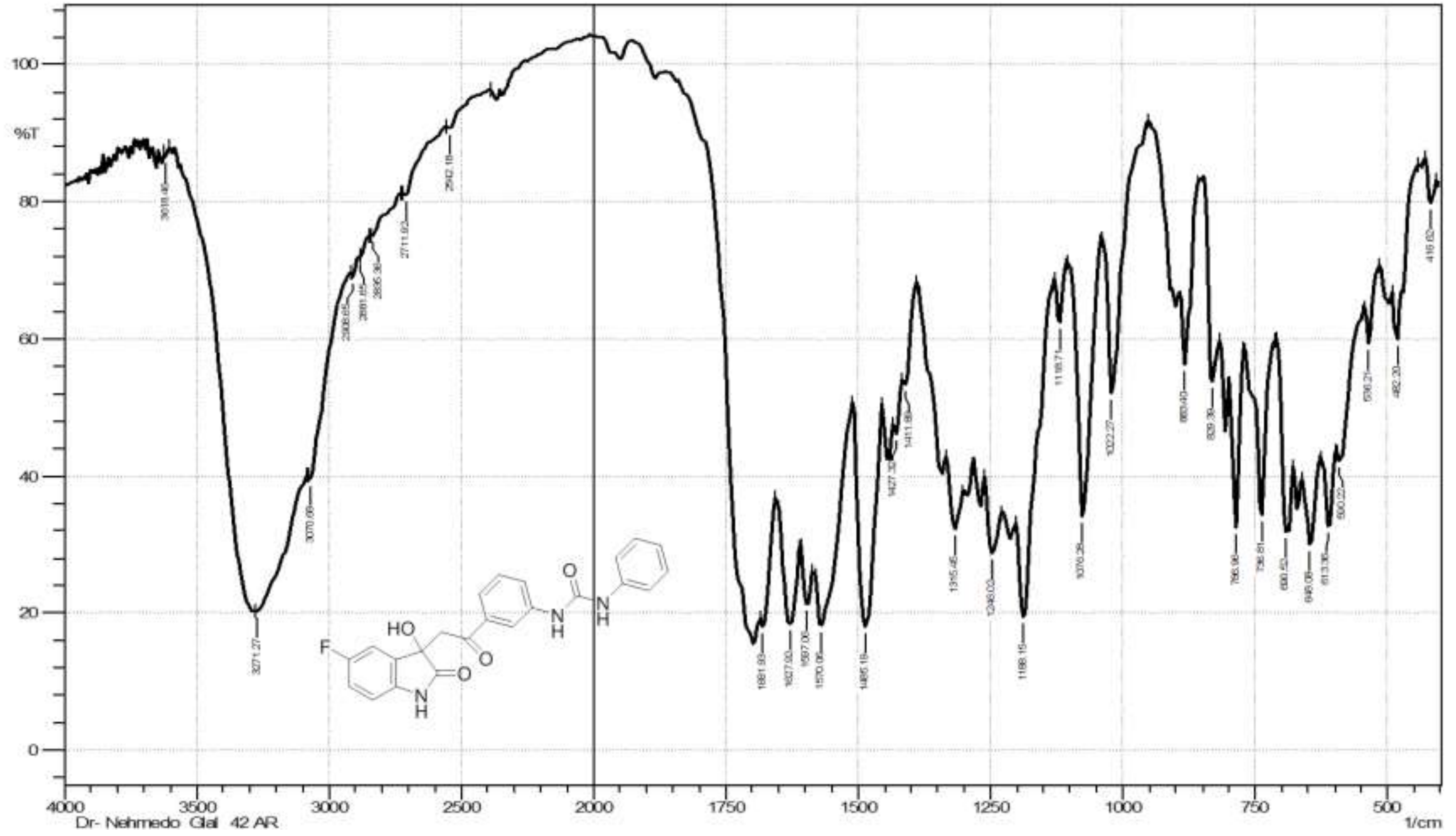

Fig. S7. IR spectrum of compound **11c** (KBr pellet).

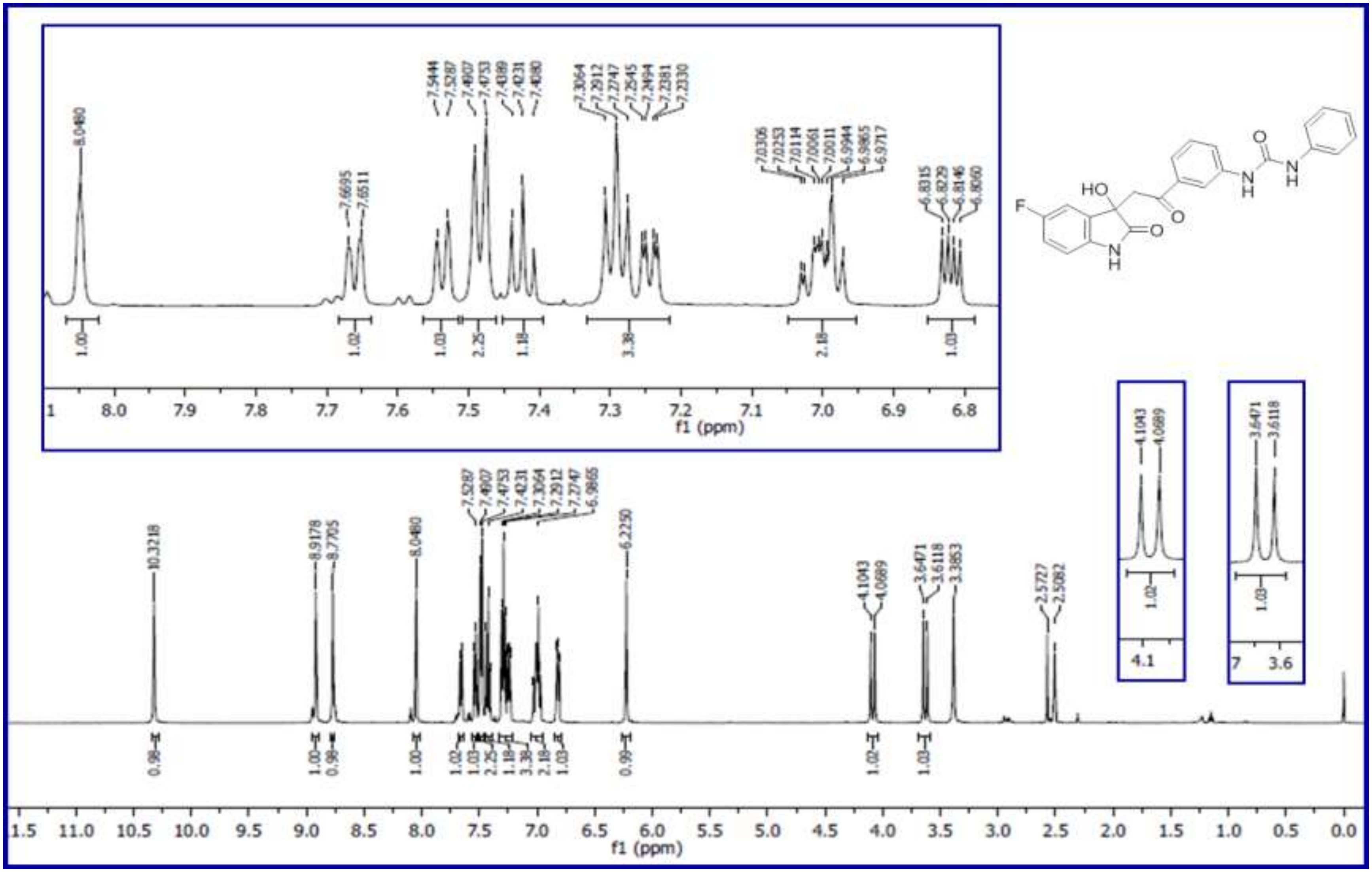

Fig. S8. ^{1}H-NMR spectrum of compound **11c** in DMSO-d_6.

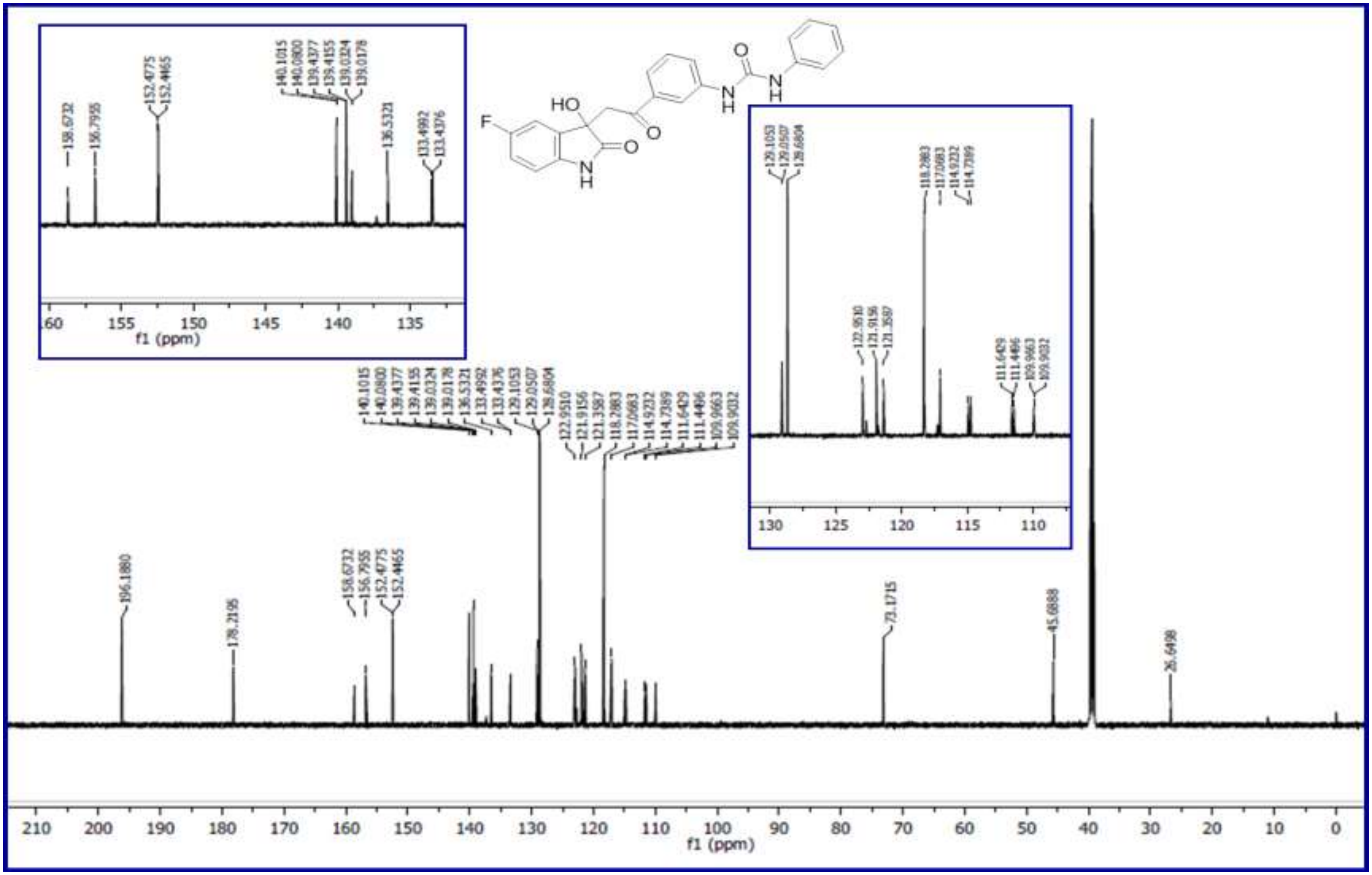

Fig. S9. ^{13}C-NMR spectrum of compound **11c** in DMSO-d_6.

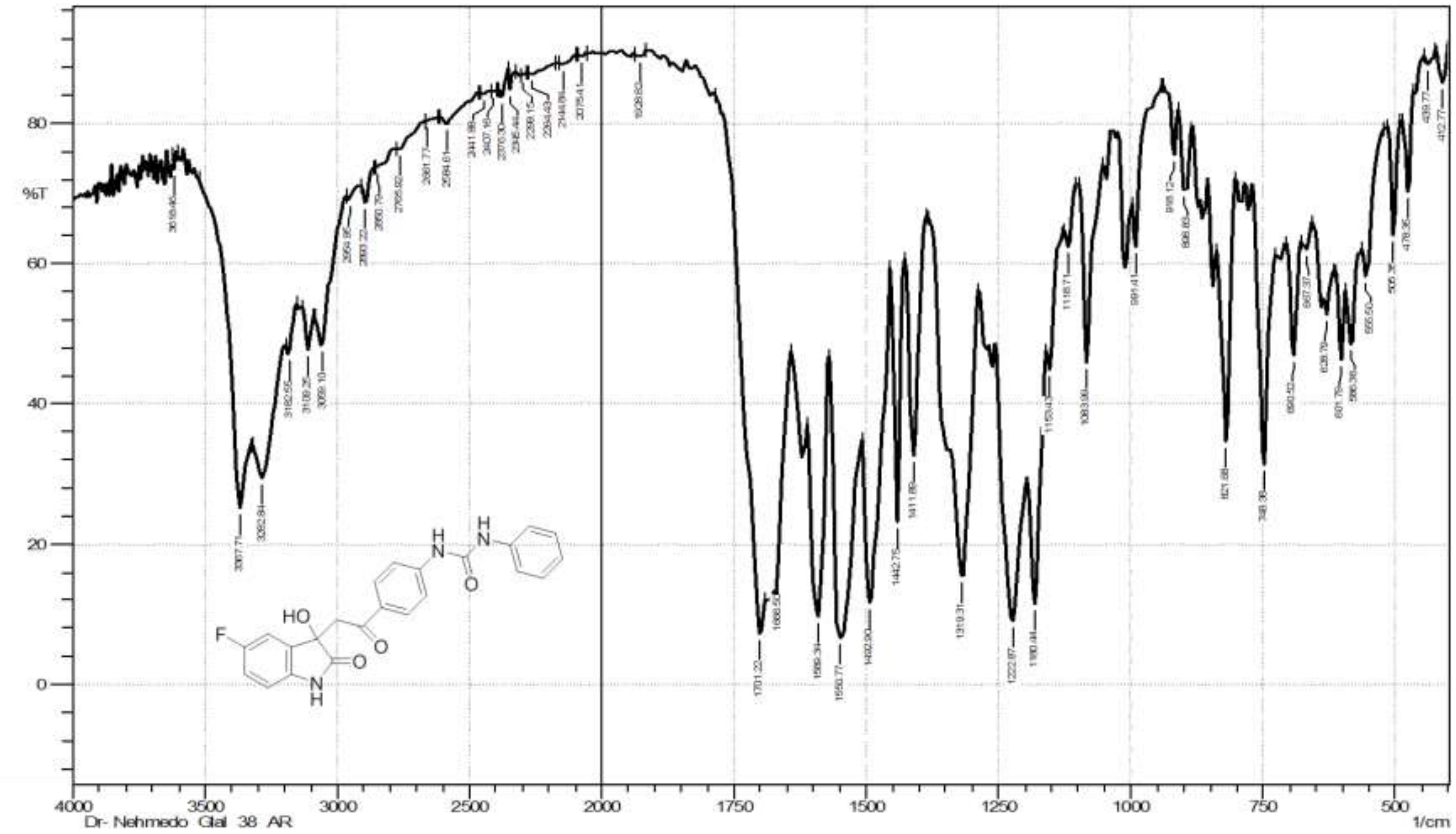

Fig. S10. IR spectrum of compound **11d** (KBr pellet).

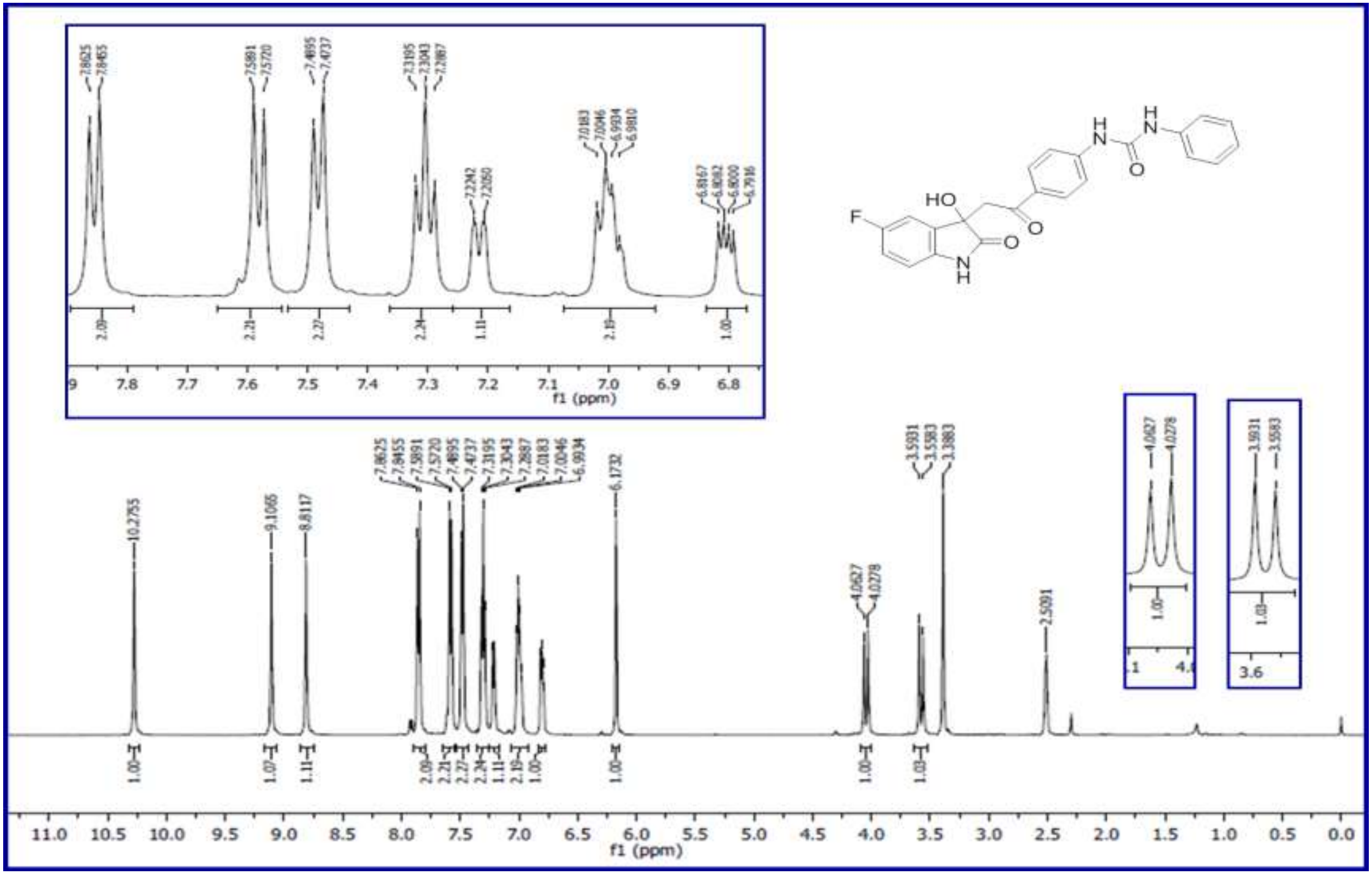

Fig. S11. ^{1}H-NMR spectrum of compound **11d** in DMSO-d_6.

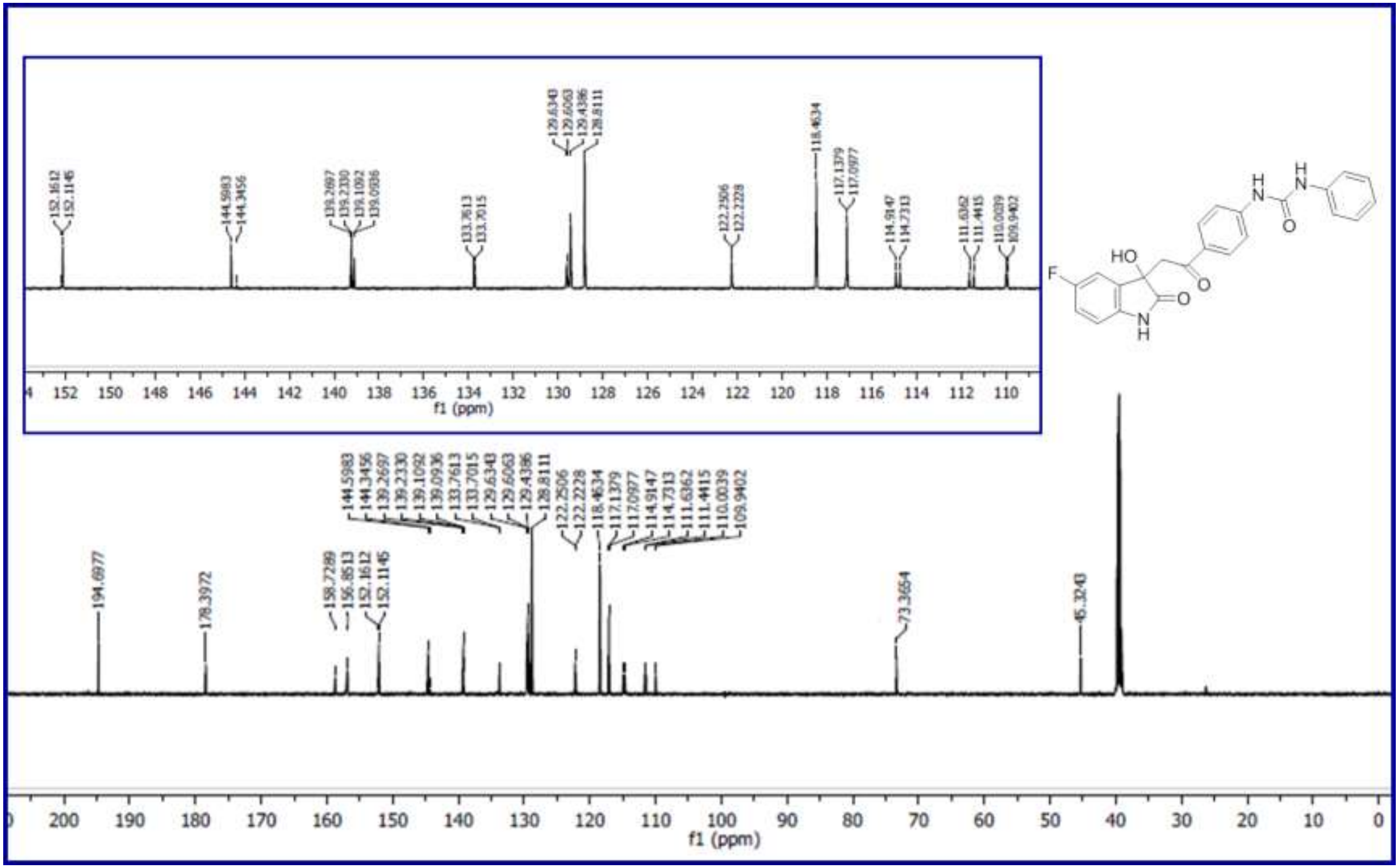

Fig. S12. ^{13}C-NMR spectrum of compound **11d** in DMSO-d_6.

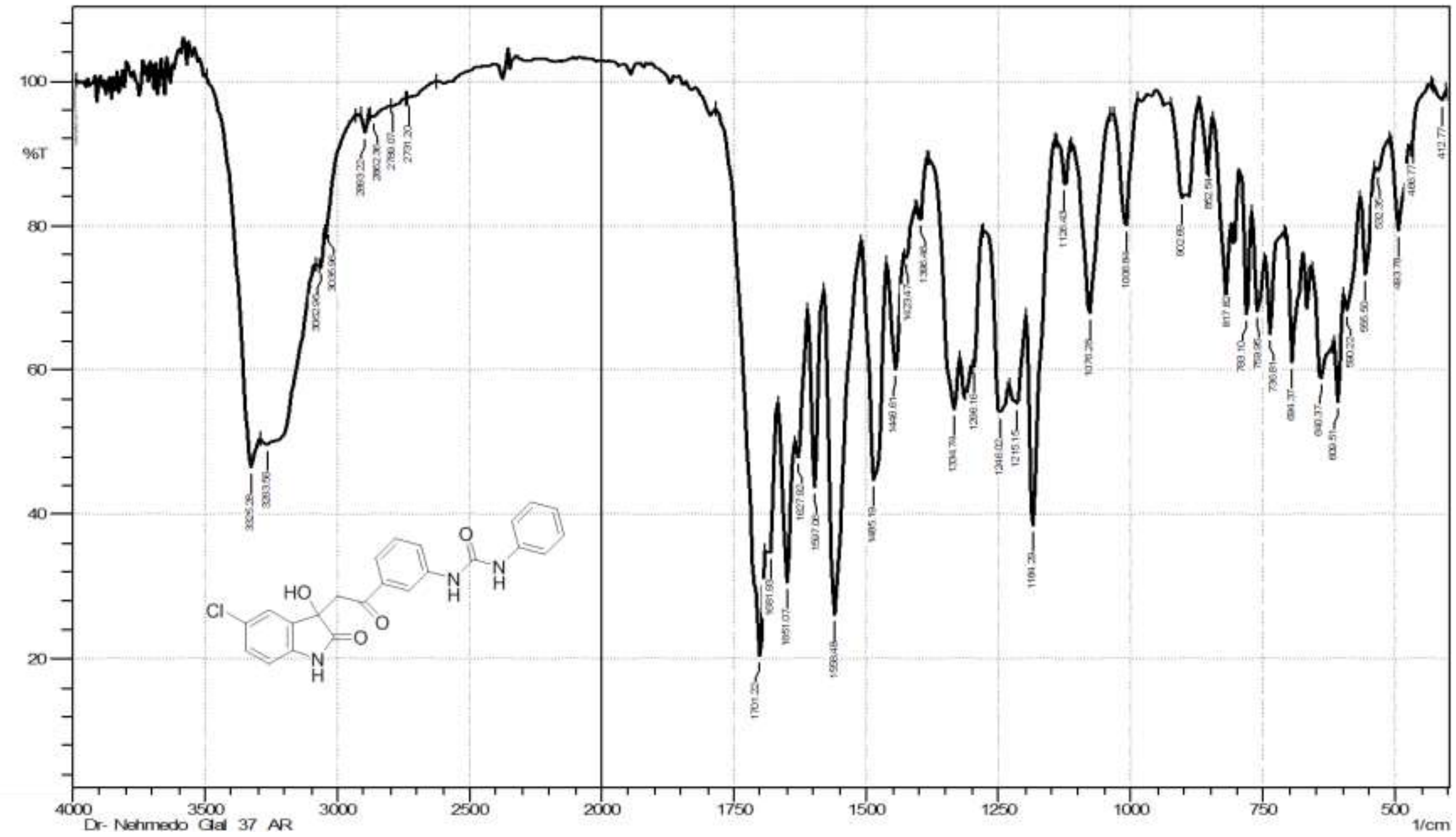

Fig. S13. IR spectrum of compound **11e** (KBr pellet).

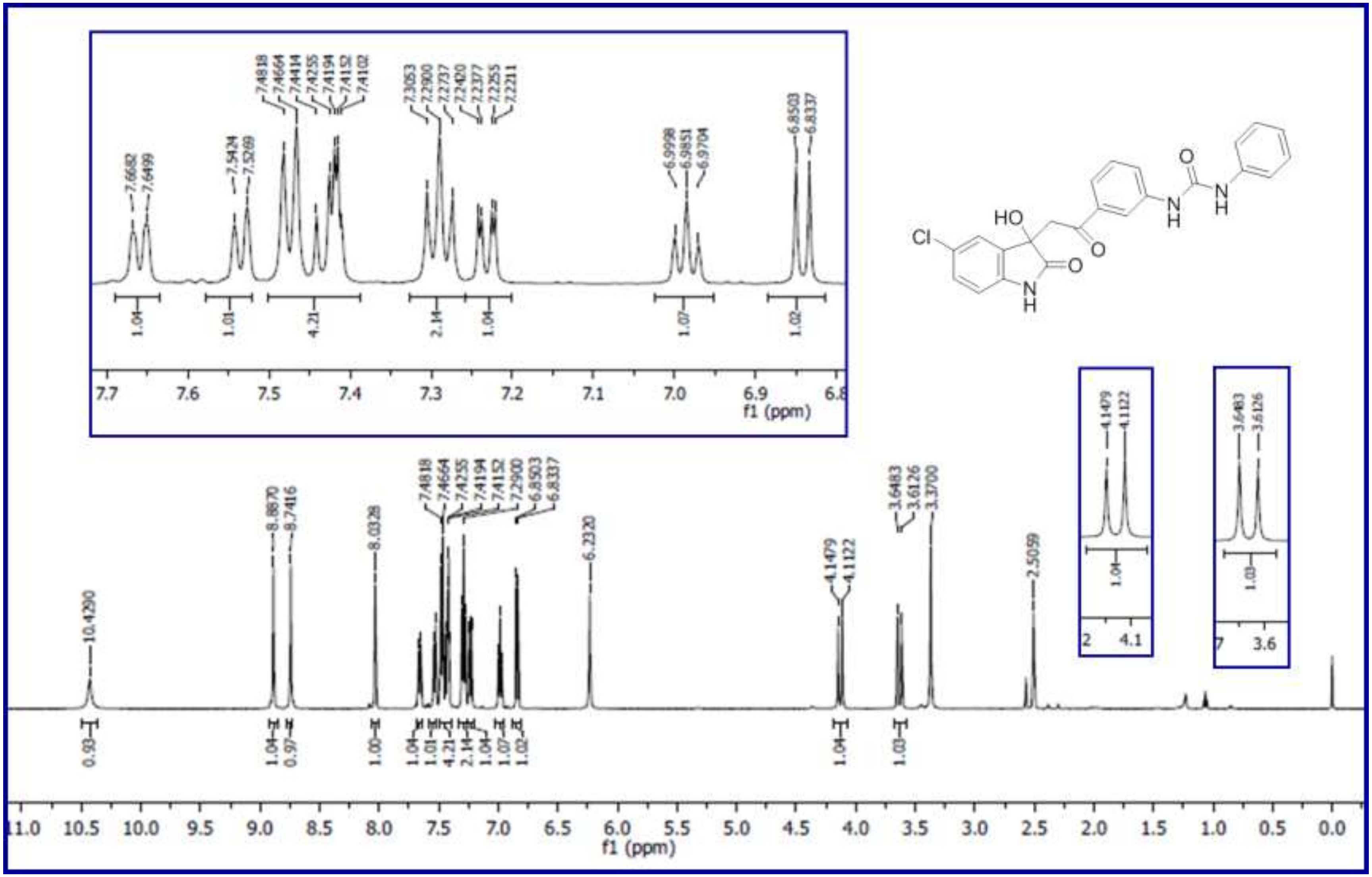

Fig. S14. ^{1}H-NMR spectrum of compound **11e** in DMSO-d_6.

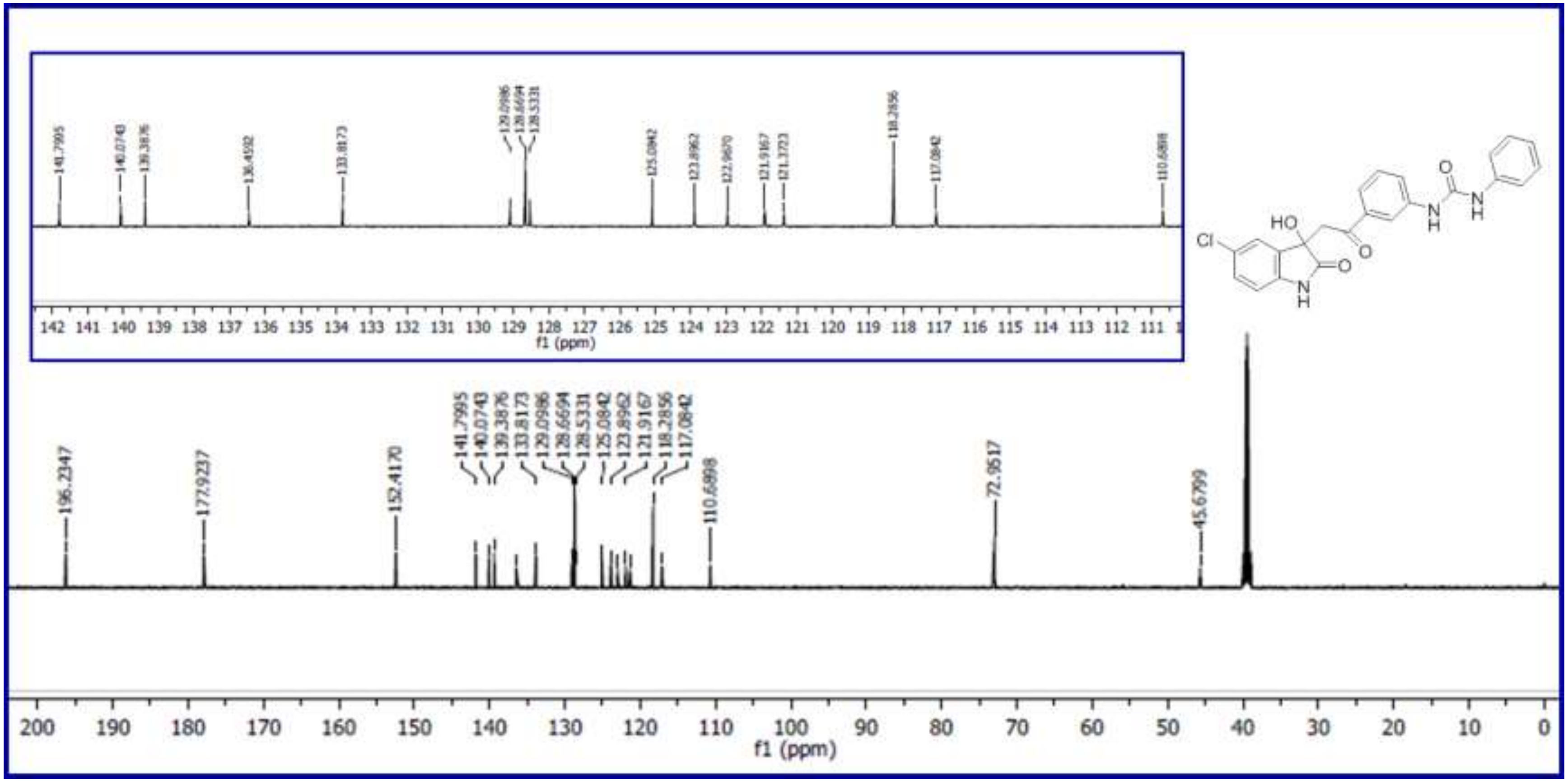

Fig. S15. ^{13}C-NMR spectrum of compound **11e** in DMSO-d_6.

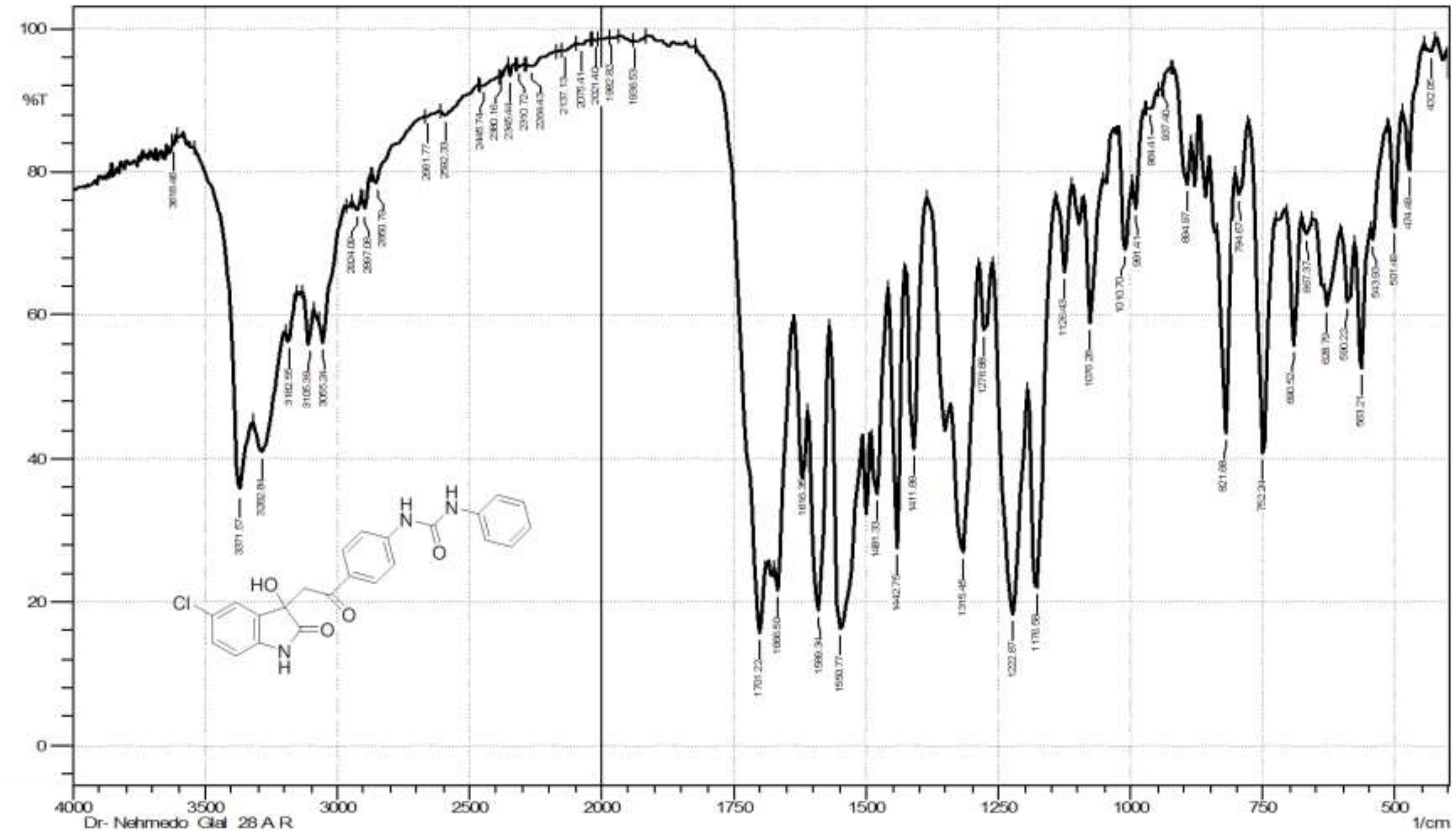

Fig. S16. IR spectrum of compound **11f** (KBr pellet).

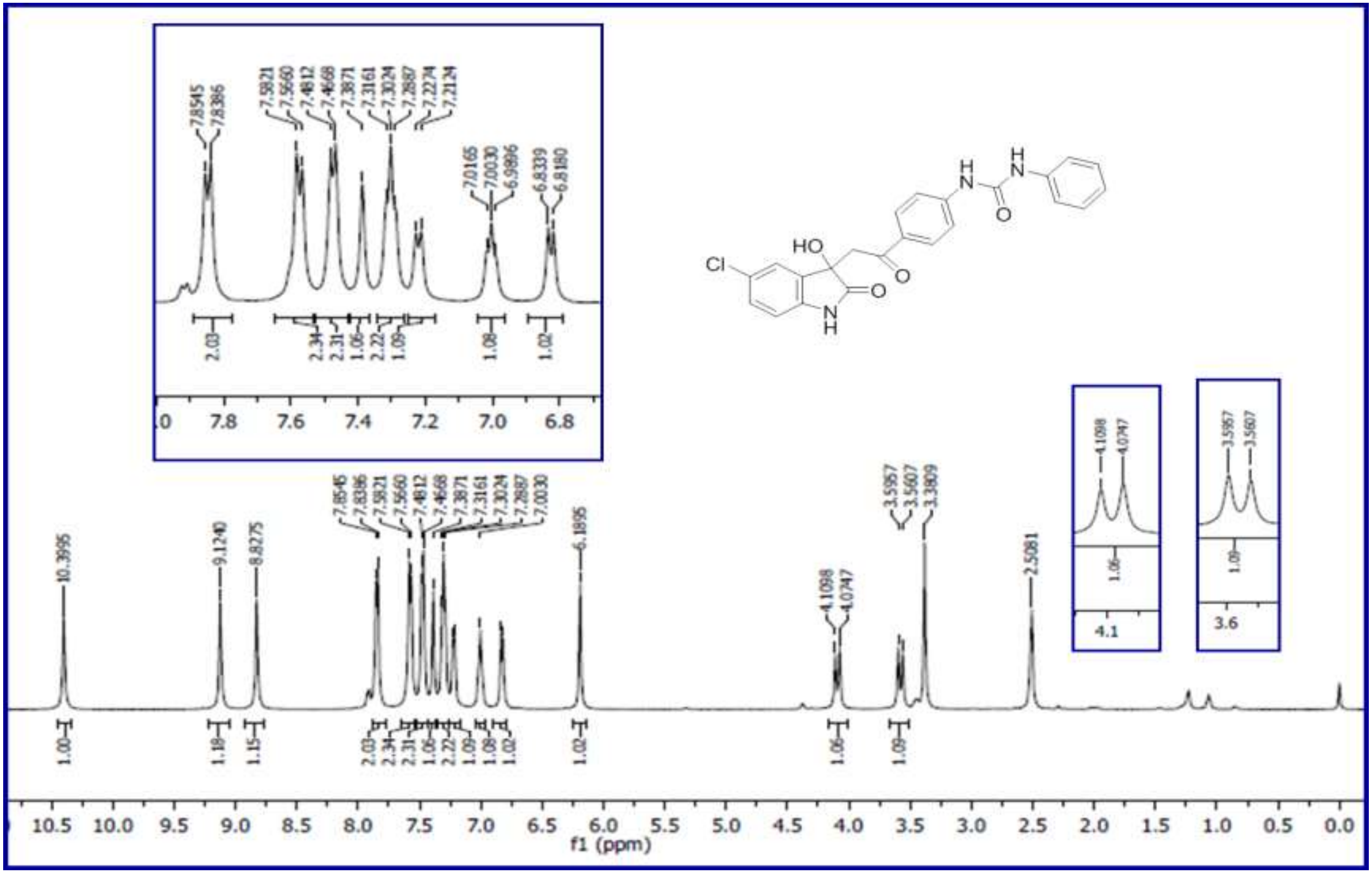

Fig. S17. ^{1}H-NMR spectrum of compound **11f** in DMSO-d_6.

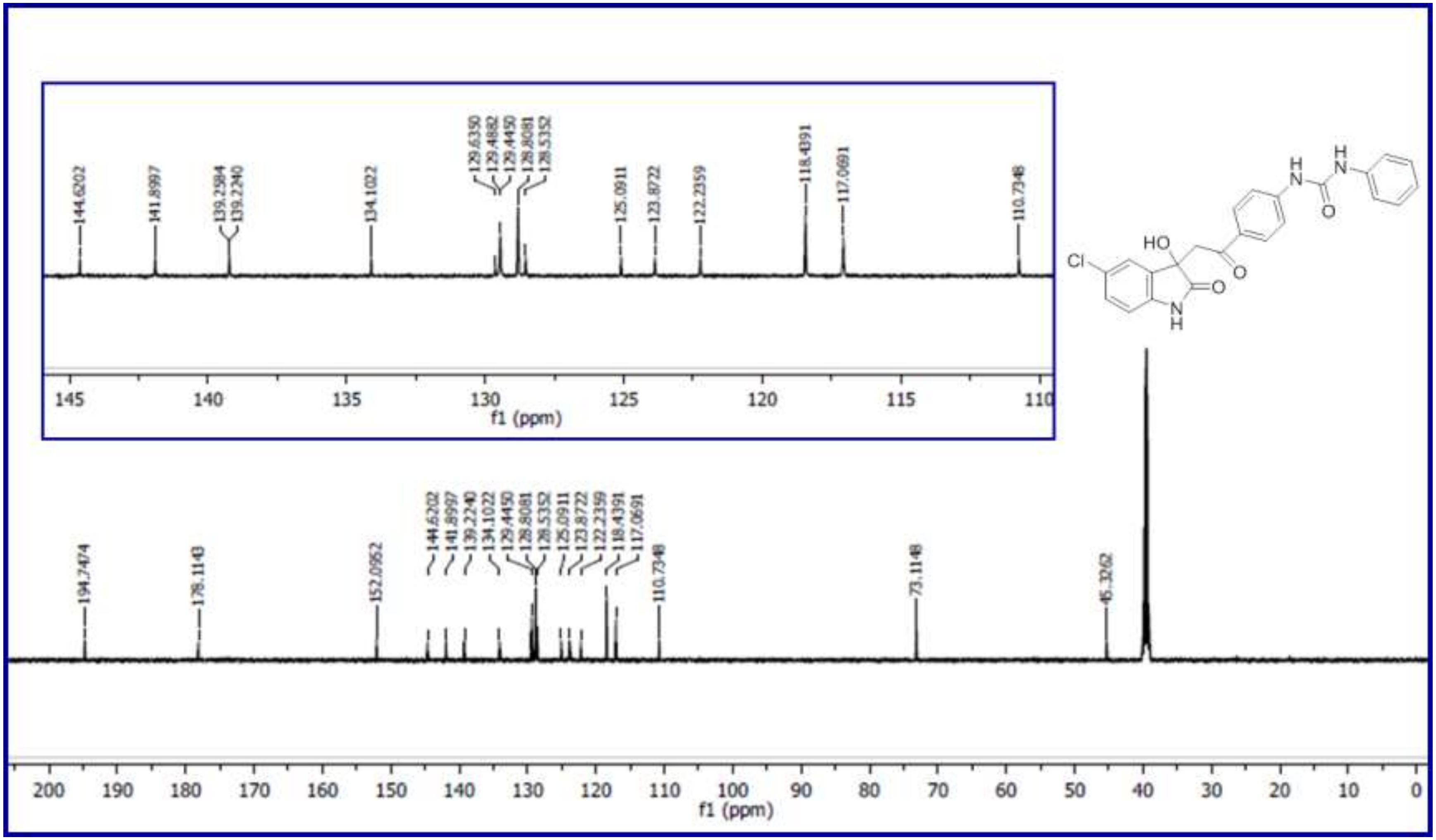

Fig. S18. ^{13}C-NMR spectrum of compound **11f** in DMSO-d_6.

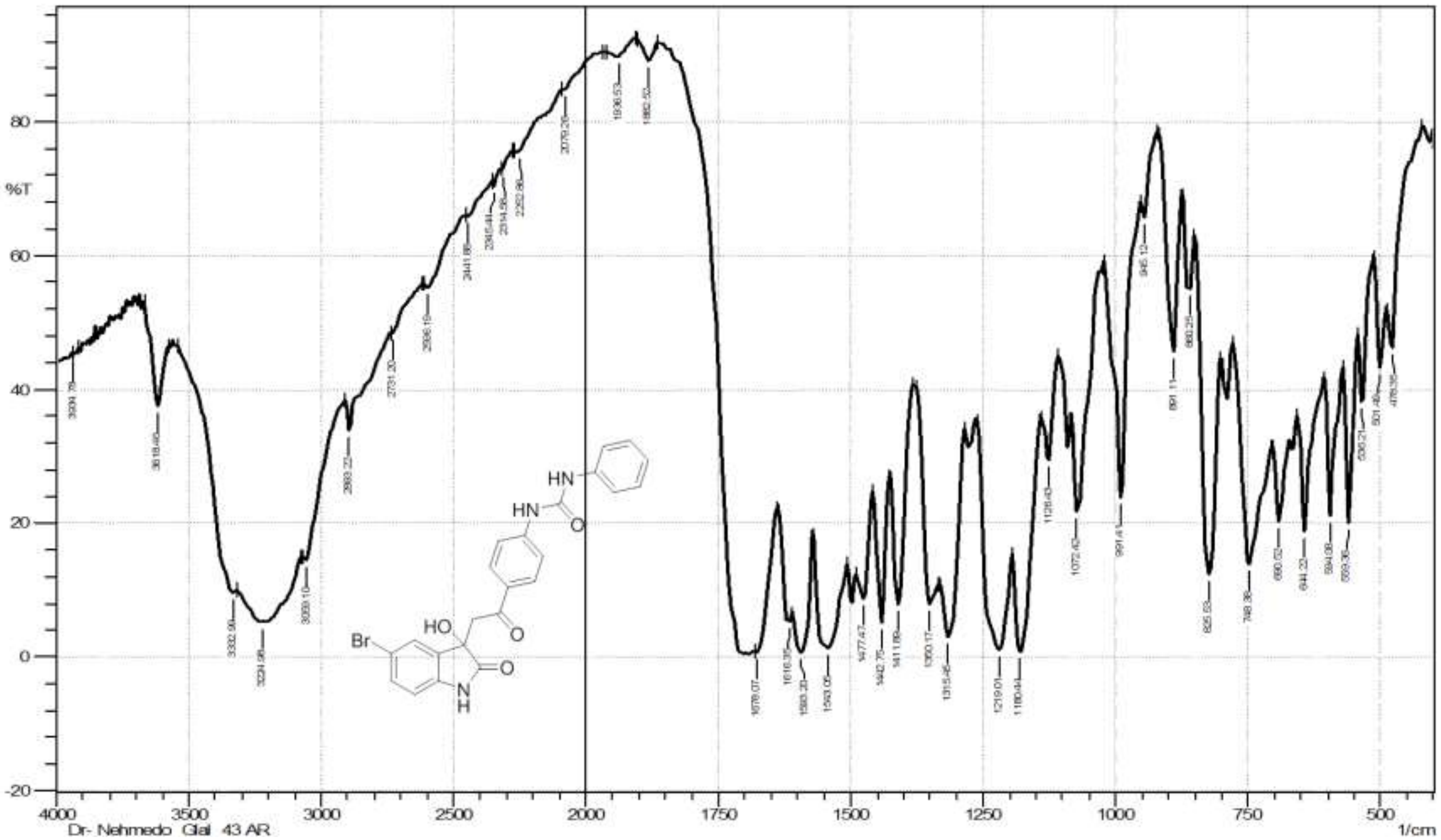

Fig. S19. IR spectrum of compound **11g** (KBr pellet).

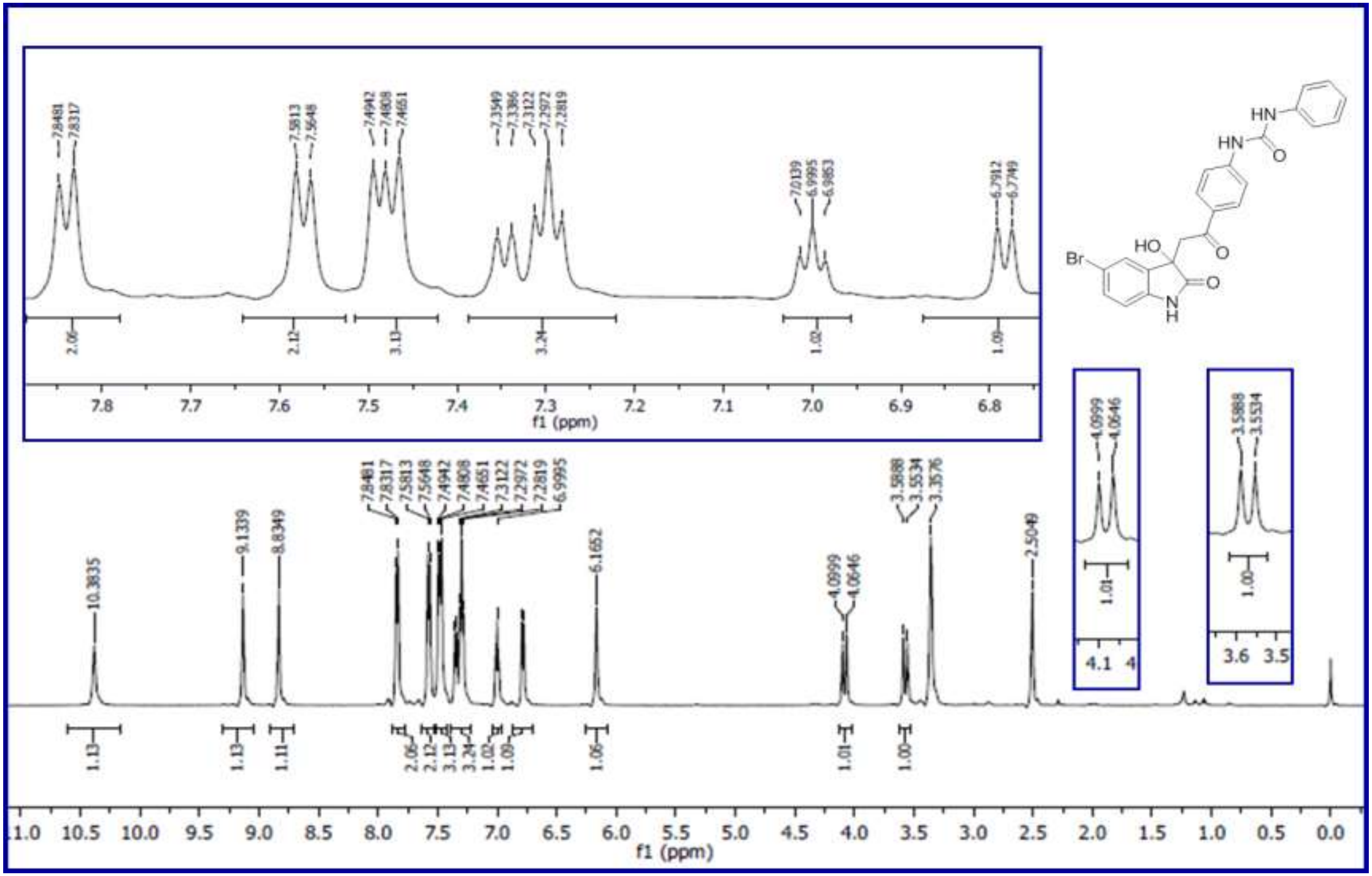

Fig. S20. ^{1}H-NMR spectrum of compound **11g** in DMSO-d_6.

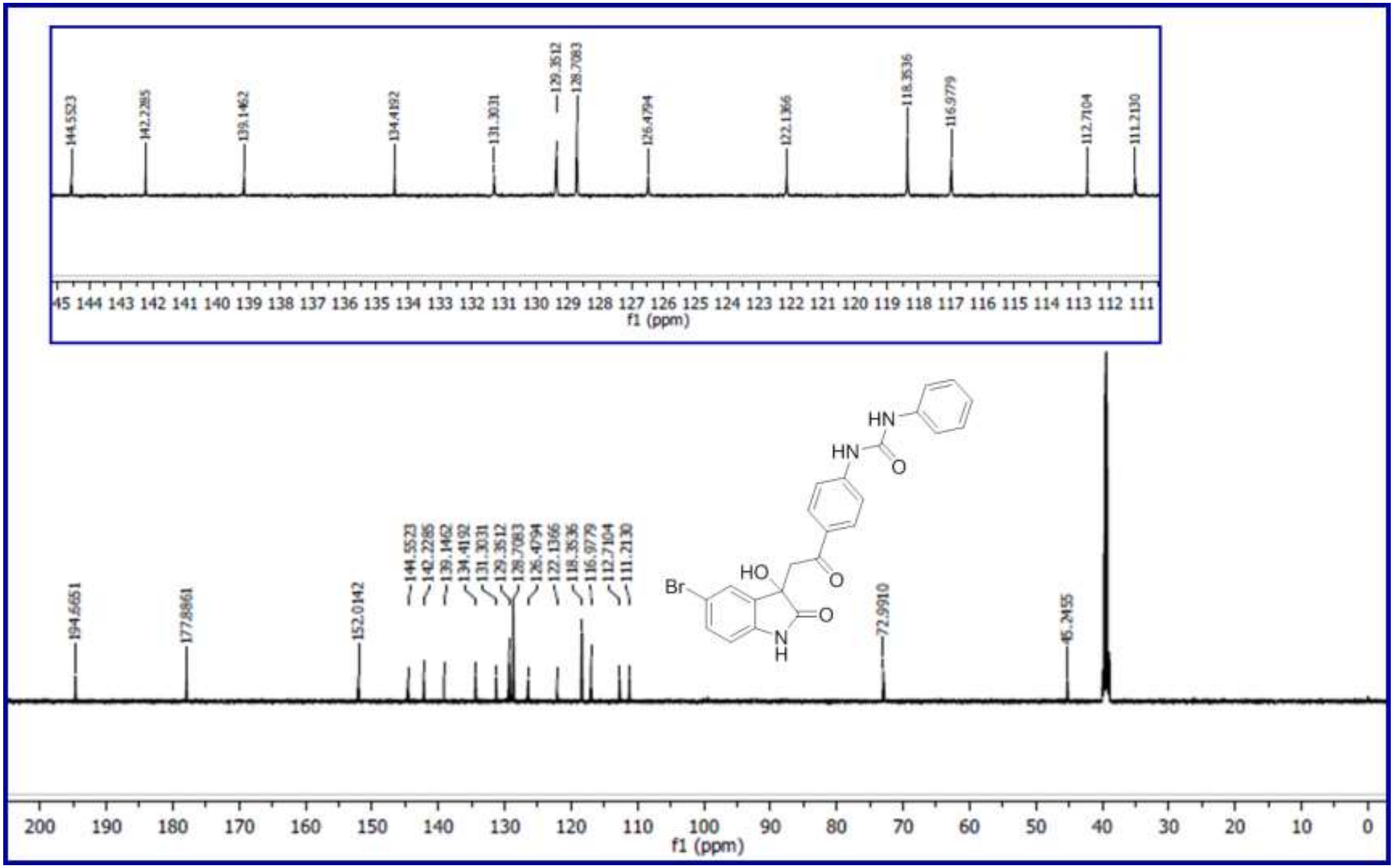

Fig. S21. ^{13}C-NMR spectrum of compound **11g** in DMSO-d_6.

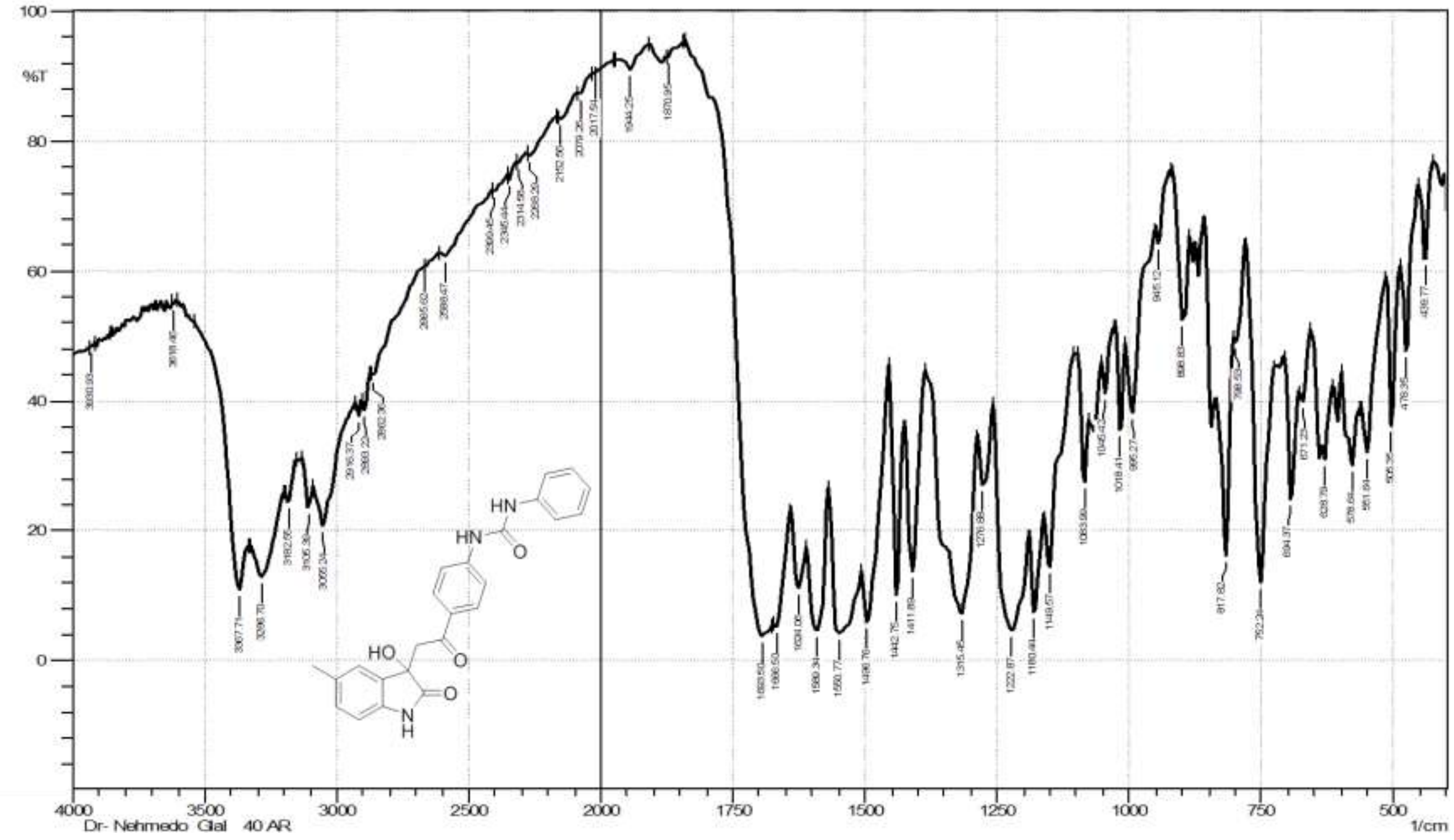

Fig. S22. IR spectrum of compound **11h** (KBr pellet).

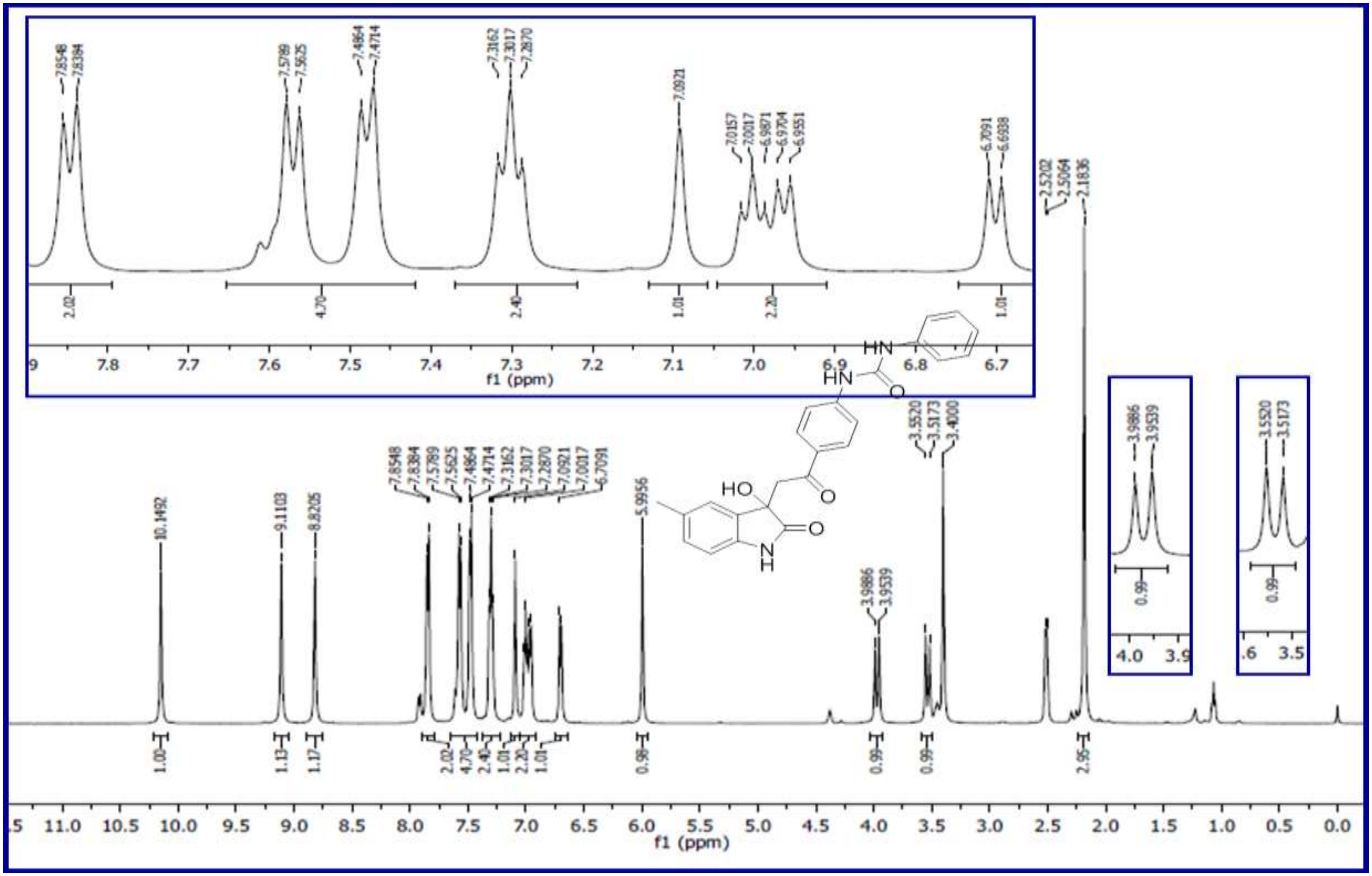

Fig. S23. ^{1}H-NMR spectrum of compound **11h** in DMSO-d_6.

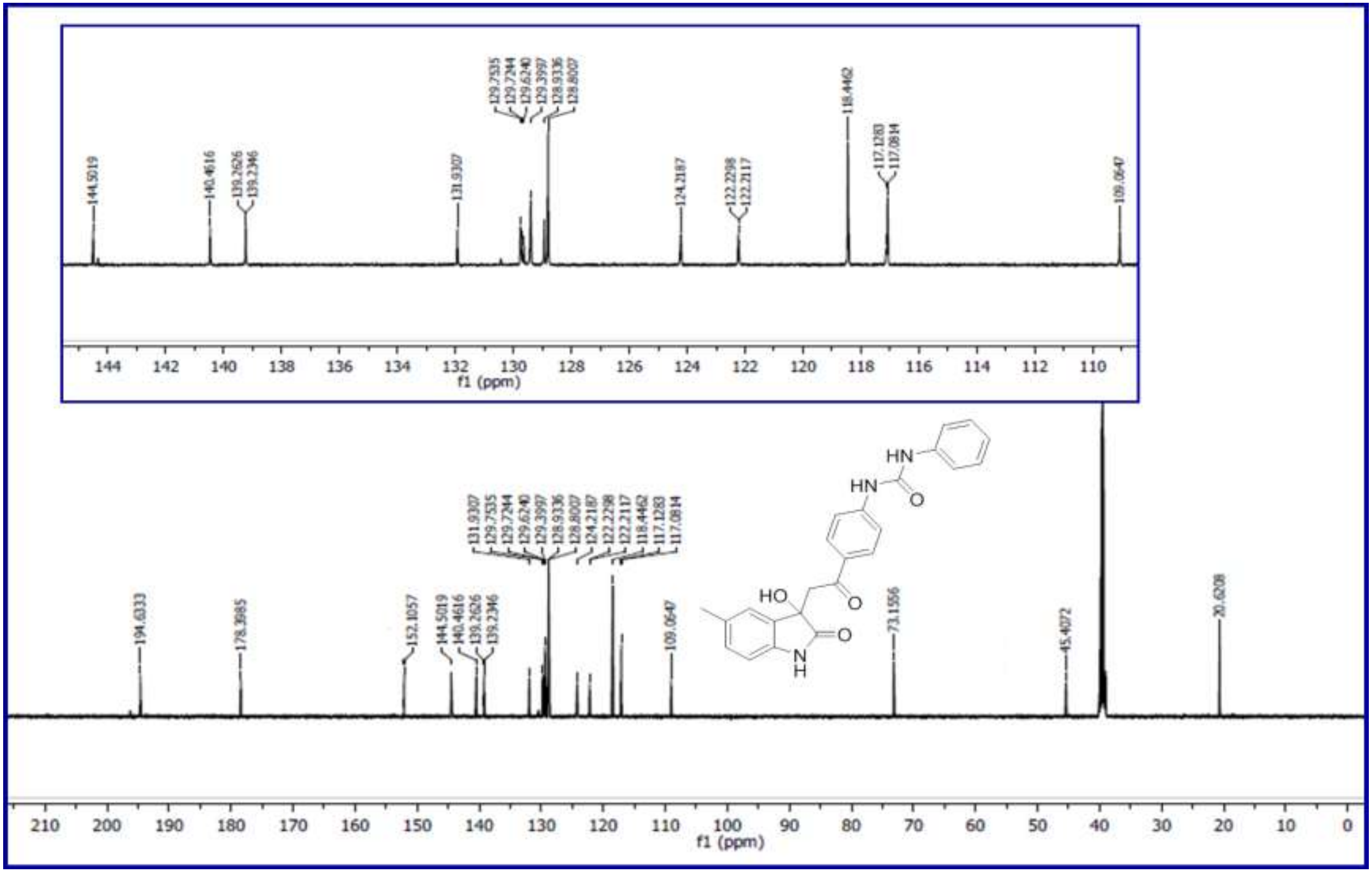

Fig. S24. ^{13}C-NMR spectrum of compound **11h** in DMSO-d_6.

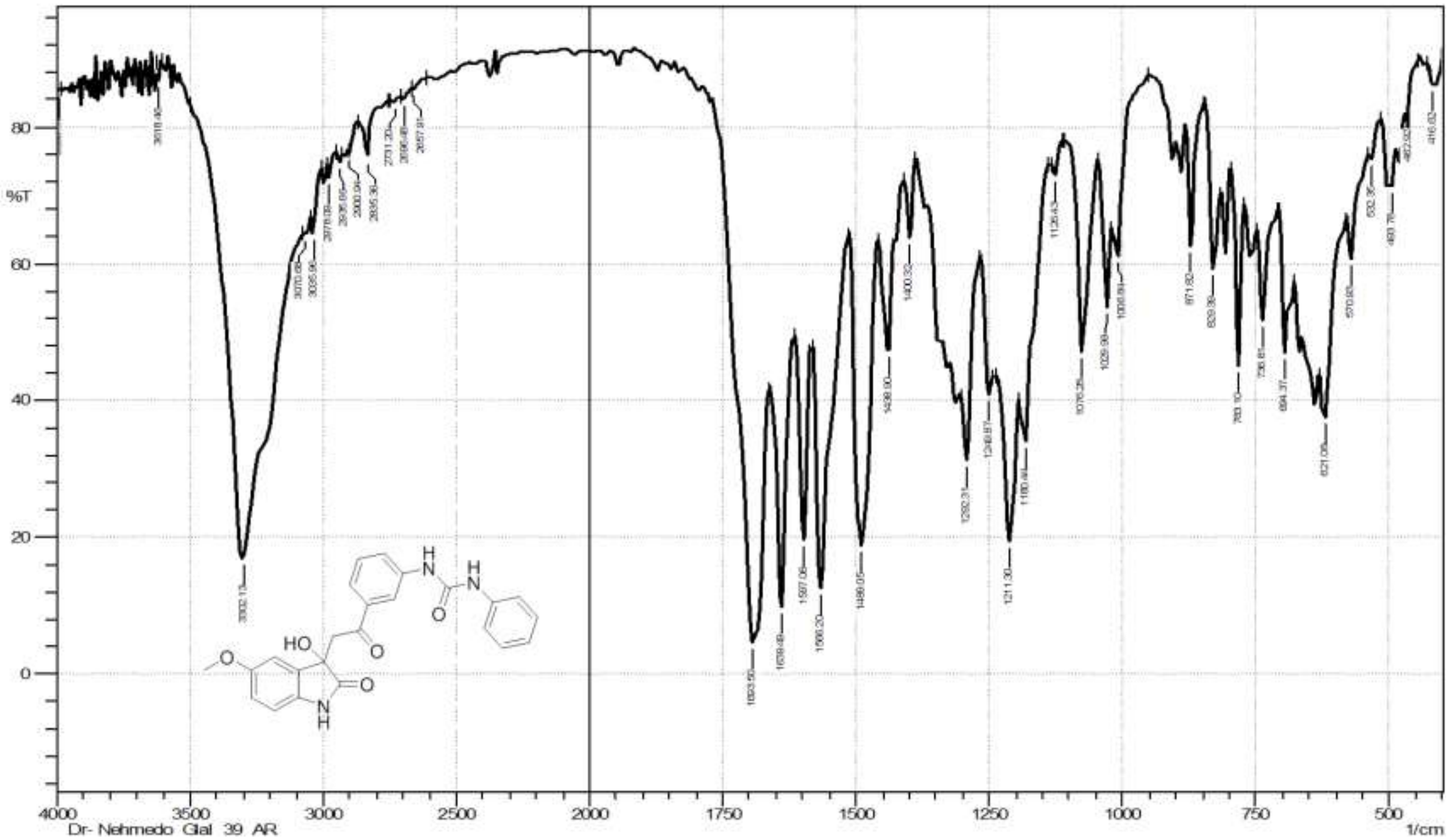

Fig. S25. IR spectrum of compound **11i** (KBr pellet).

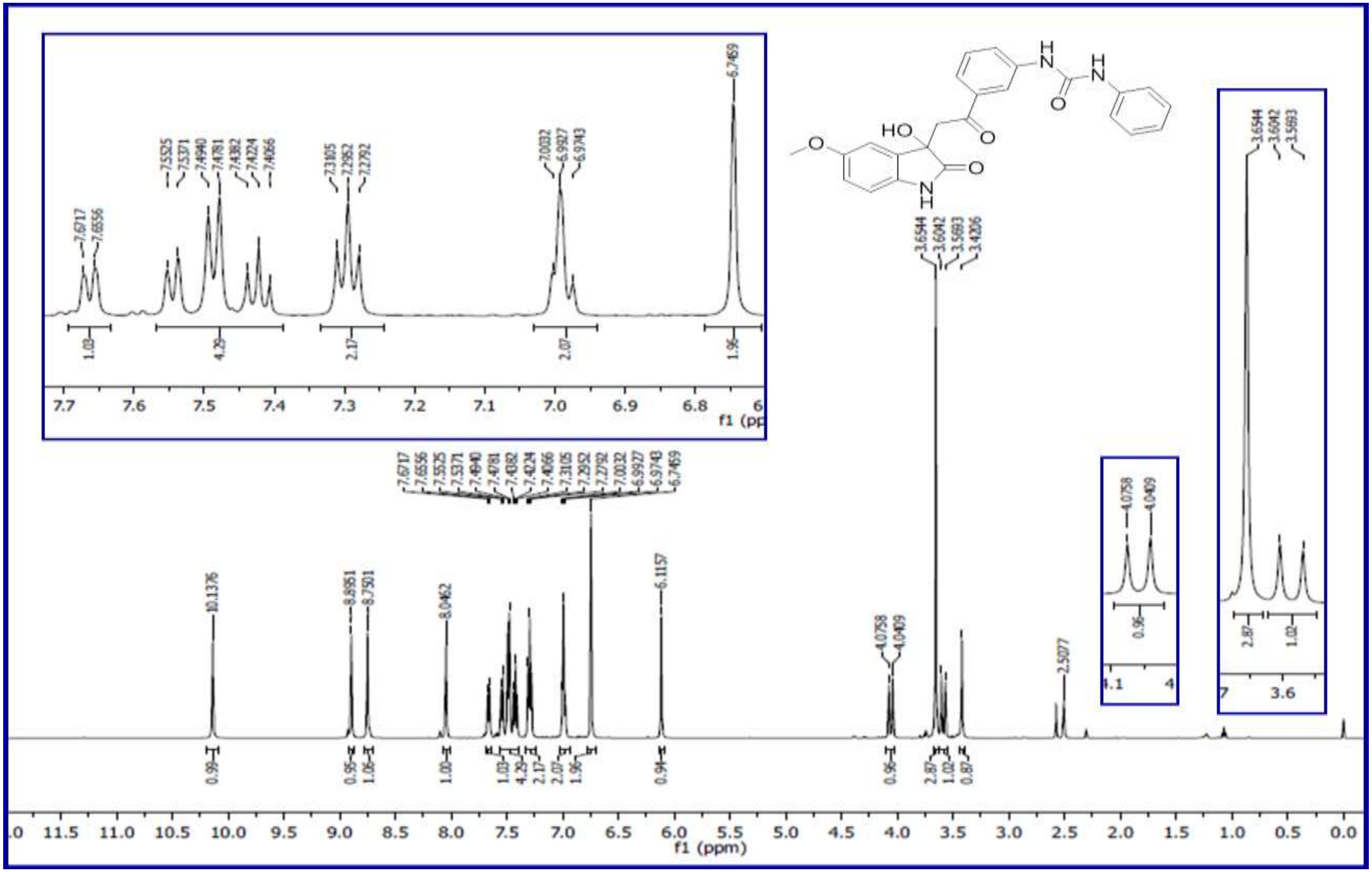

Fig. S26. ^{1}H-NMR spectrum of compound **11i** in DMSO-d_6.

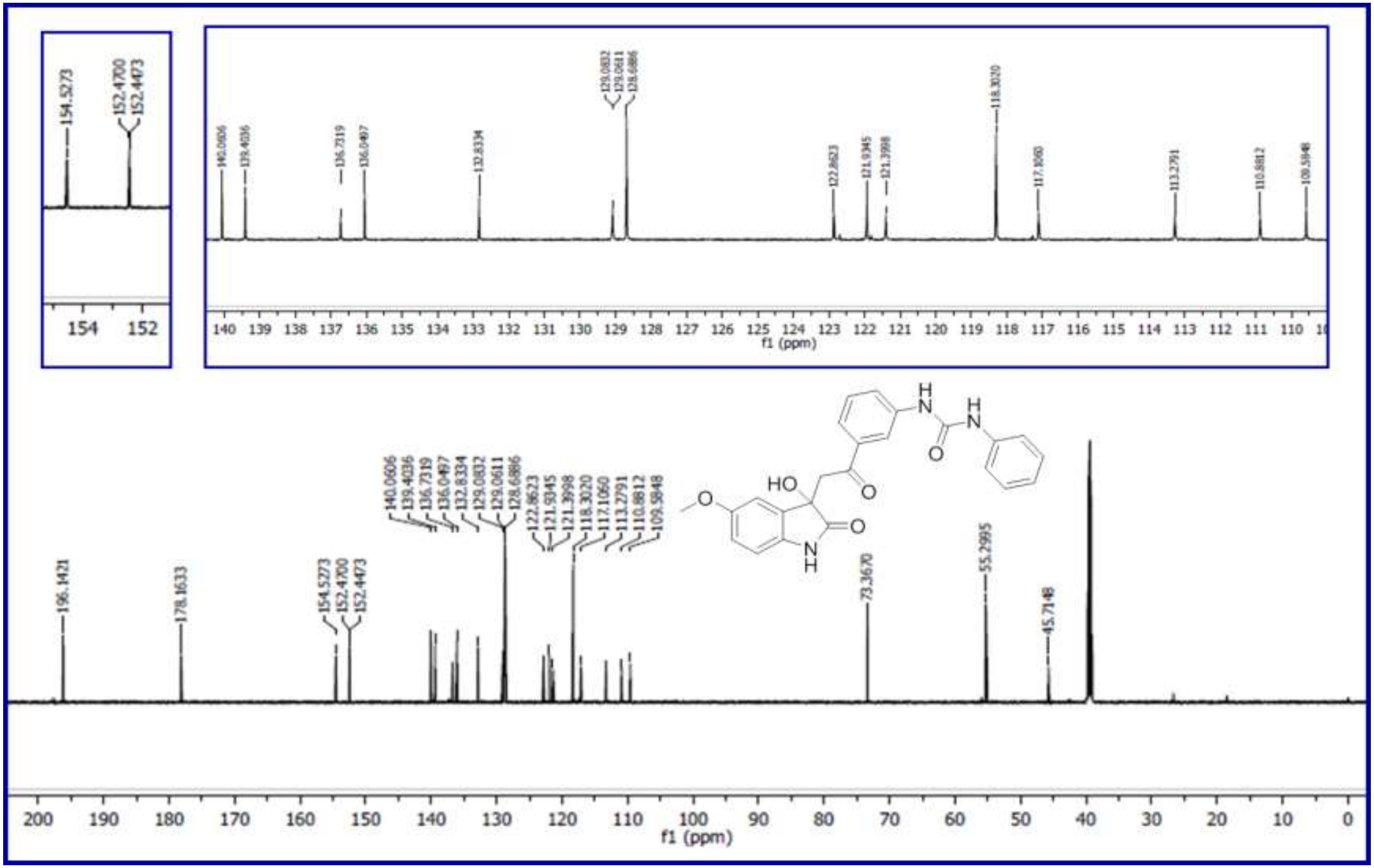

Fig. S27. ^{13}C-NMR spectrum of compound **11i** in DMSO-d_6.

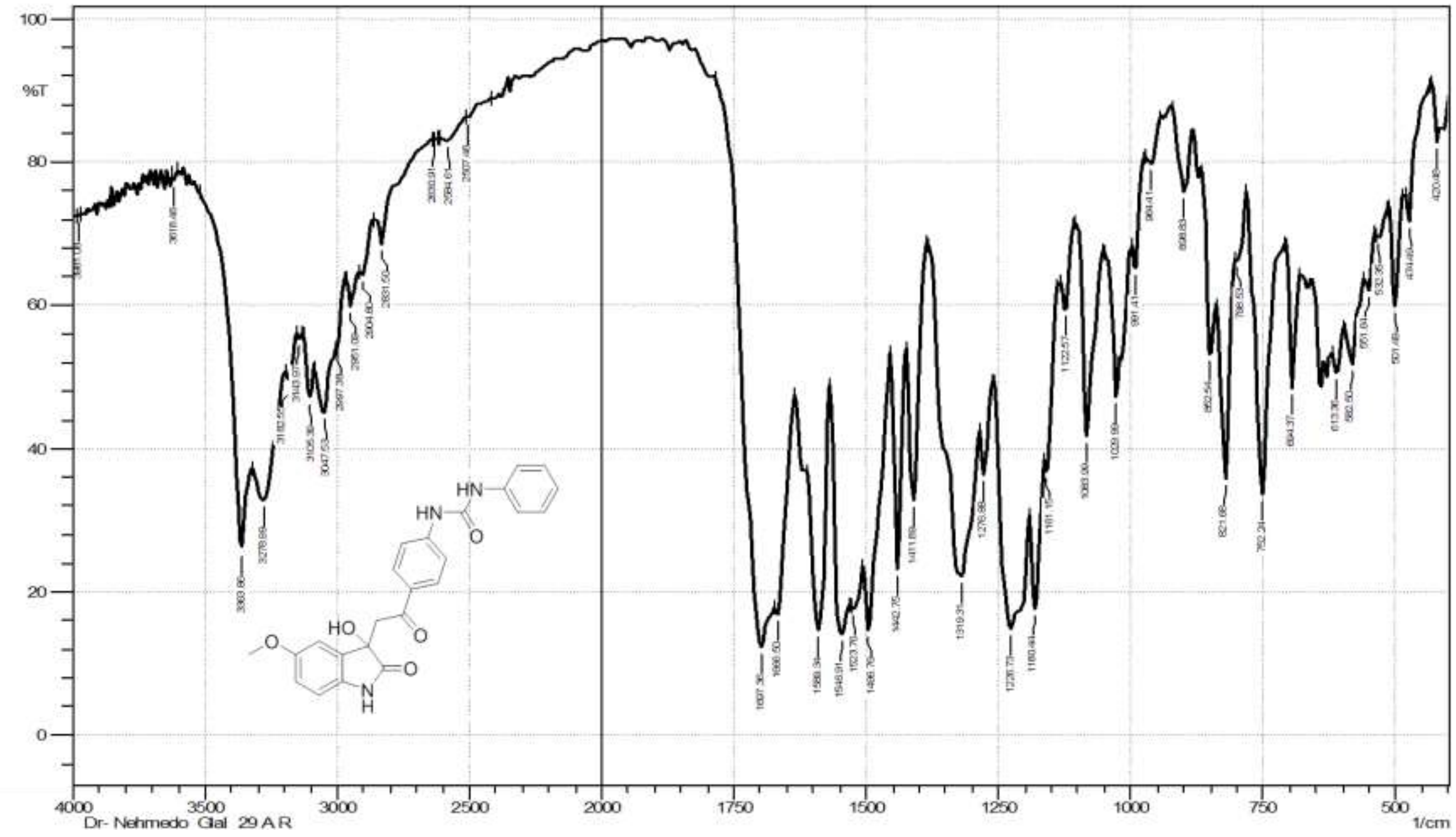

Fig. S28. IR spectrum of compound **11j** (KBr pellet).

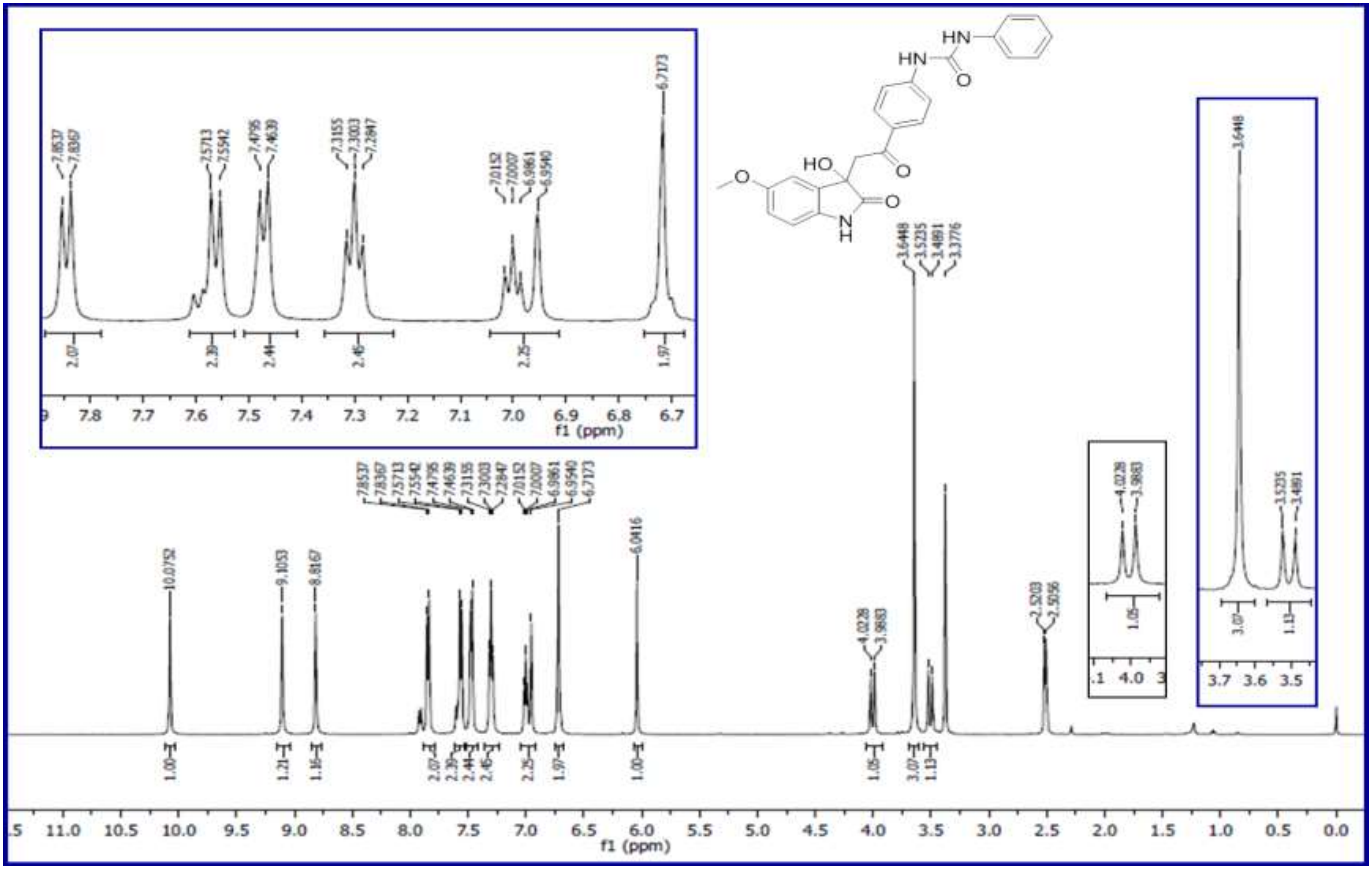

Fig. S29. ^{1}H-NMR spectrum of compound **11j** in DMSO-d_6.

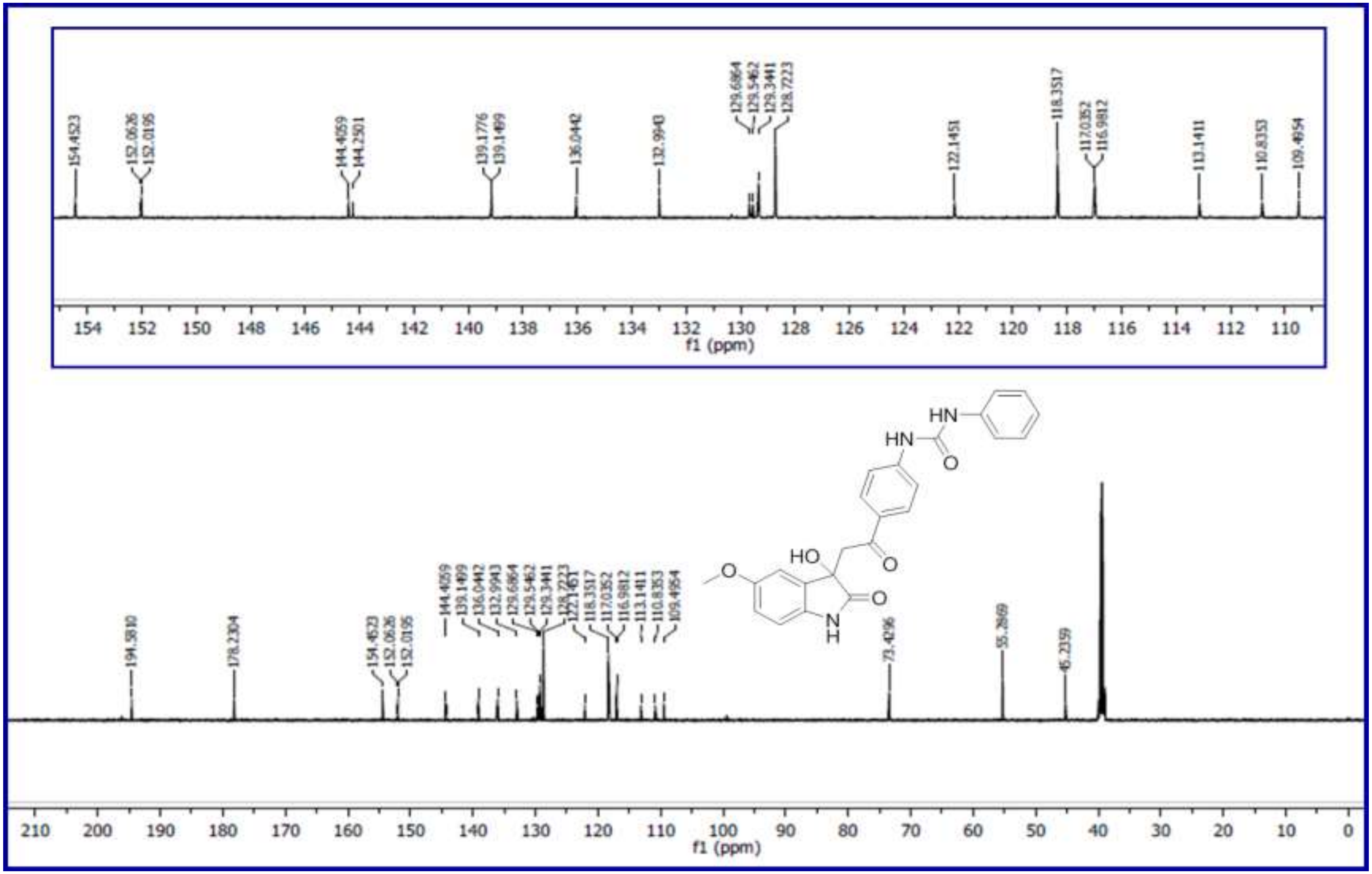

Fig. S30. ^{13}C-NMR spectrum of compound **11j** in DMSO-d_6.

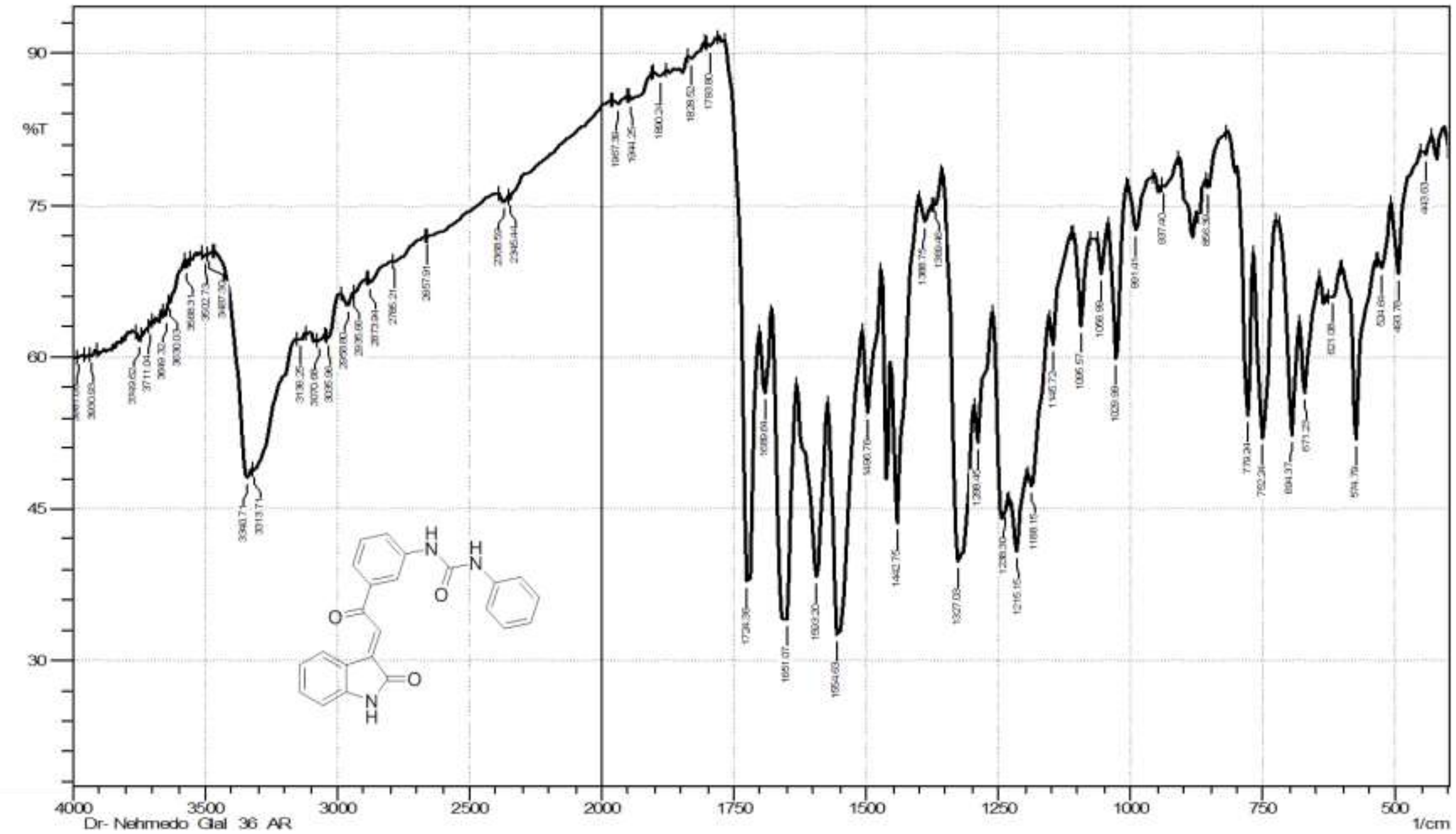

Fig. S31. IR spectrum of compound **12a** (KBr pellet).

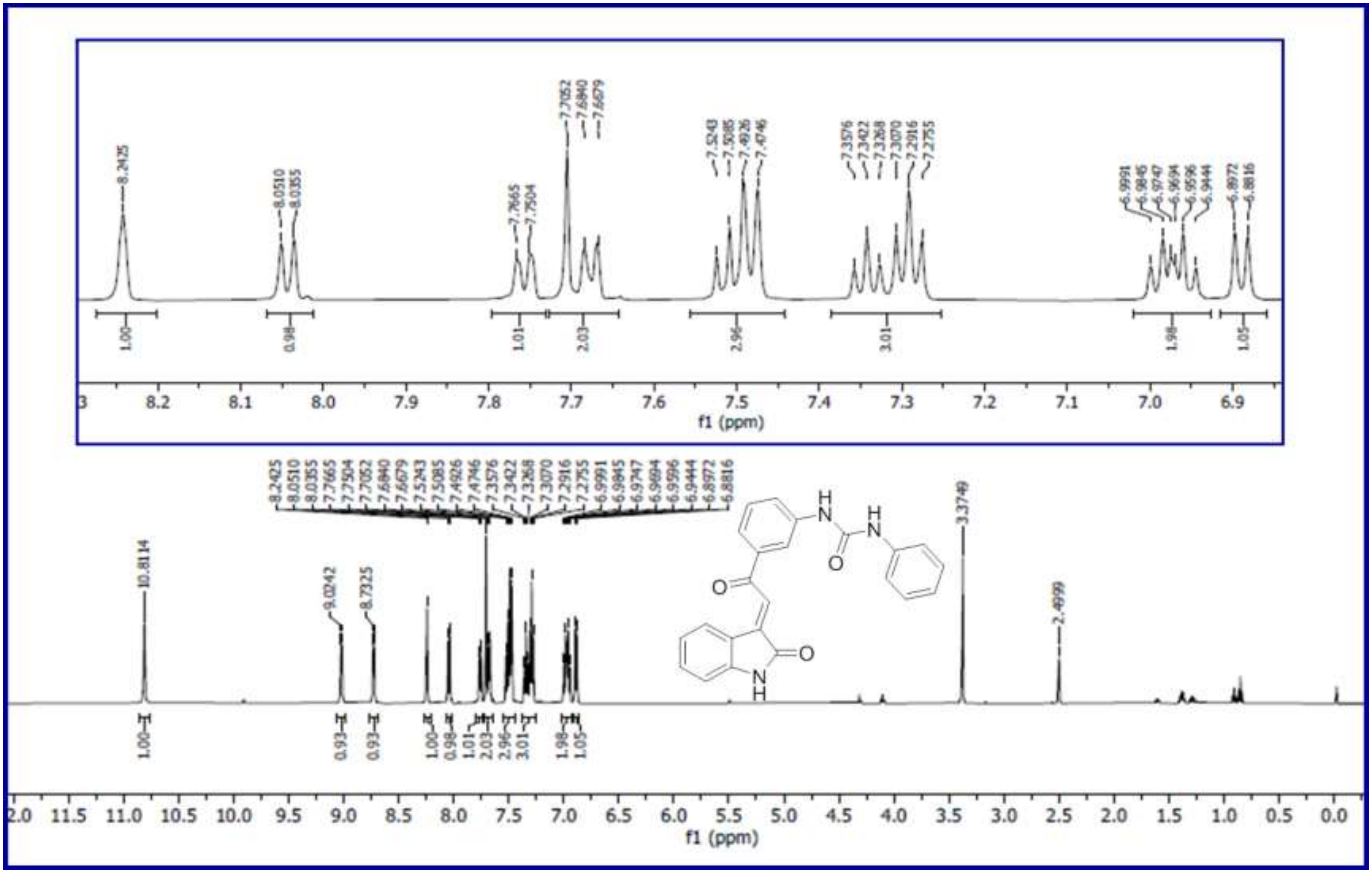

Fig. S32. ^{1}H-NMR spectrum of compound **12a** in DMSO-d_6.

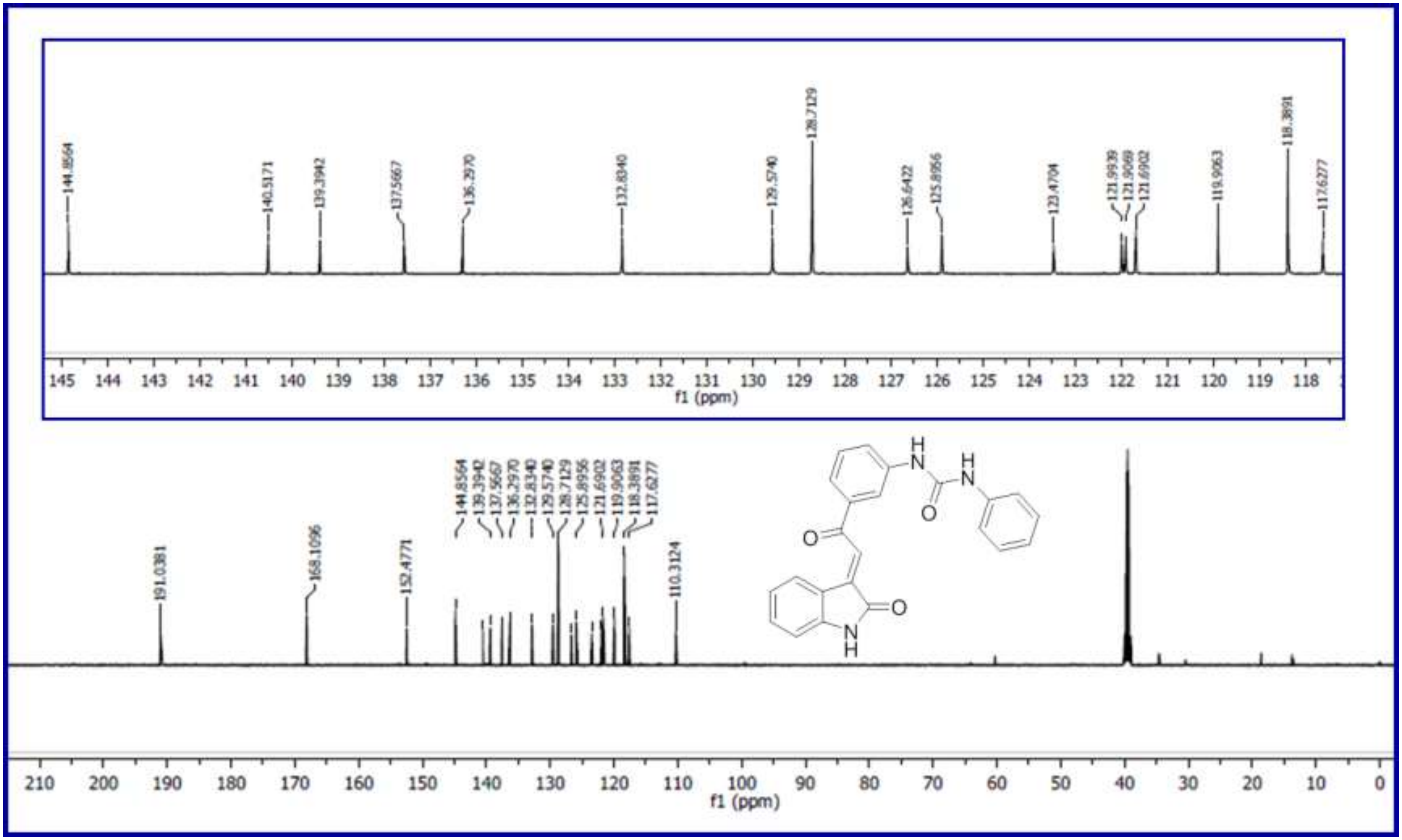

Fig. S33. ^{13}C-NMR spectrum of compound **12a** in DMSO-d_6.

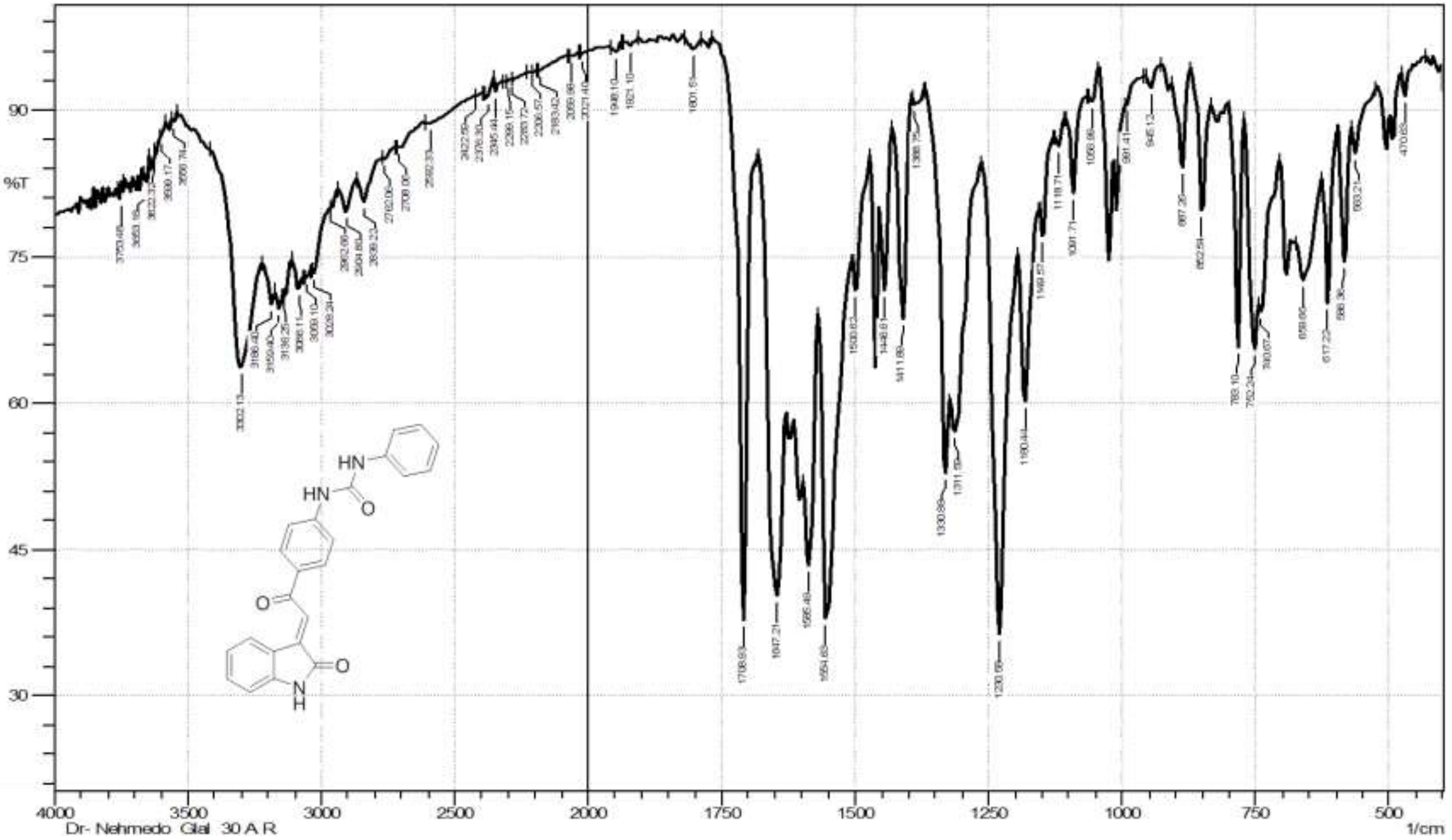

Fig. S34. IR spectrum of compound **12b** (KBr pellet).

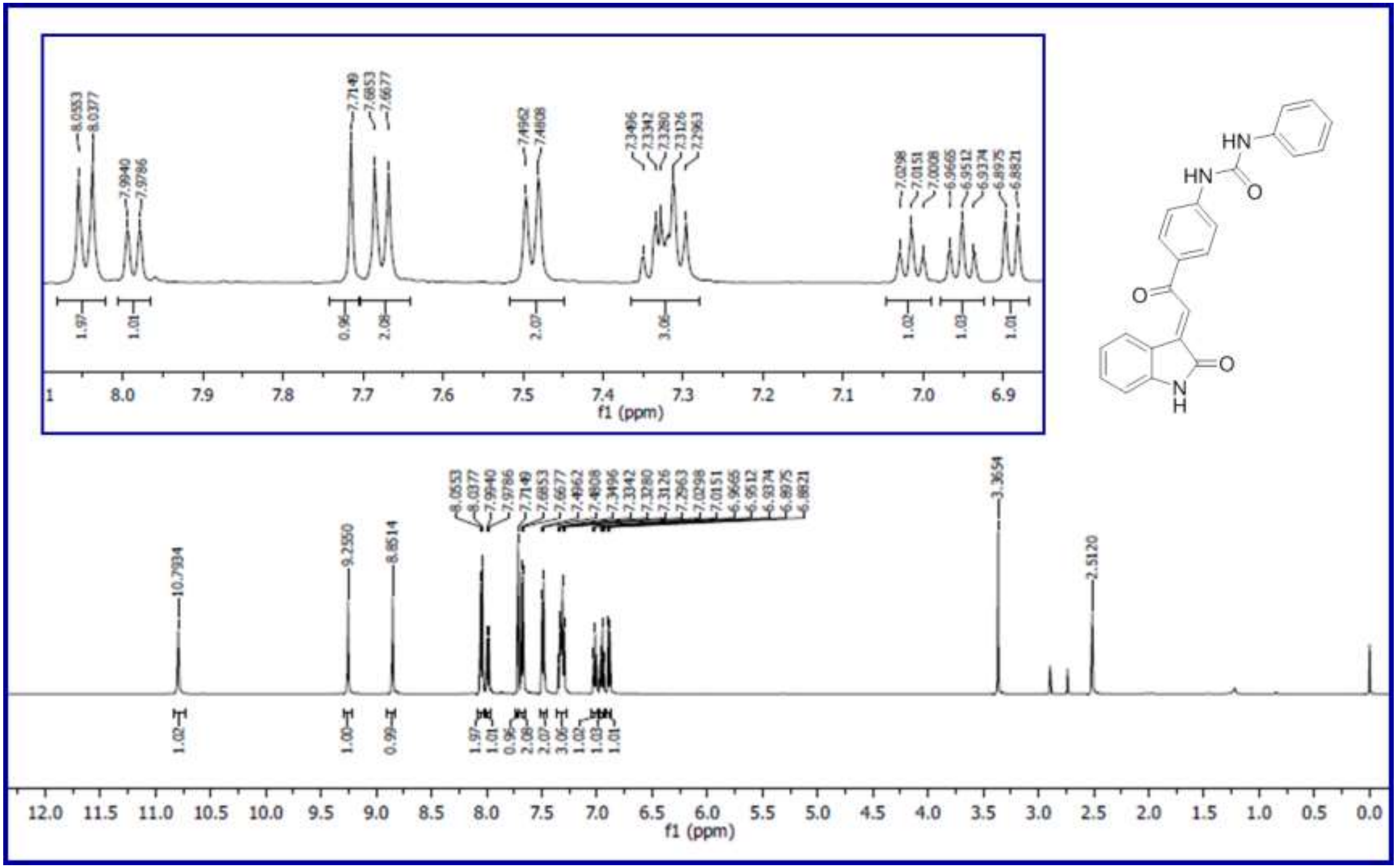

Fig. S35. ^{1}H-NMR spectrum of compound **12b** in DMSO-d_6.

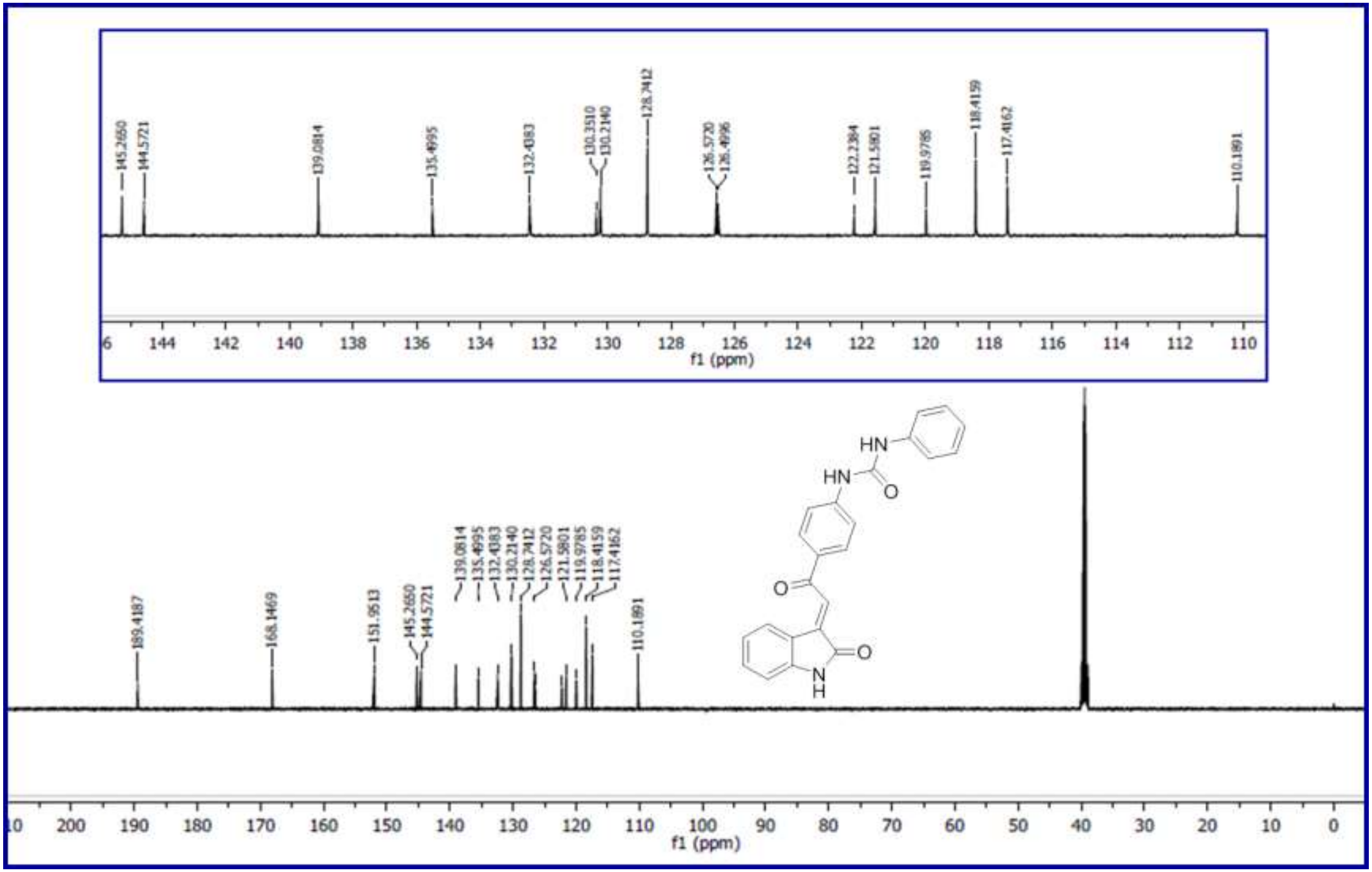

Fig. S36. ^{13}C-NMR spectrum of compound **12b** in DMSO-d_6.

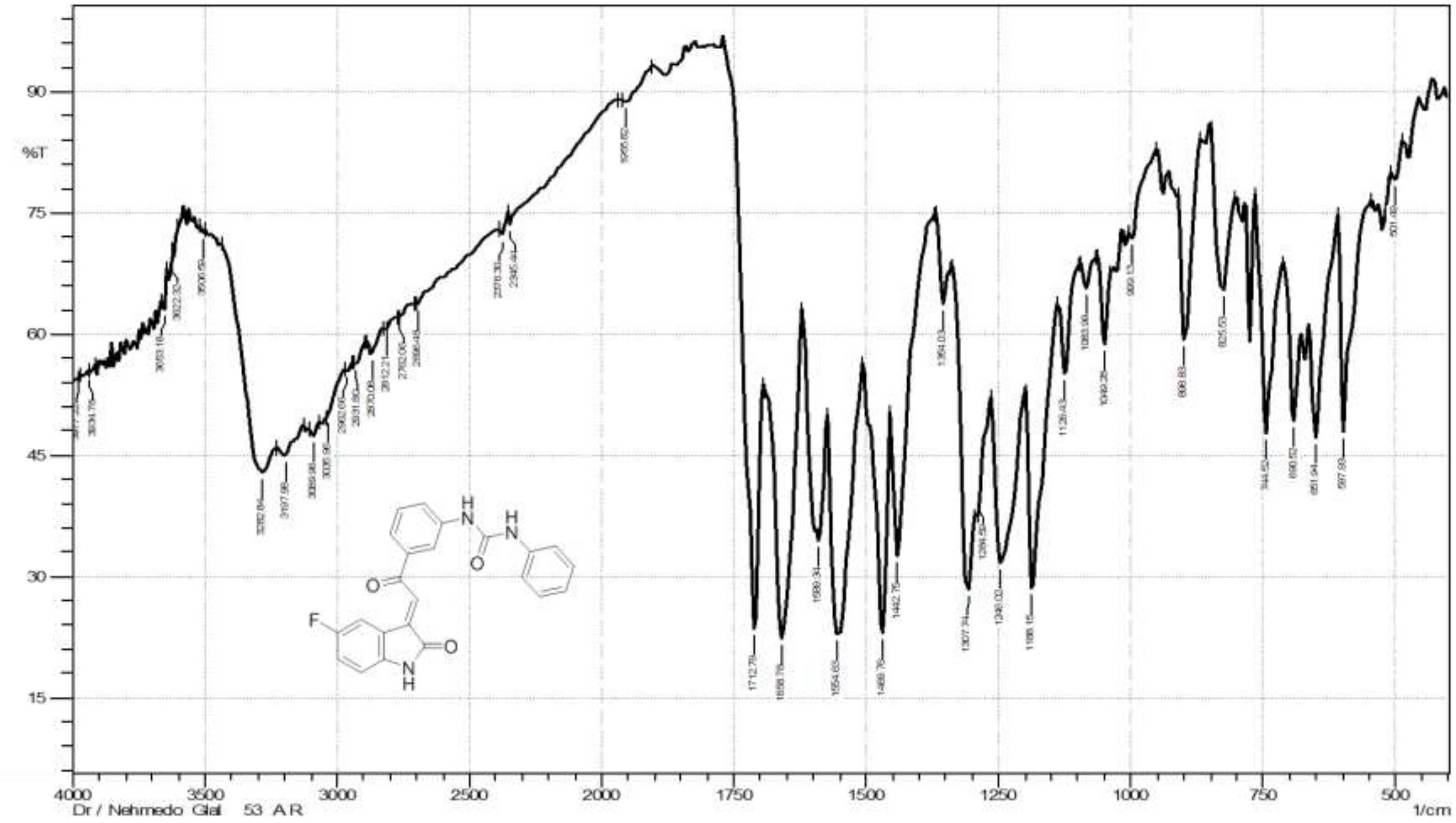

Fig. S37. IR spectrum of compound **12c** (KBr pellet).

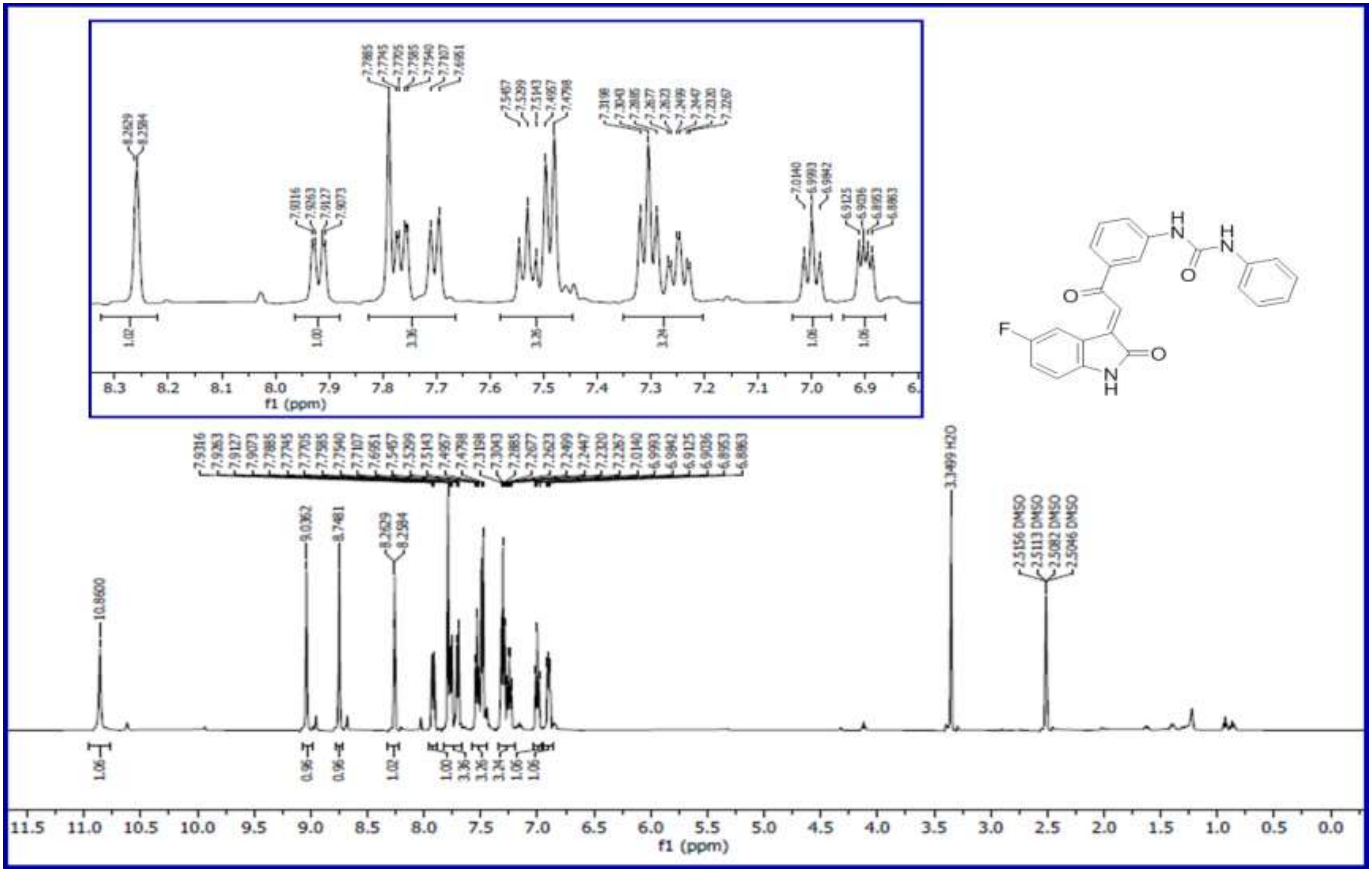

Fig. S38. ^{1}H-NMR spectrum of compound **12c** in DMSO-d_6.

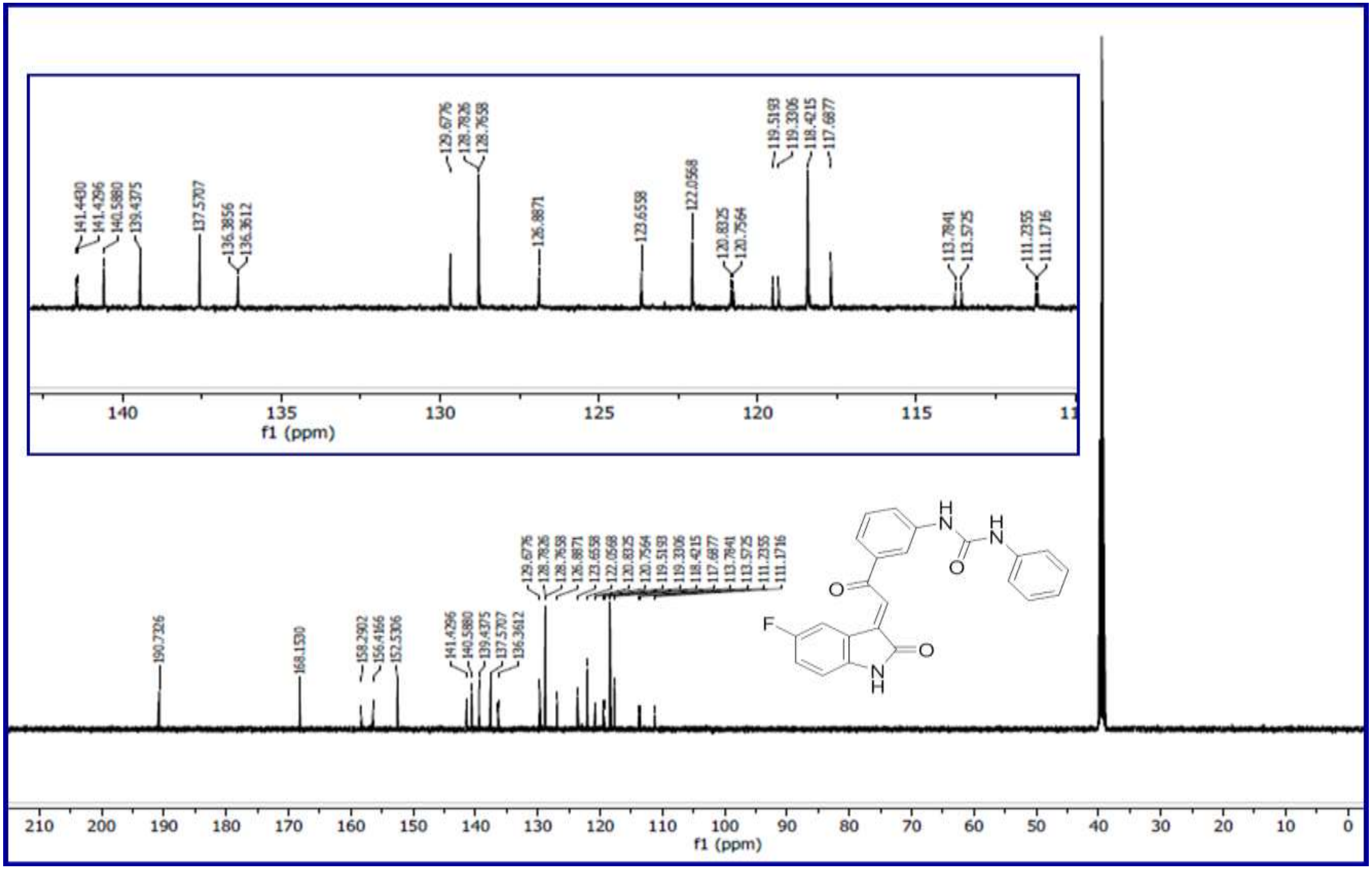

Fig. S39. ^{13}C-NMR spectrum of compound **12c** in DMSO-d_6.

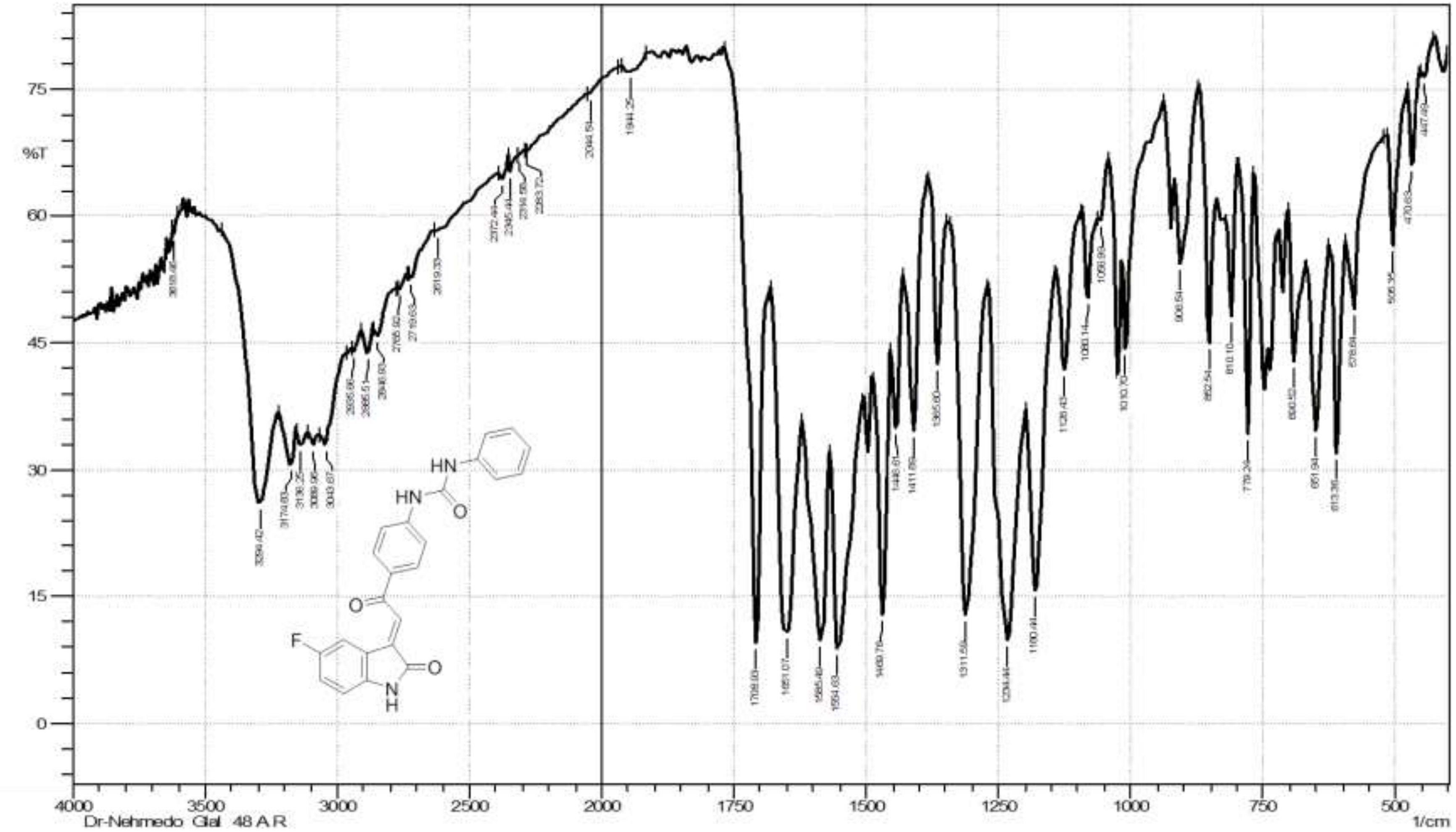

Fig. S40. IR spectrum of compound **12d** (KBr pellet).

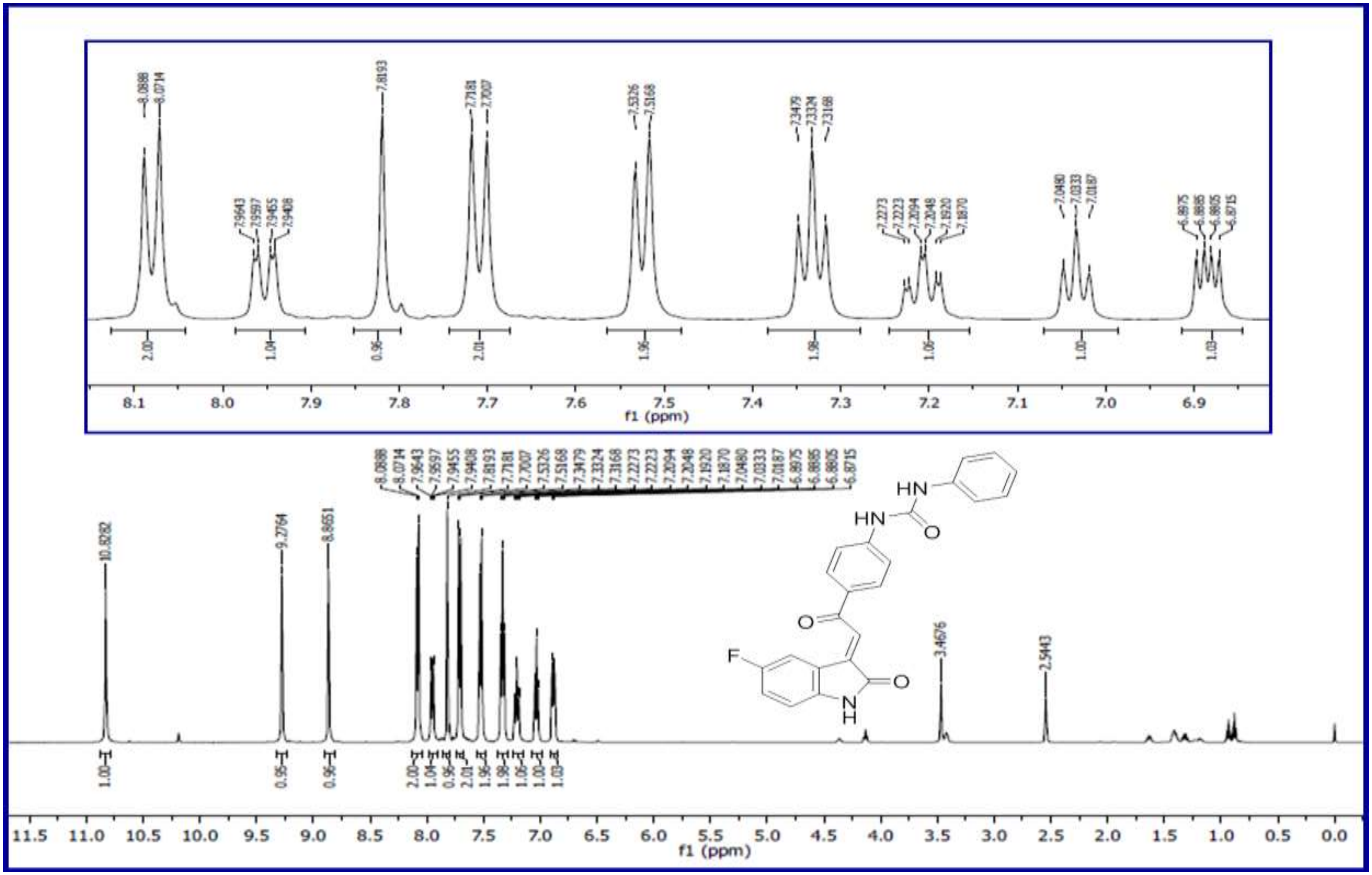

Fig. S41. ^{1}H-NMR spectrum of compound **12d** in DMSO-d_6.

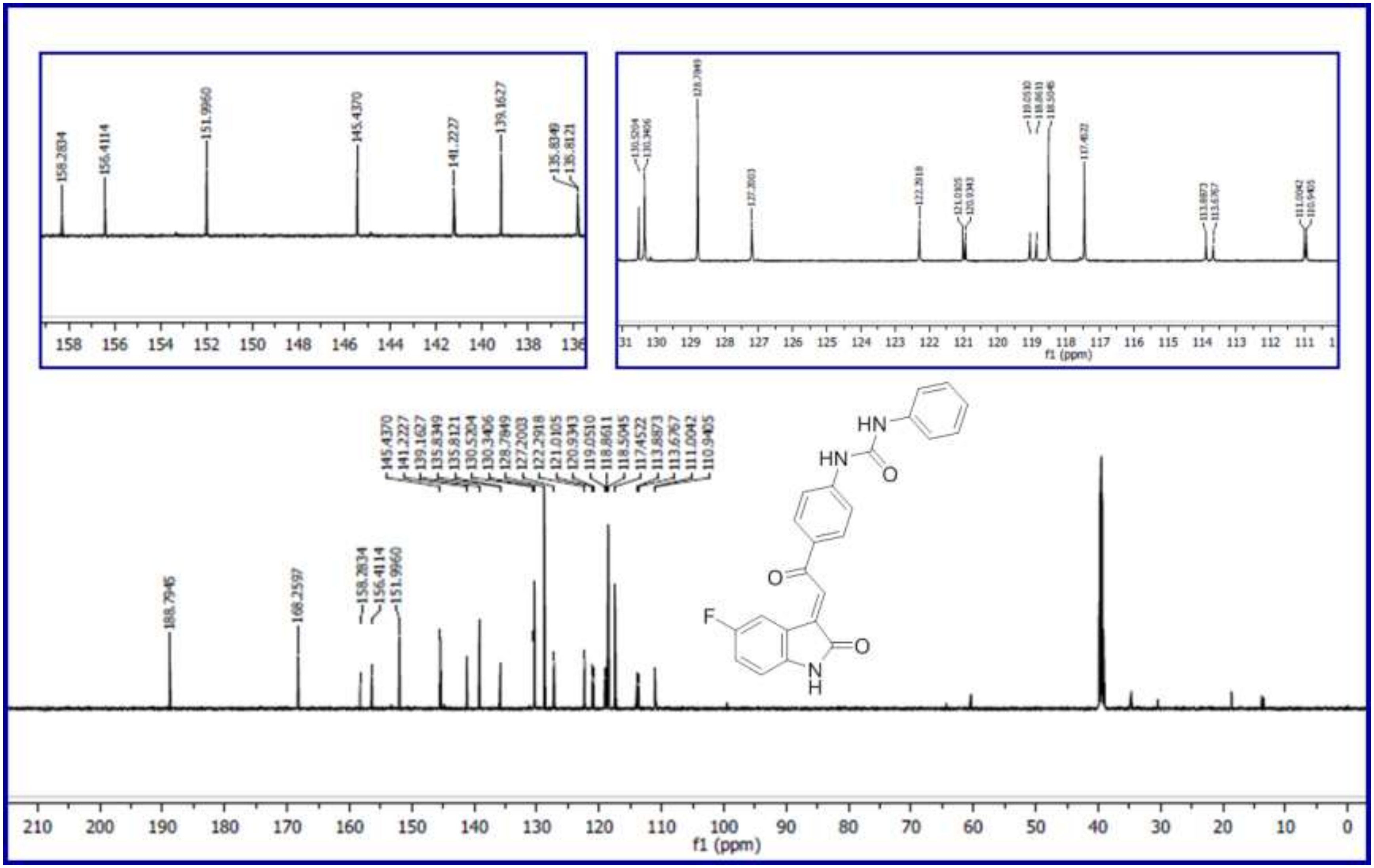

Fig. S42. ^{13}C-NMR spectrum of compound **12d** in DMSO-d_6.

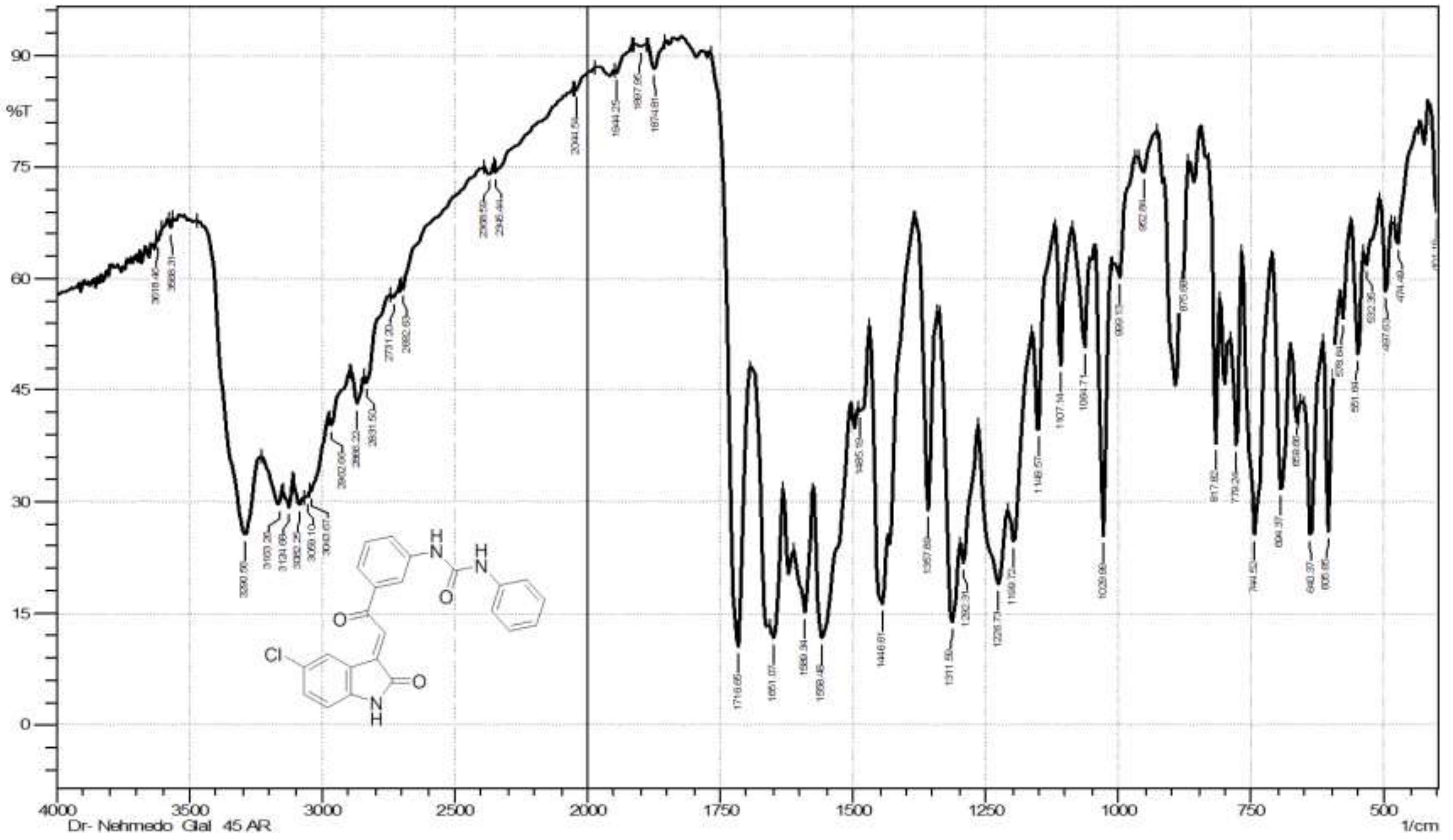

Fig. S43. IR spectrum of compound **12e** (KBr pellet).

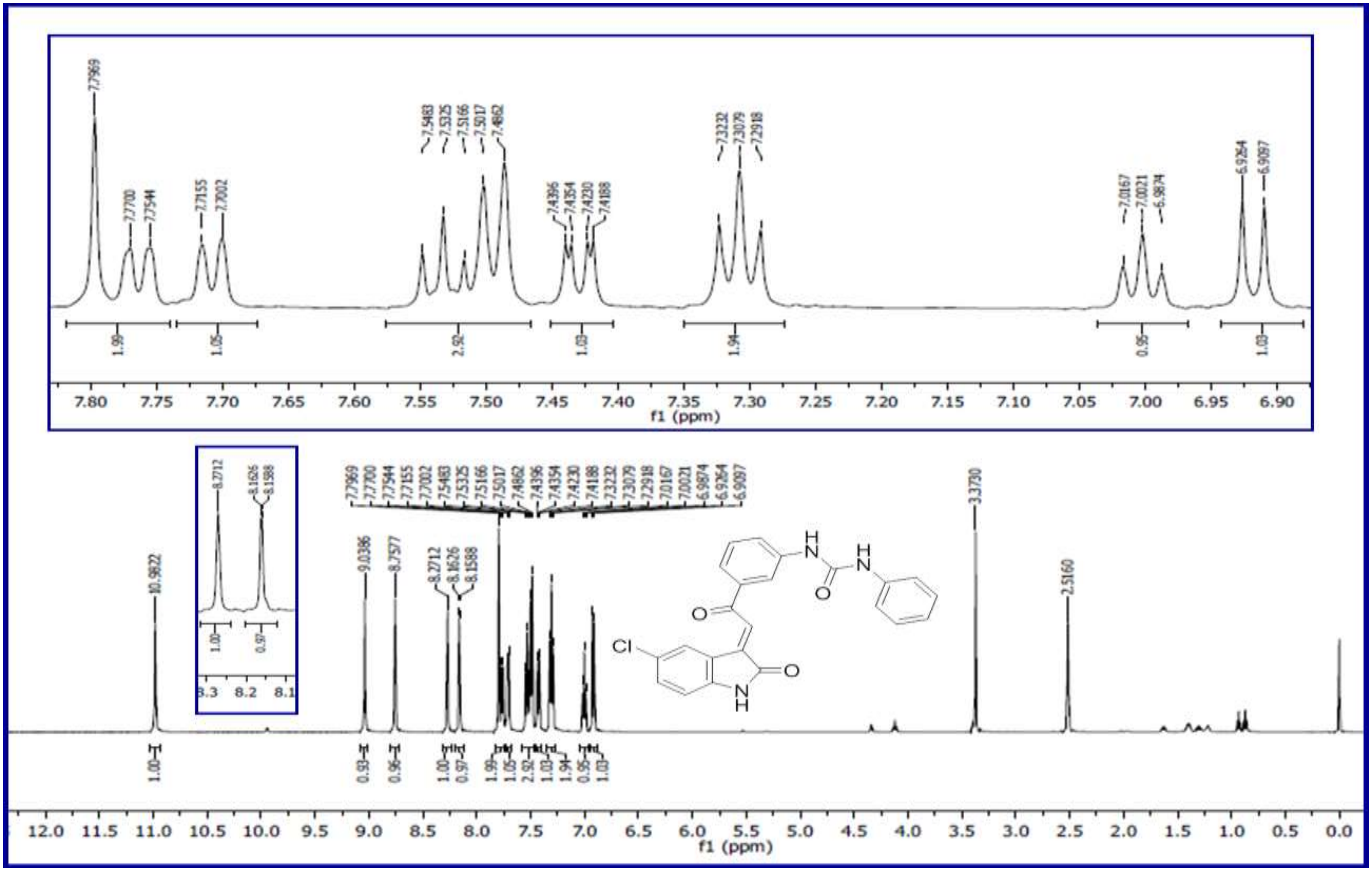

Fig. S44. ^{1}H-NMR spectrum of compound **12e** in DMSO-d_6.

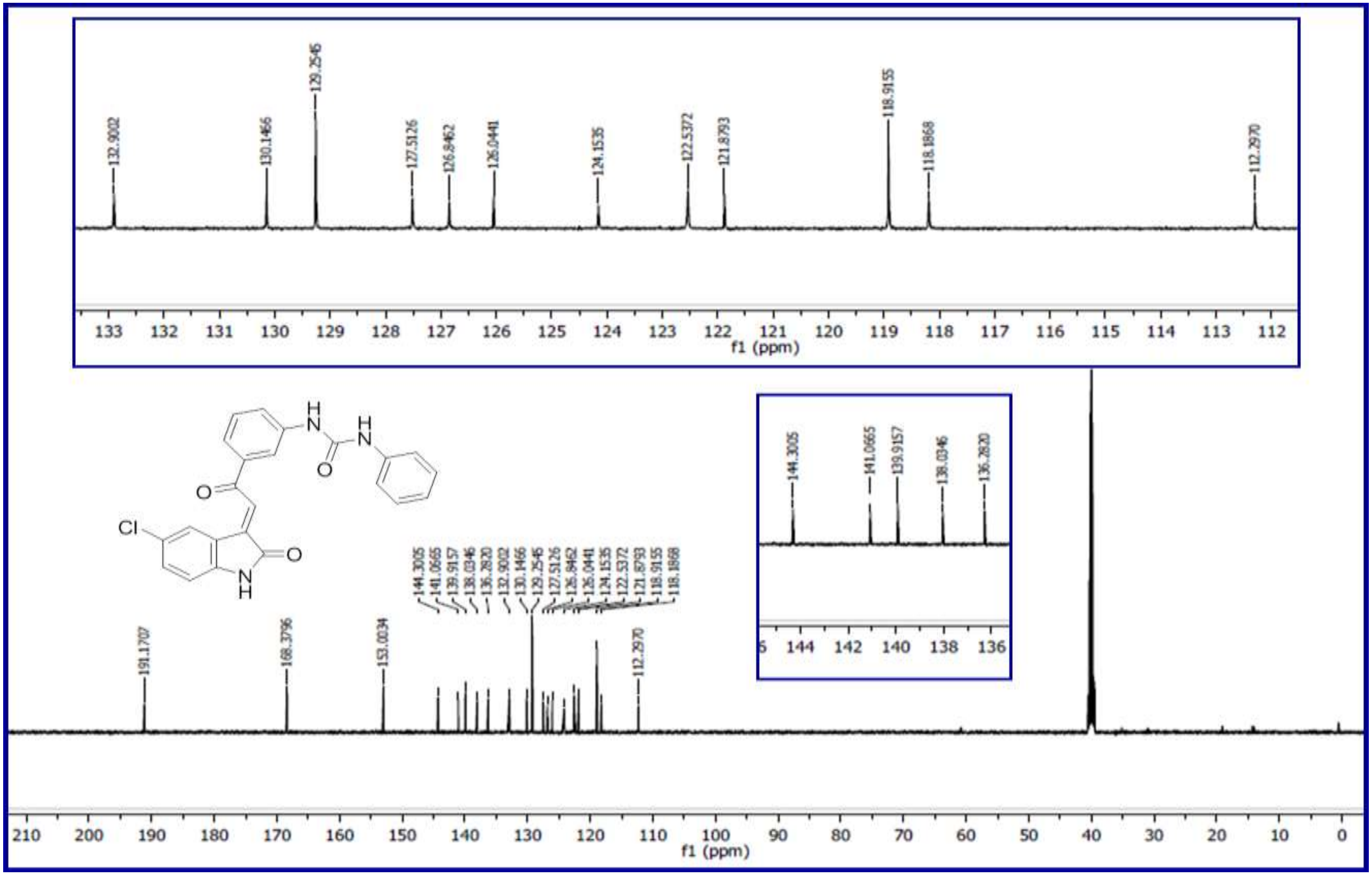

Fig. S45. ^{13}C-NMR spectrum of compound **12e** in DMSO-d_6.

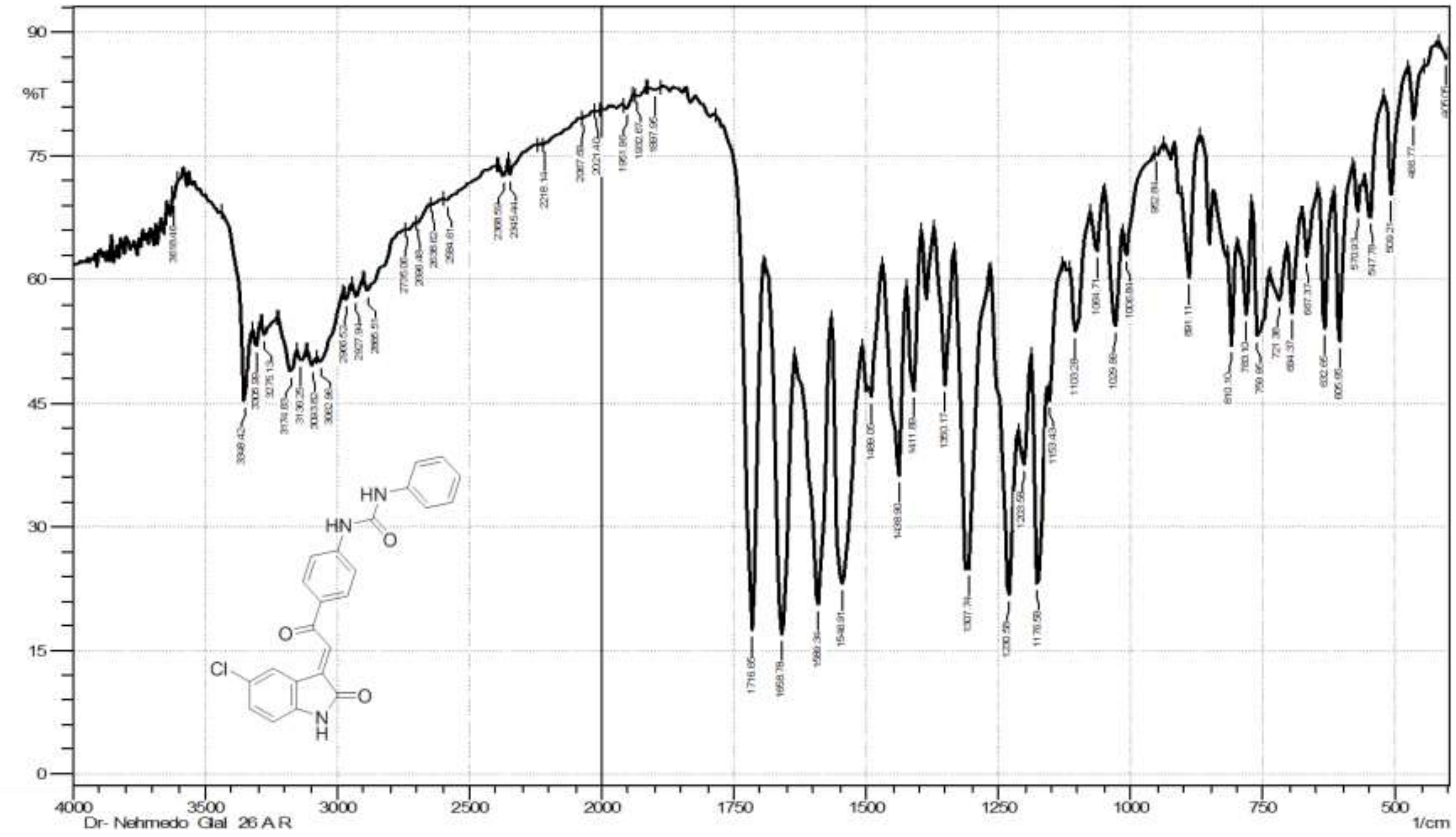

Fig. S46. IR spectrum of compound **12f** (KBr pellet).

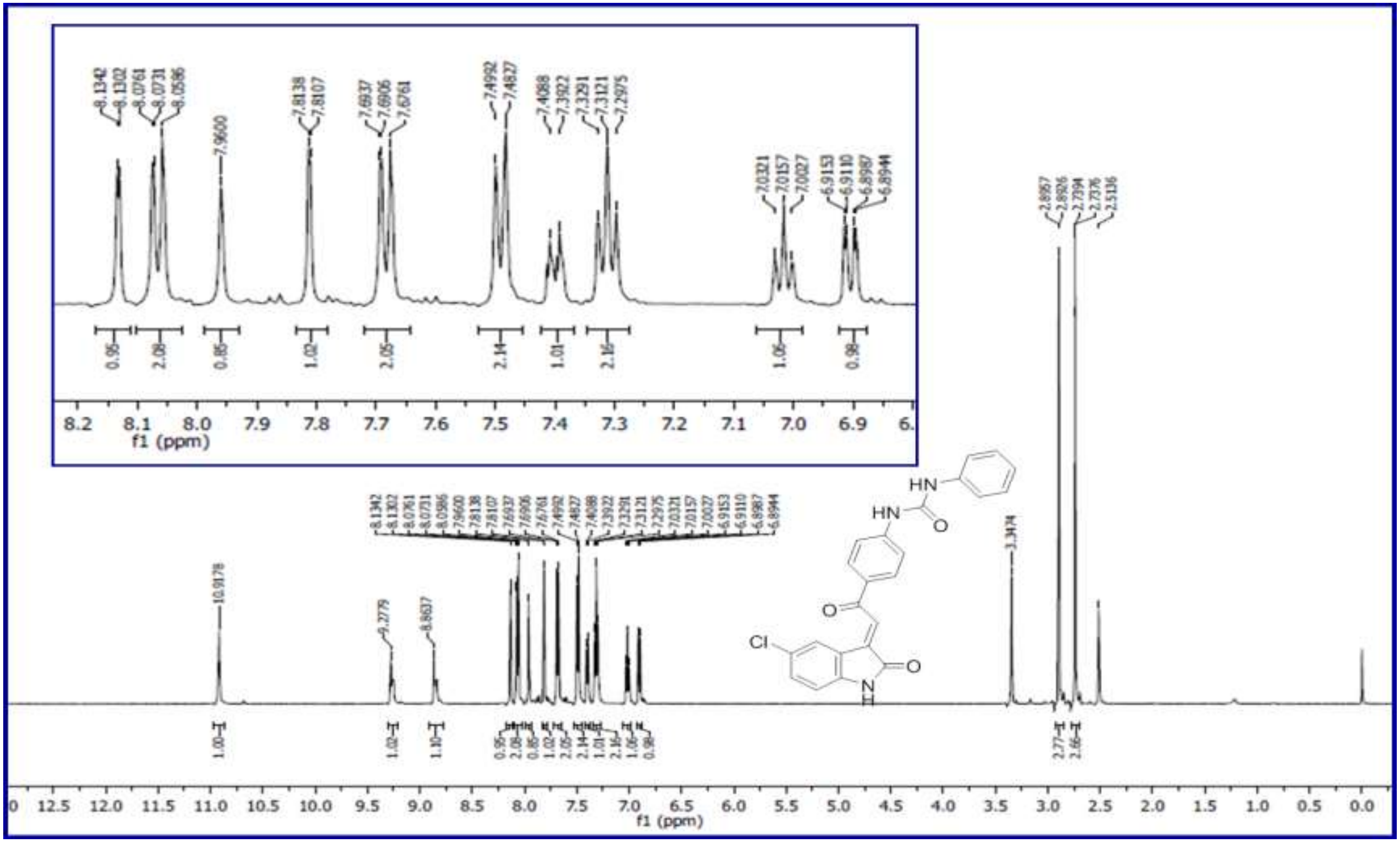

Fig. S47. ^{1}H-NMR spectrum of compound **12f** in DMSO-d_6 [singals at δ = 2.74, 2.89, 7.96 are for DMF, solvent of crystallization, Organometallics 29, 2176–2179 (2010)].

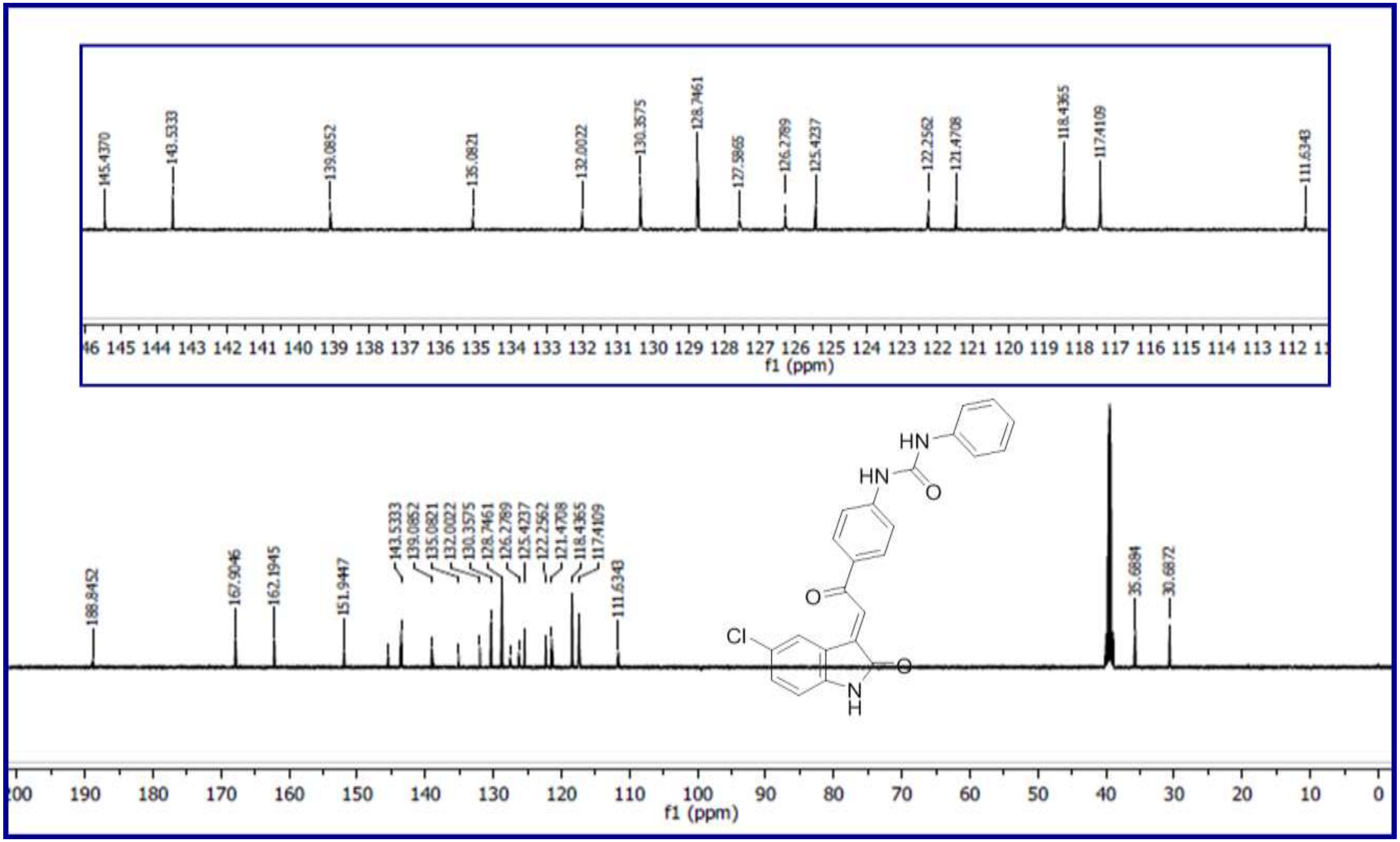

Fig. S48. ^{13}C-NMR spectrum of compound **12f** in DMSO-d_6 [singals at δ = 30.7, 35.7, 162.2 are for DMF, solvent of crystallization, Organometallics 29, 2176–2179 (2010)].

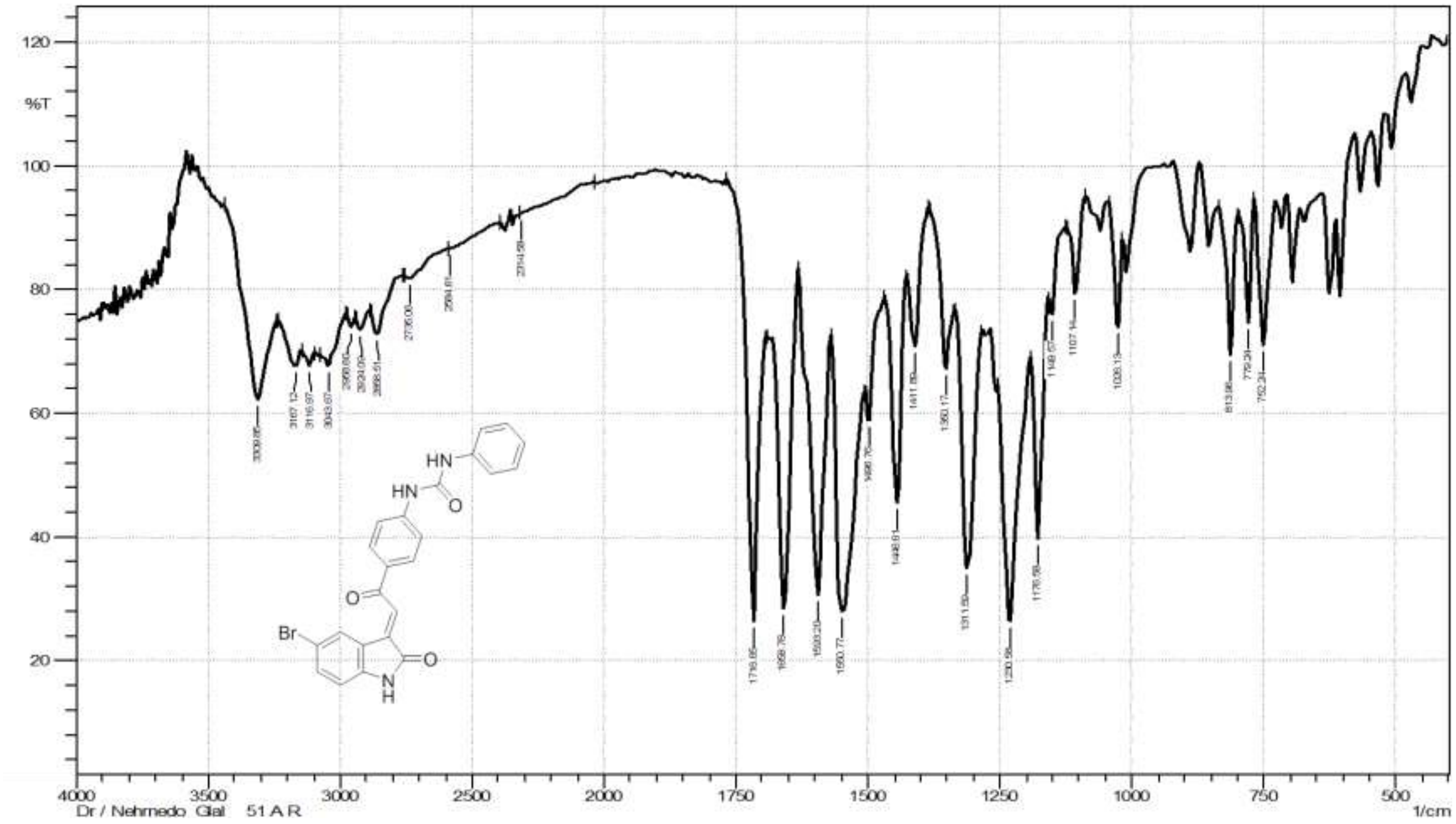

Fig. S49. IR spectrum of compound **12g** (KBr pellet).

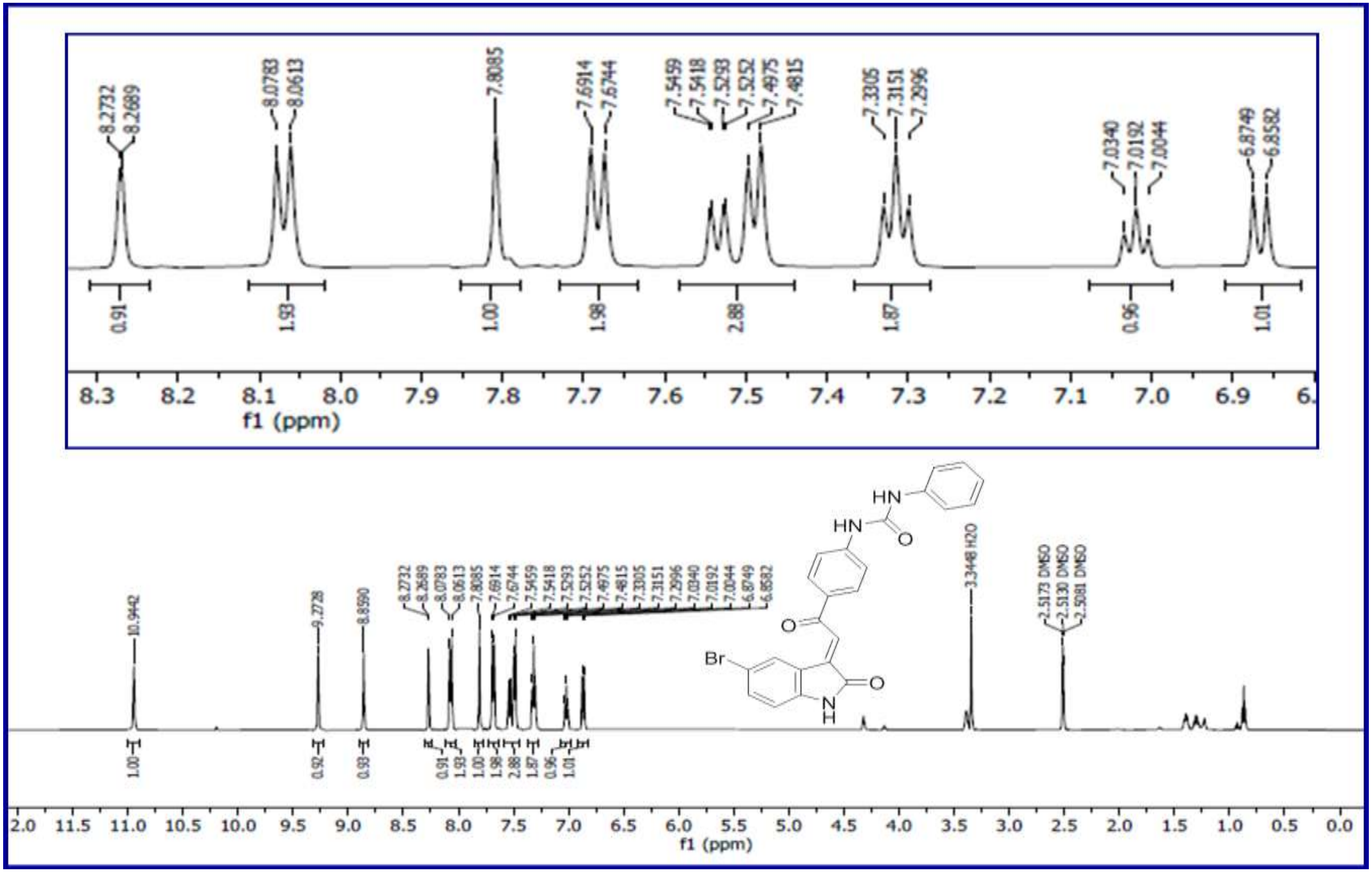

Fig. S50. ^{1}H-NMR spectrum of compound **12g** in DMSO-d_6.

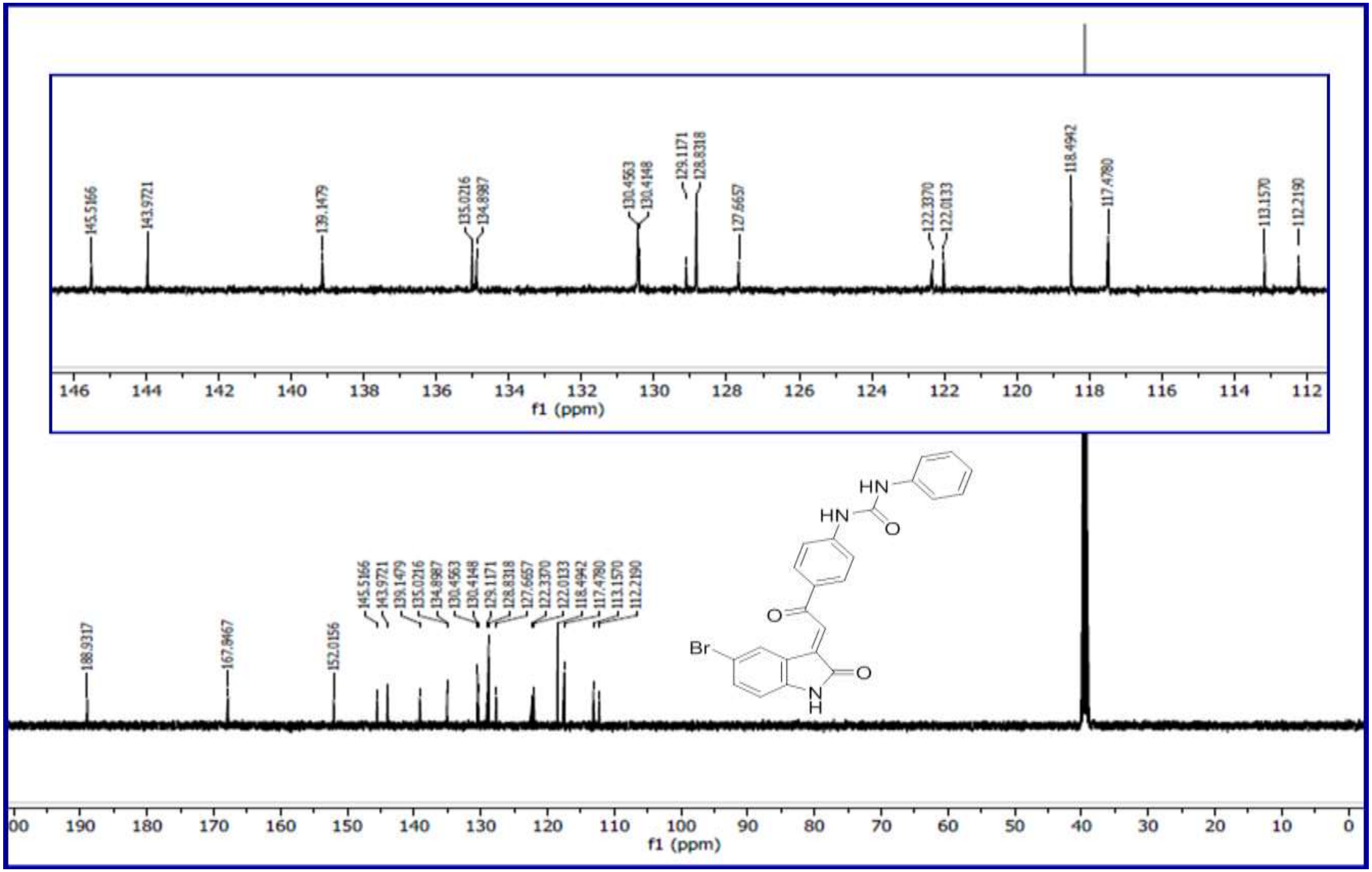

Fig. S51. ^{13}C-NMR spectrum of compound **12g** in DMSO-d_6.

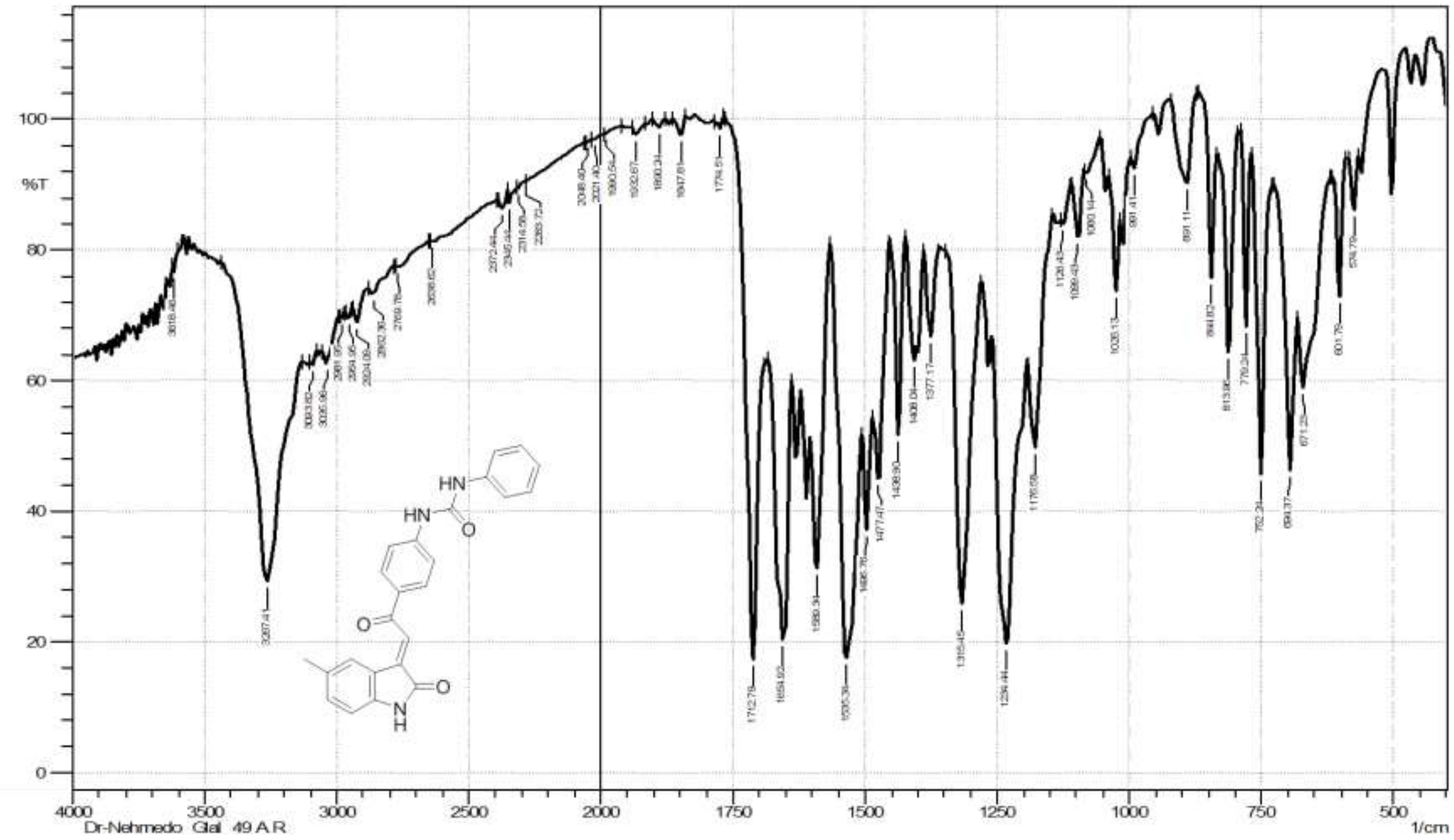

Fig. S52. IR spectrum of compound **12h** (KBr pellet).

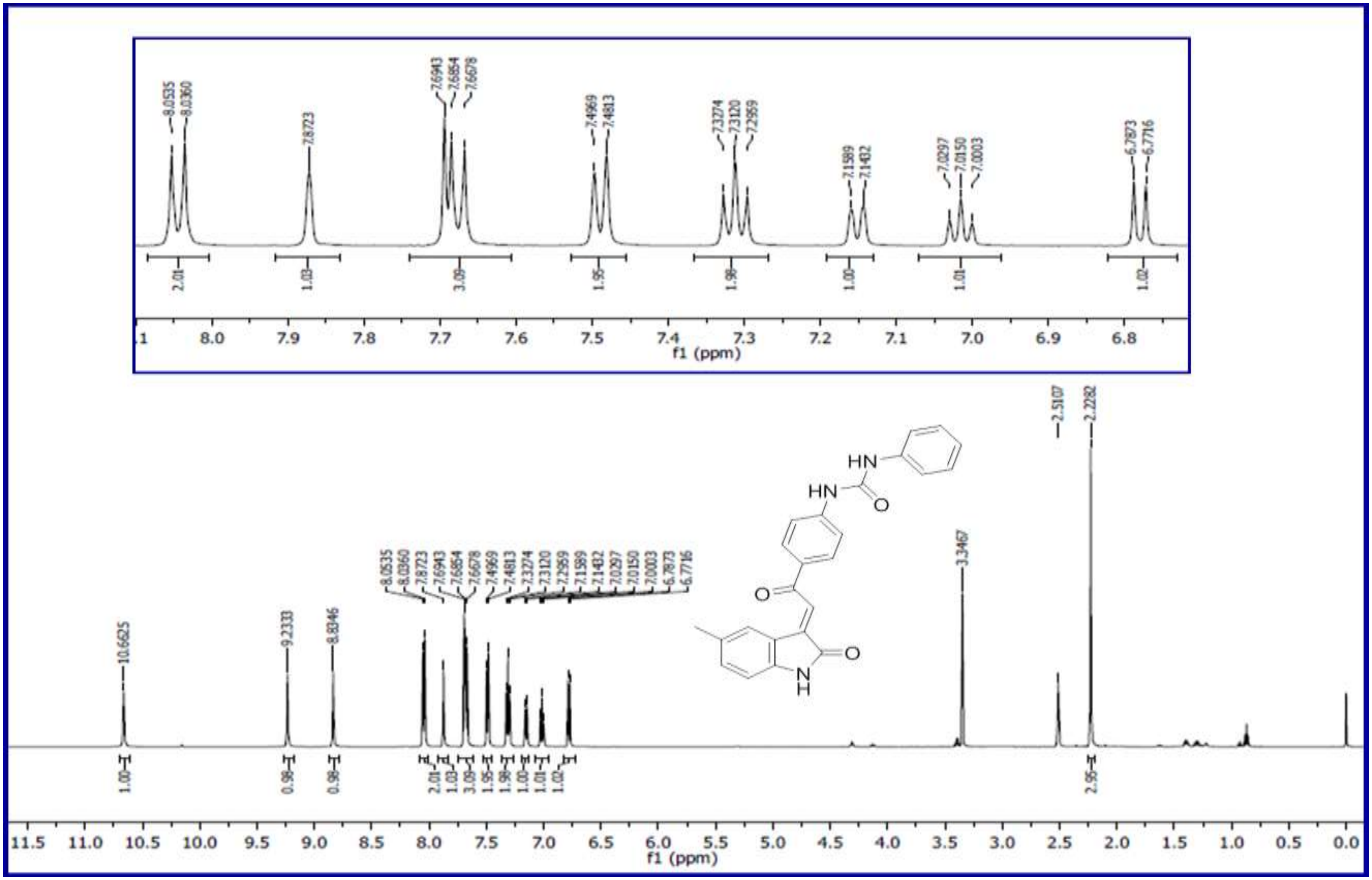

Fig. S53. ^{1}H-NMR spectrum of compound **12h** in DMSO-d_6.

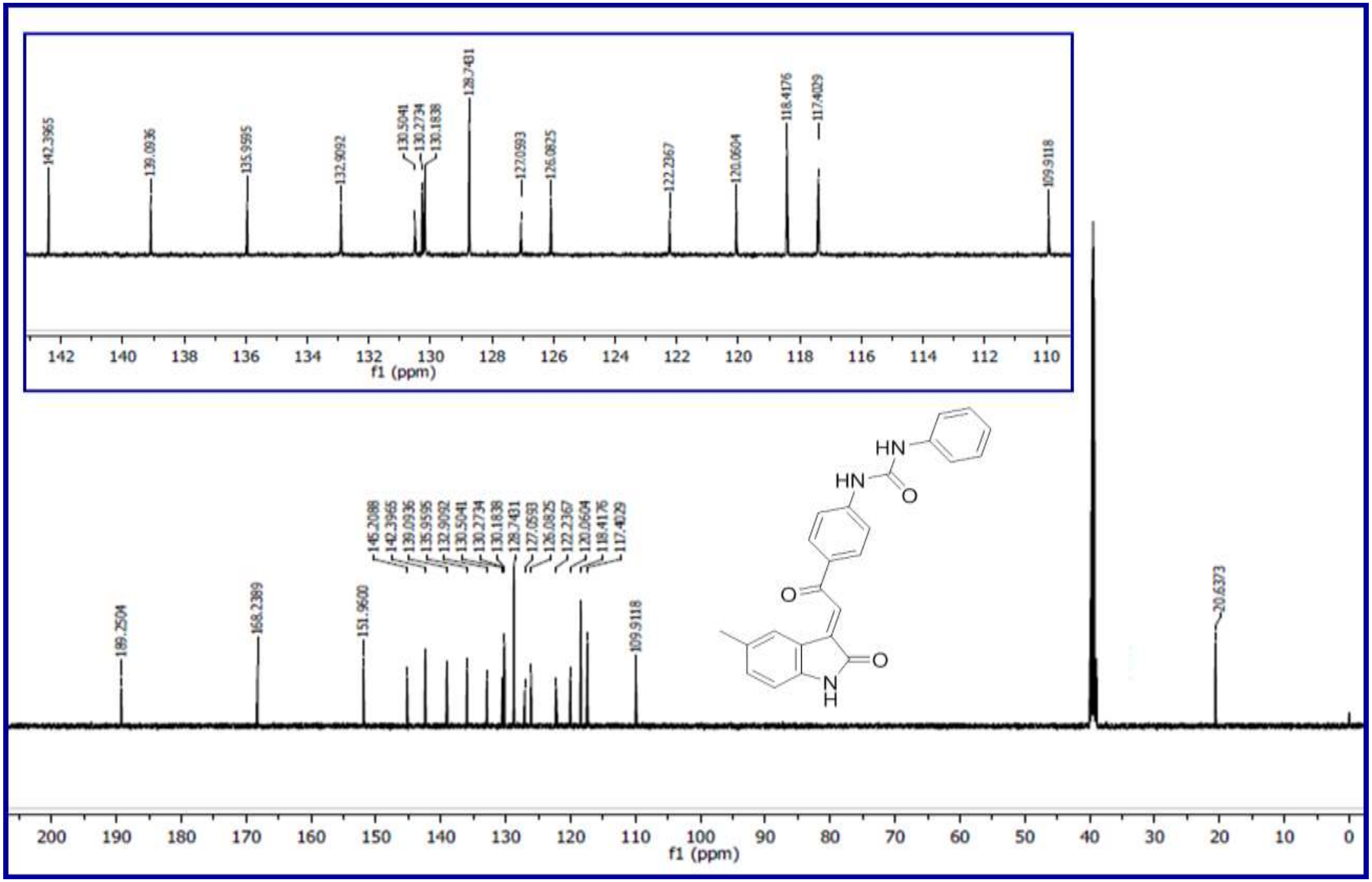

Fig. S54. ^{13}C-NMR spectrum of compound **12h** in DMSO-d_6.

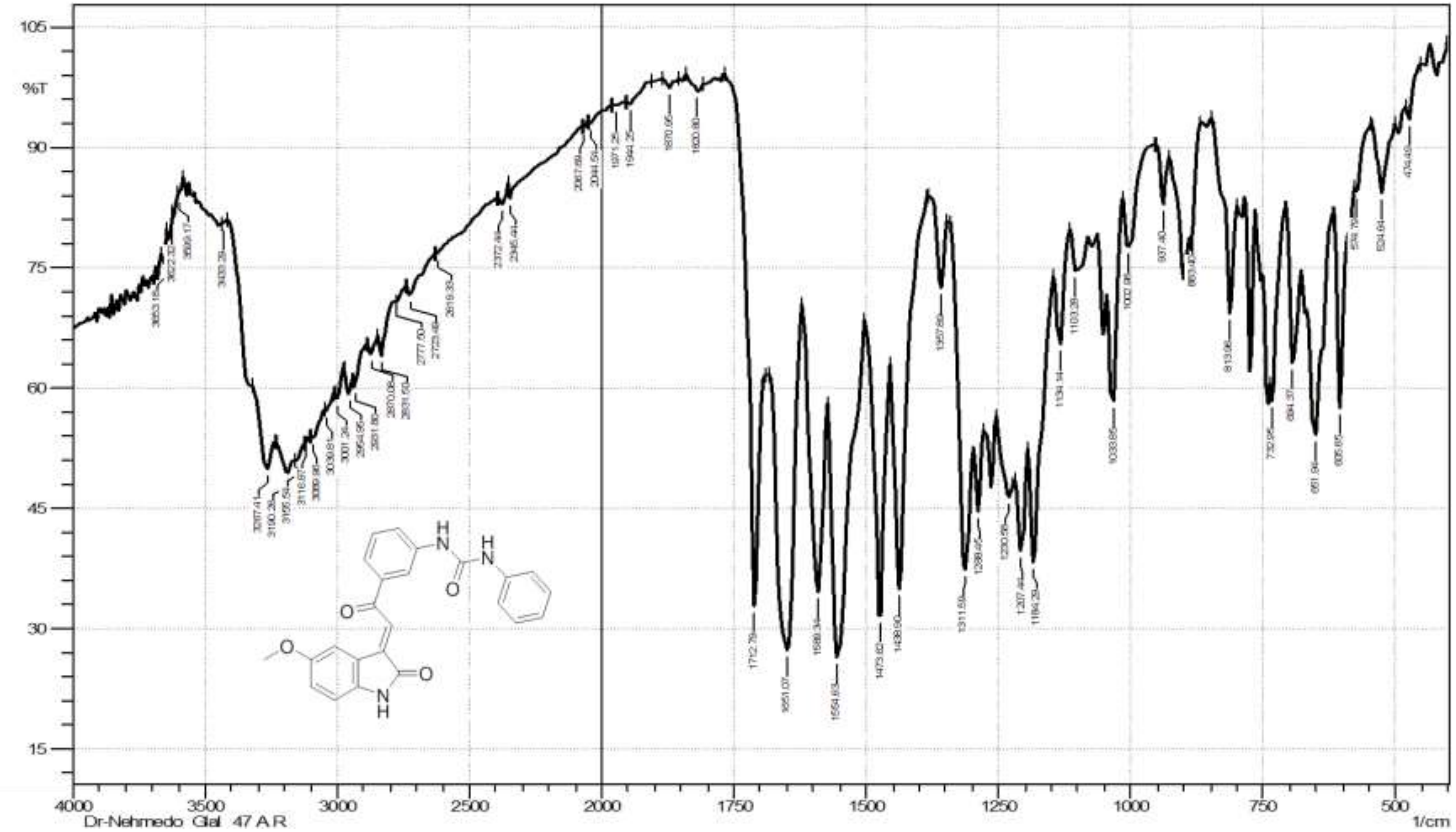

Fig. S55. IR spectrum of compound **12i** (KBr pellet).

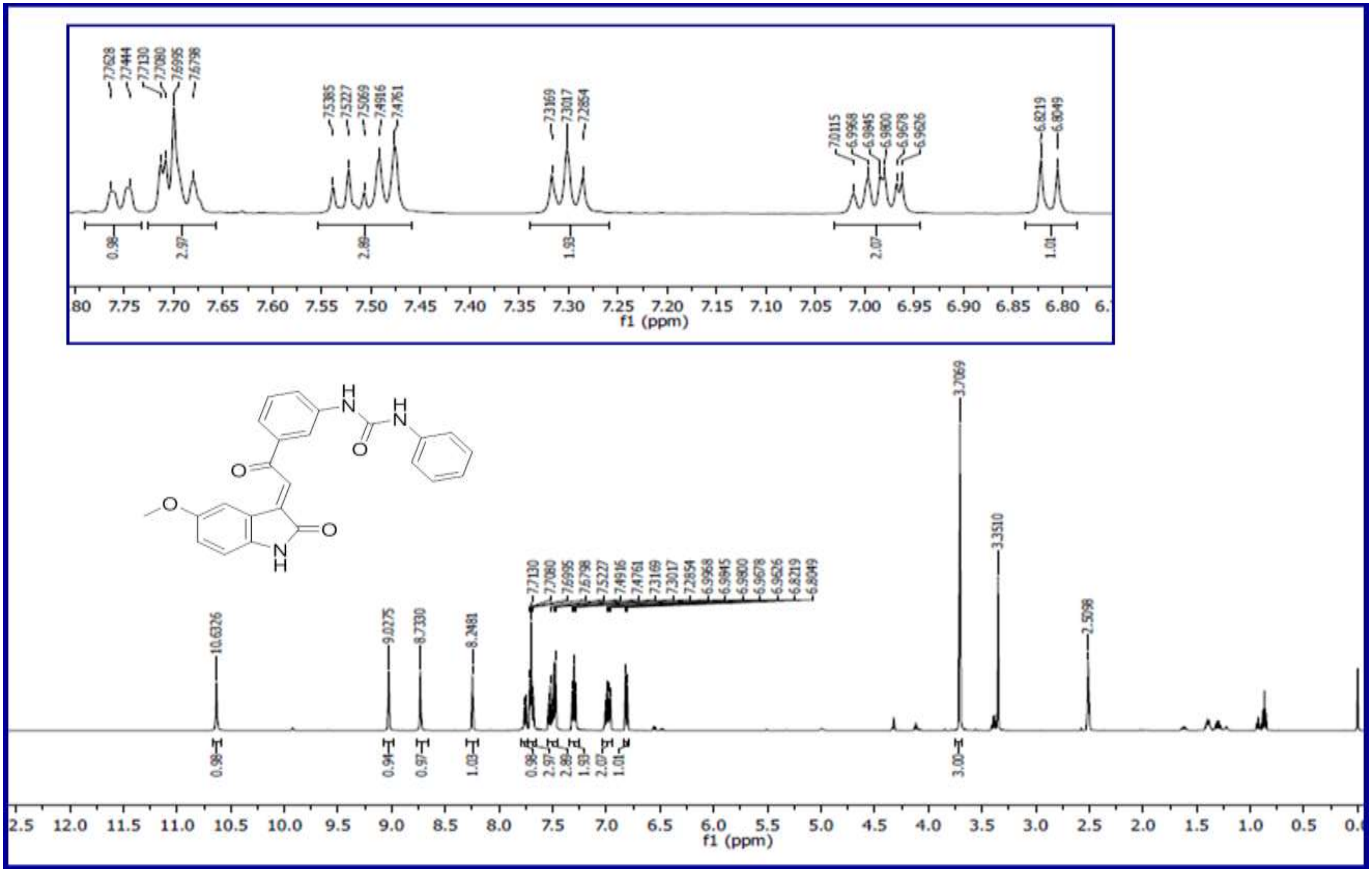

Fig. S56. ^{1}H-NMR spectrum of compound **12i** in DMSO-d_6.

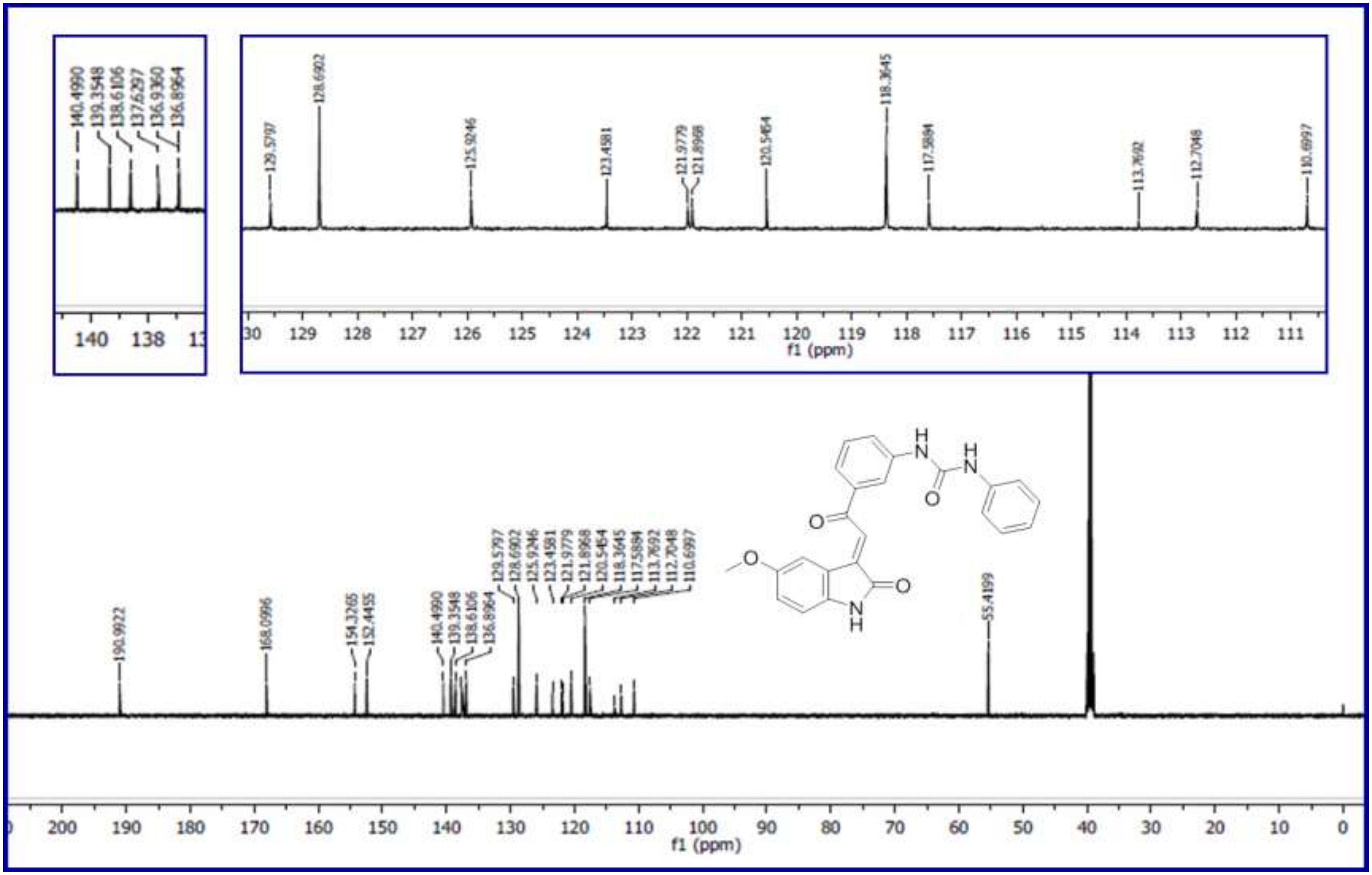

Fig. S57. ^{13}C-NMR spectrum of compound **12i** in DMSO-d_6.

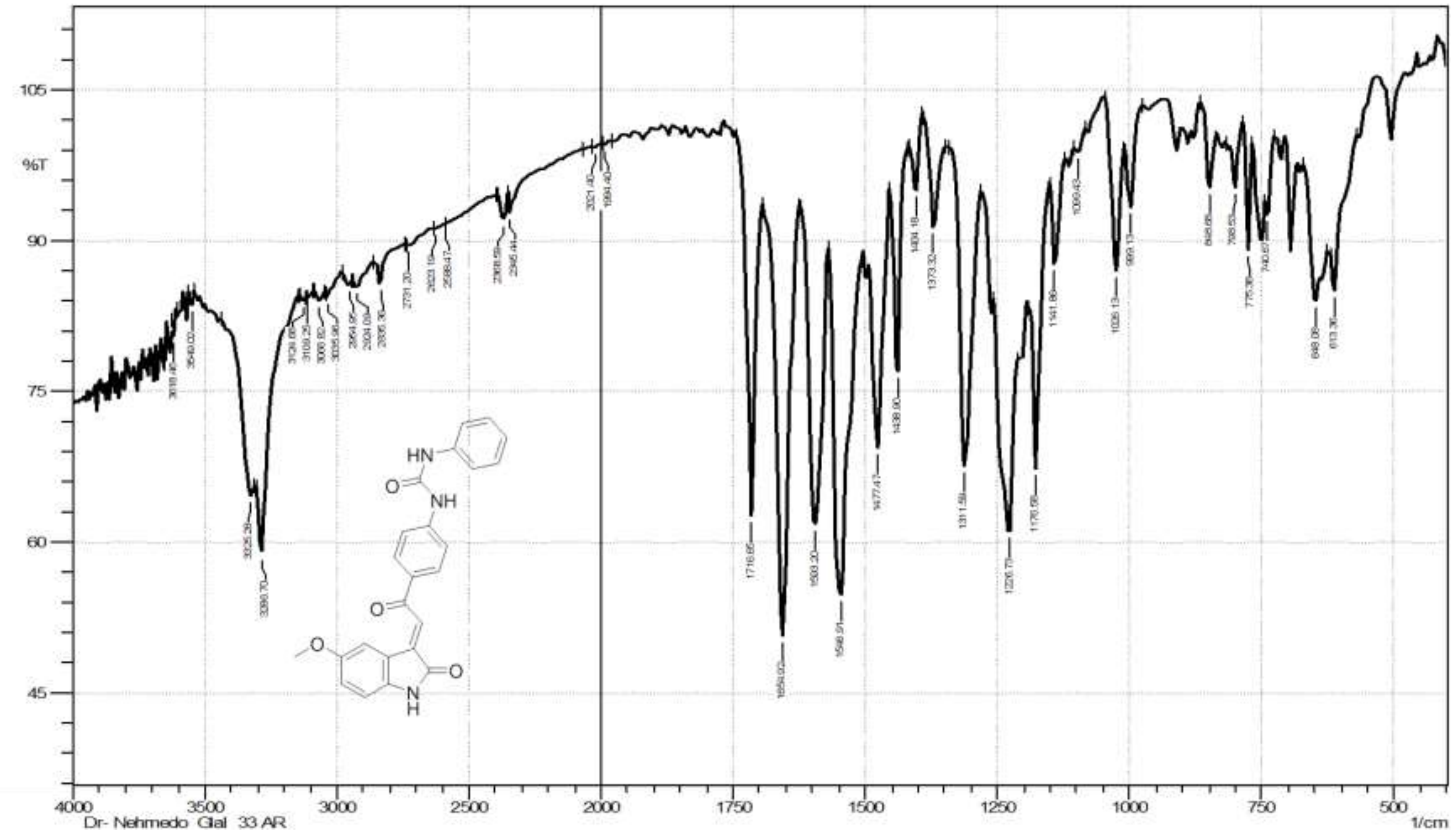

Fig. S58. IR spectrum of compound **12j** (KBr pellet).

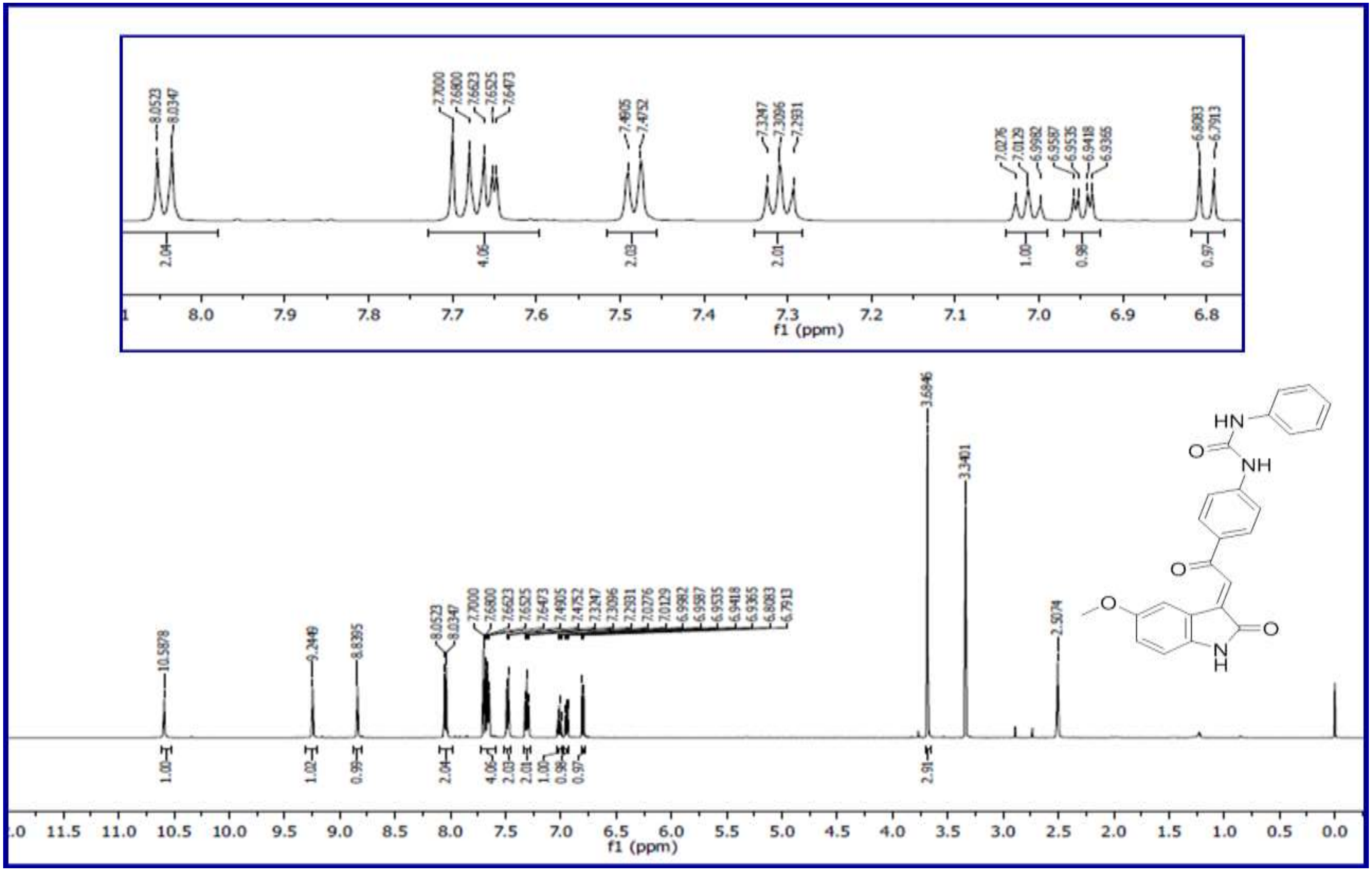

Fig. S59. ^{1}H-NMR spectrum of compound **12j** in DMSO-d_6.

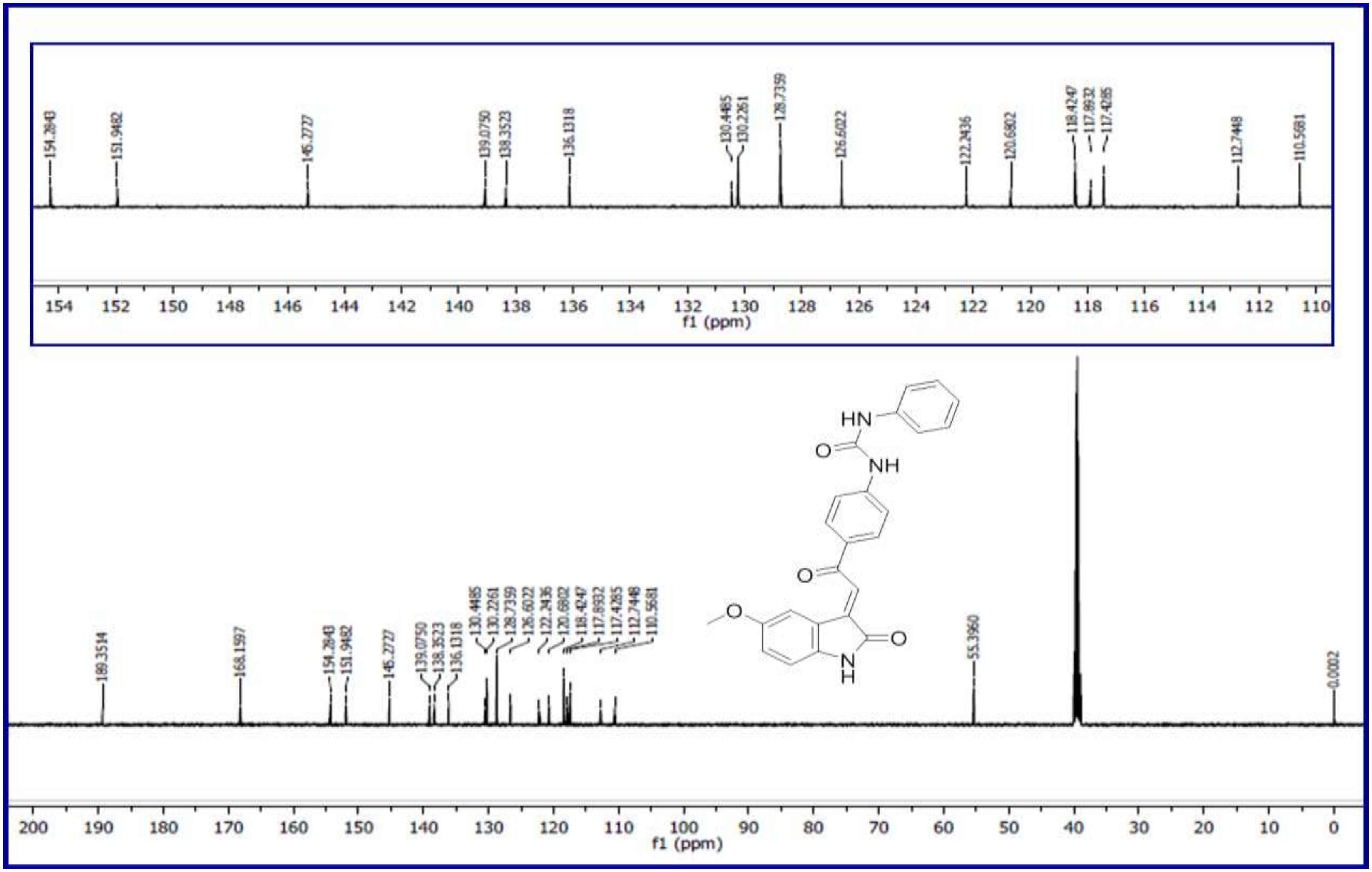

Fig. S60. ^{13}C-NMR spectrum of compound **12j** in DMSO-d_6.

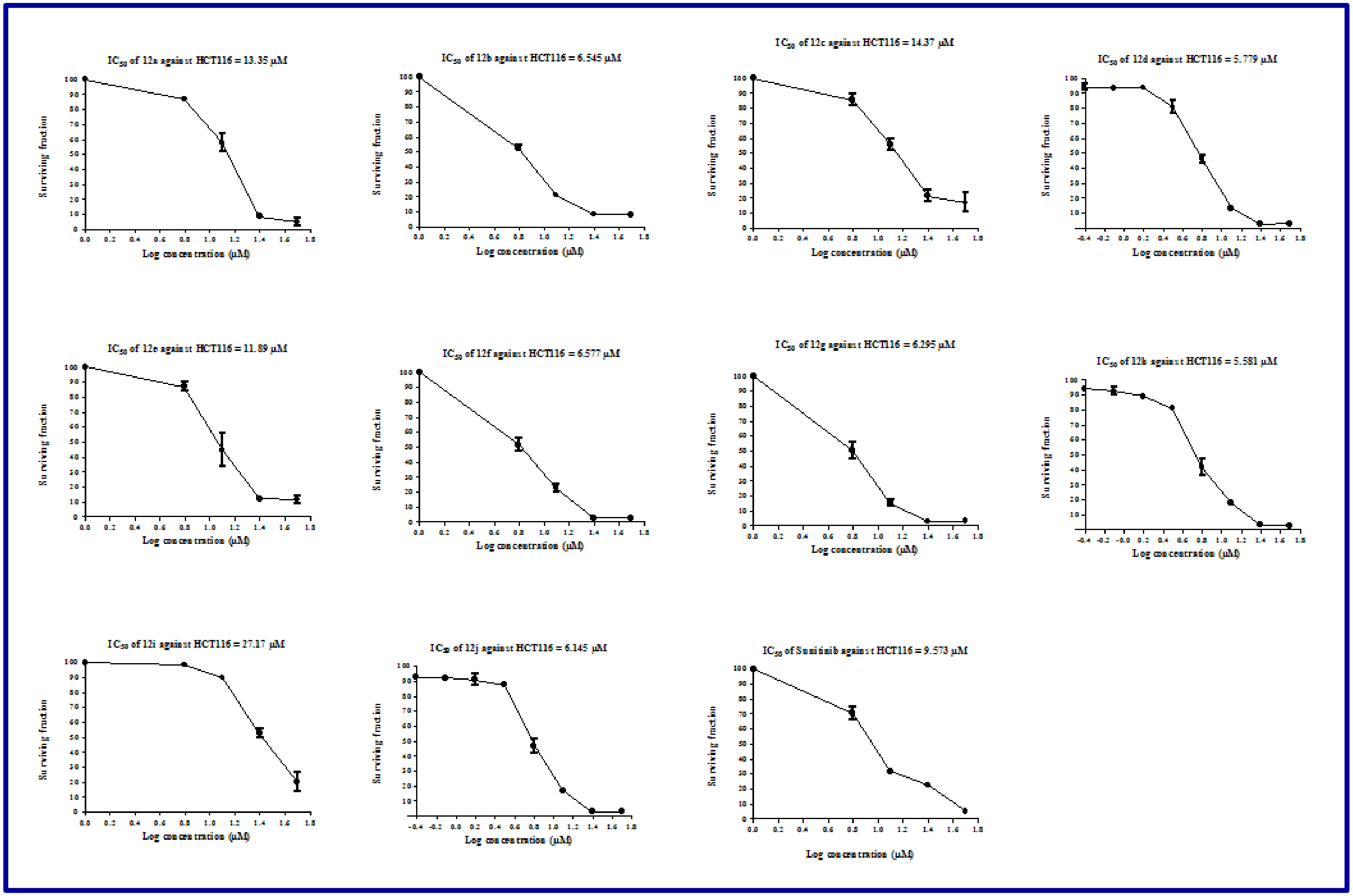

Fig. S61. Dose response curves of **12a–j** and sunitinib against HCT116 (colon) cancer cell line.

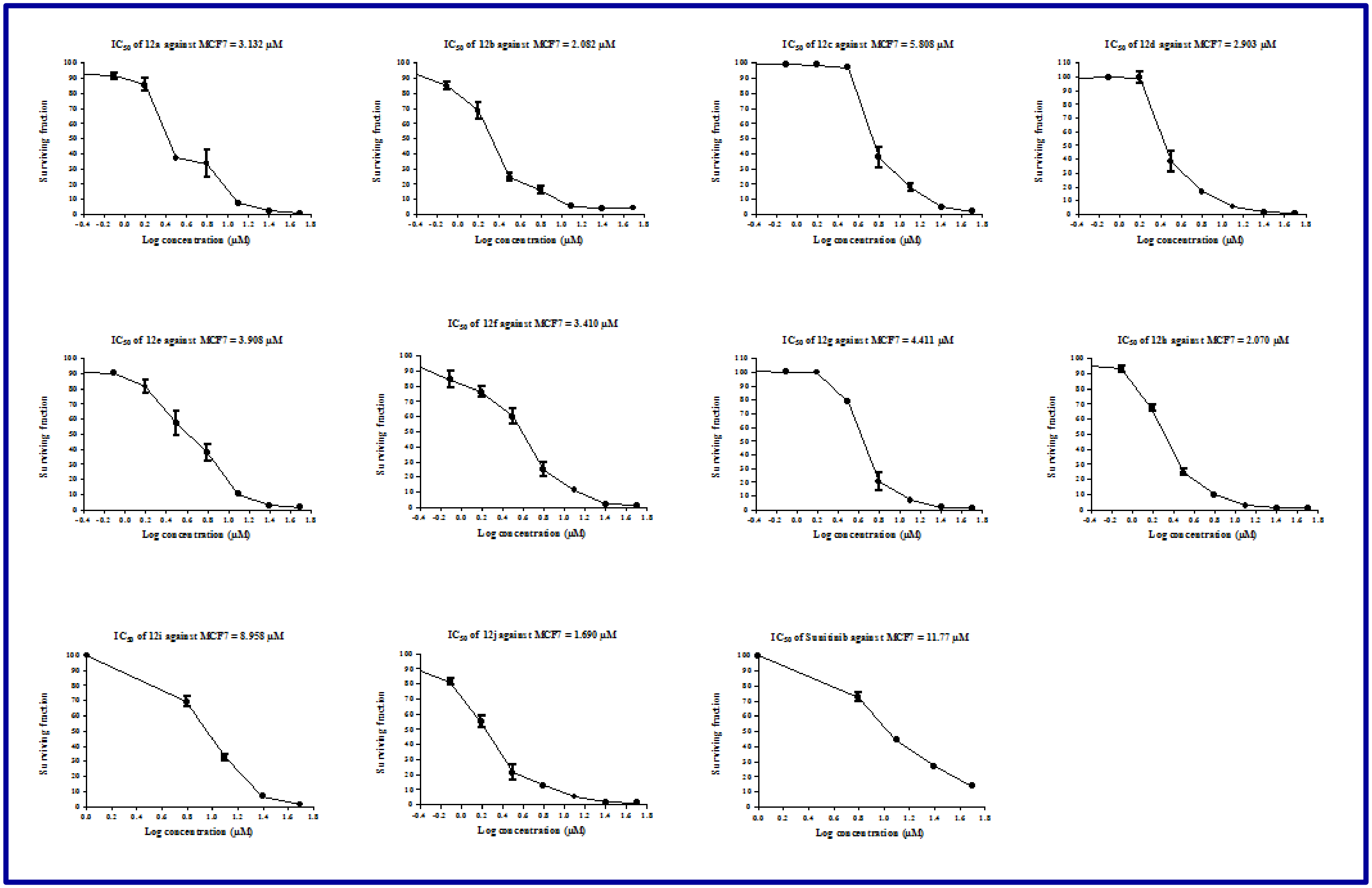

Fig. S62. Dose response curves of **12a–j** and sunitinib against MCF7 (breast) cancer cell line.

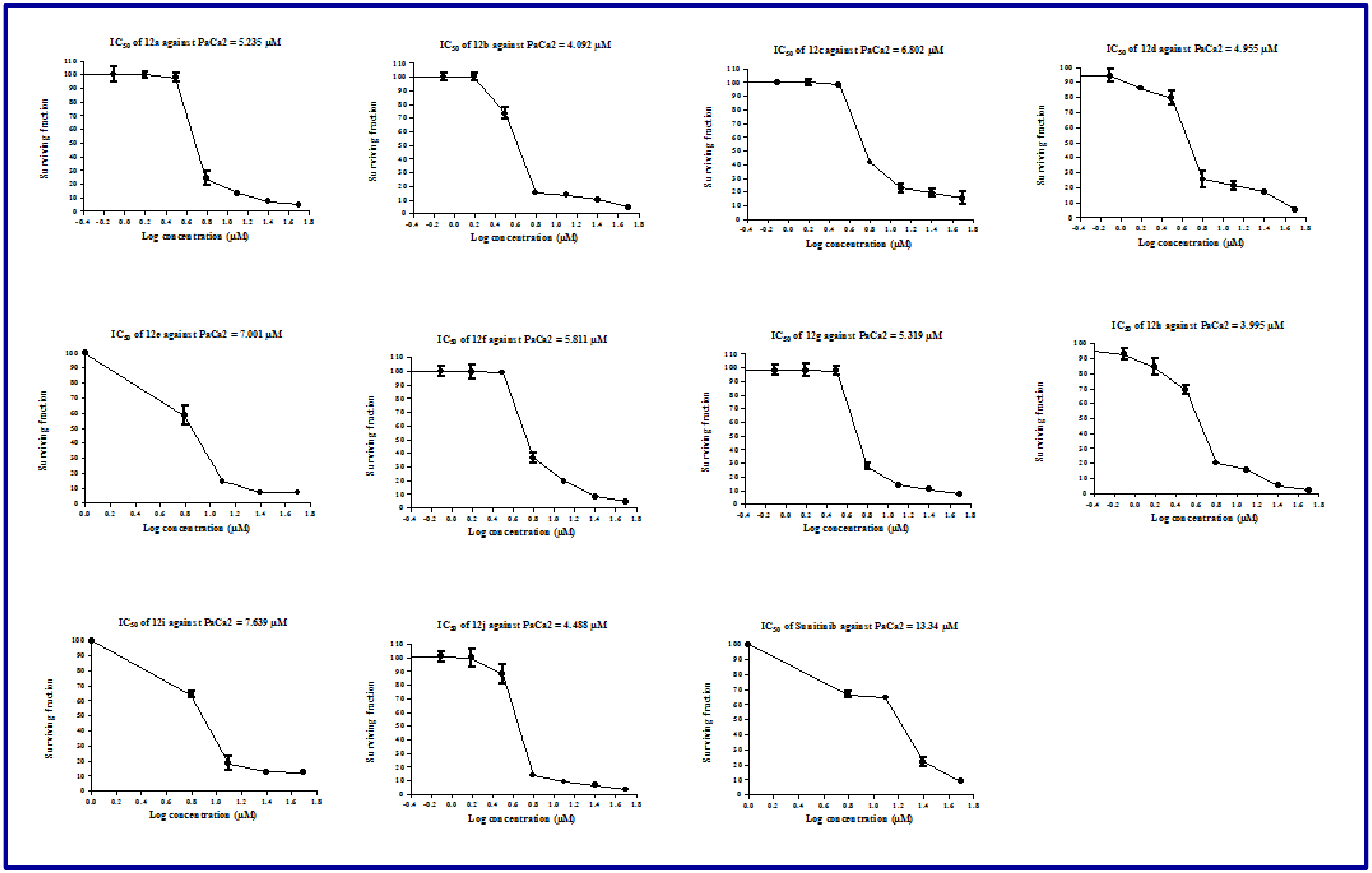

Fig. S63. Dose response curves of **12a–j** and sunitinib against PaCa2 (pancreatic) cancer cell line.

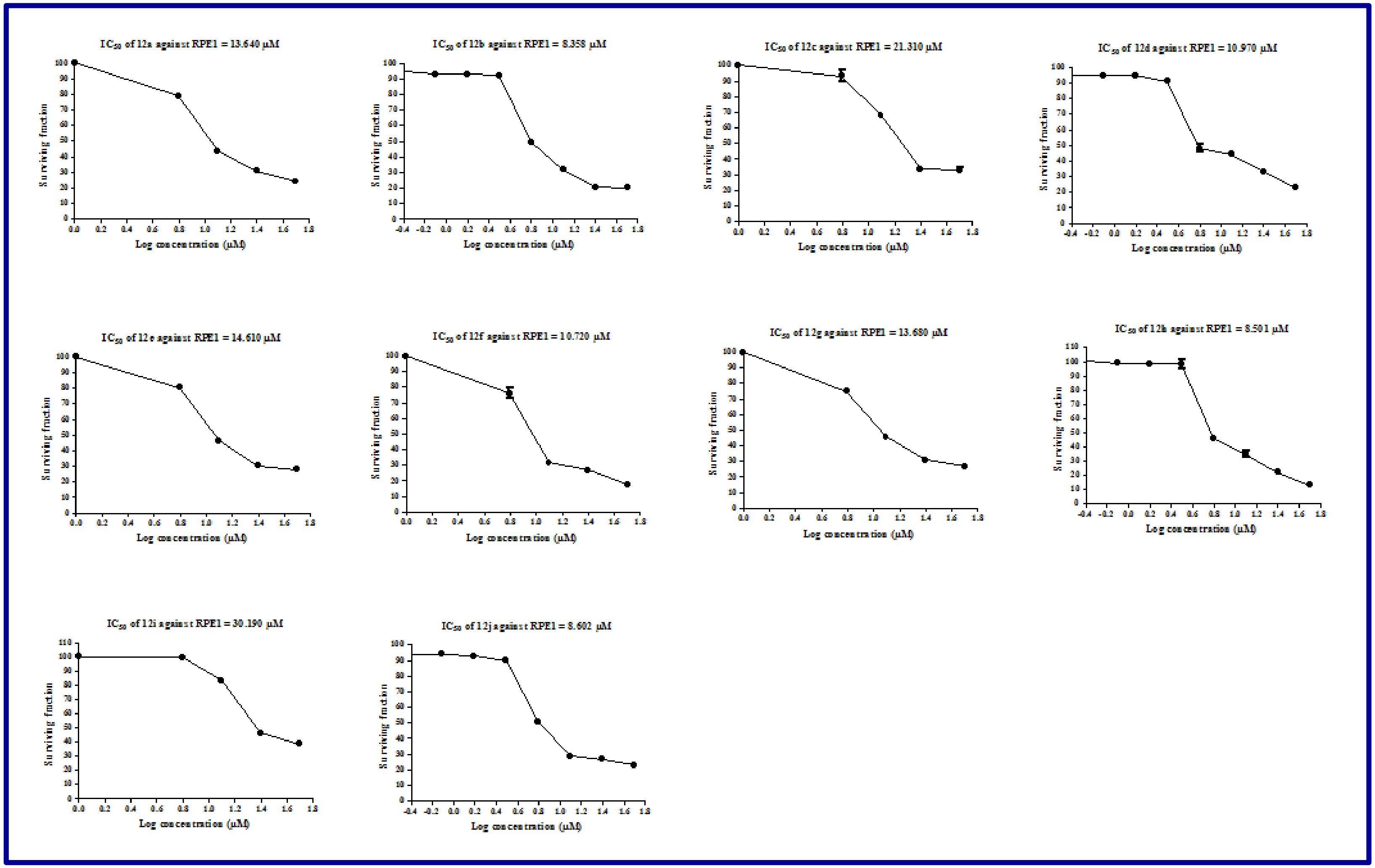

Fig. S64. Dose response curves of **12a–j** against RPE1 (pancreatic) cancer cell line.

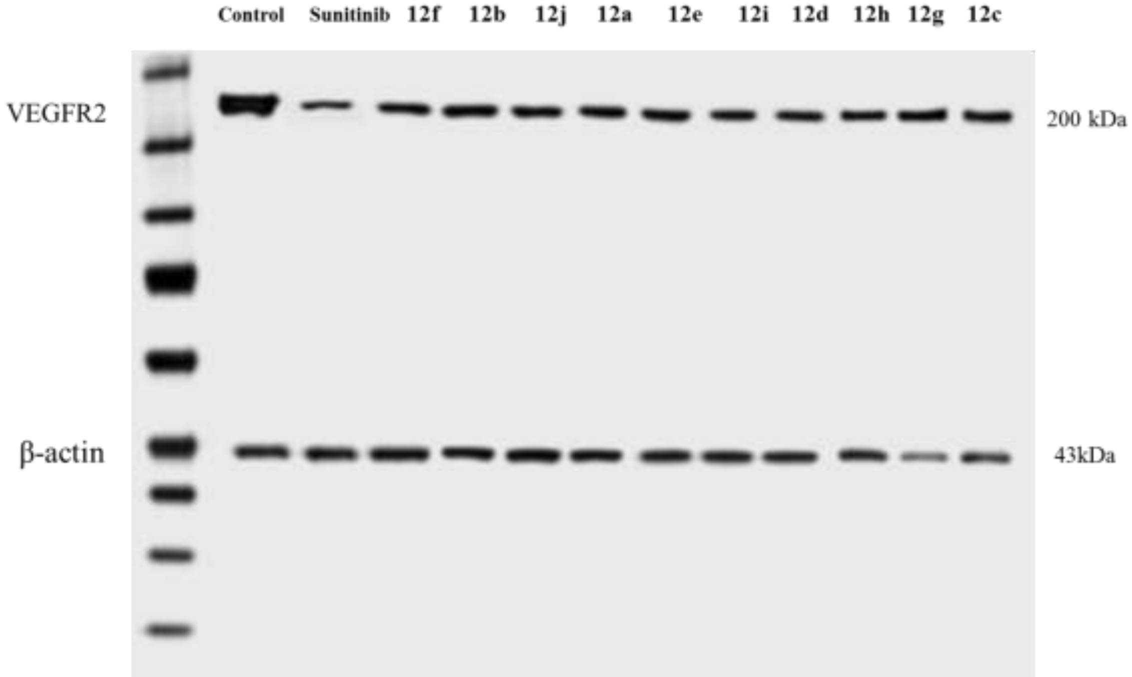

Fig. S65b. VEGFR-2 inhibitory properties of **12a–j** and sunitinib utilizing Western Blotting technique (uncropped).

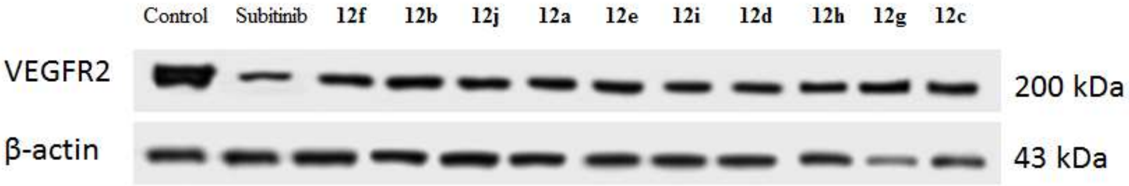

Fig. S65b. VEGFR-2 inhibitory properties of **12a–j** and sunitinib utilizing Western Blotting technique.

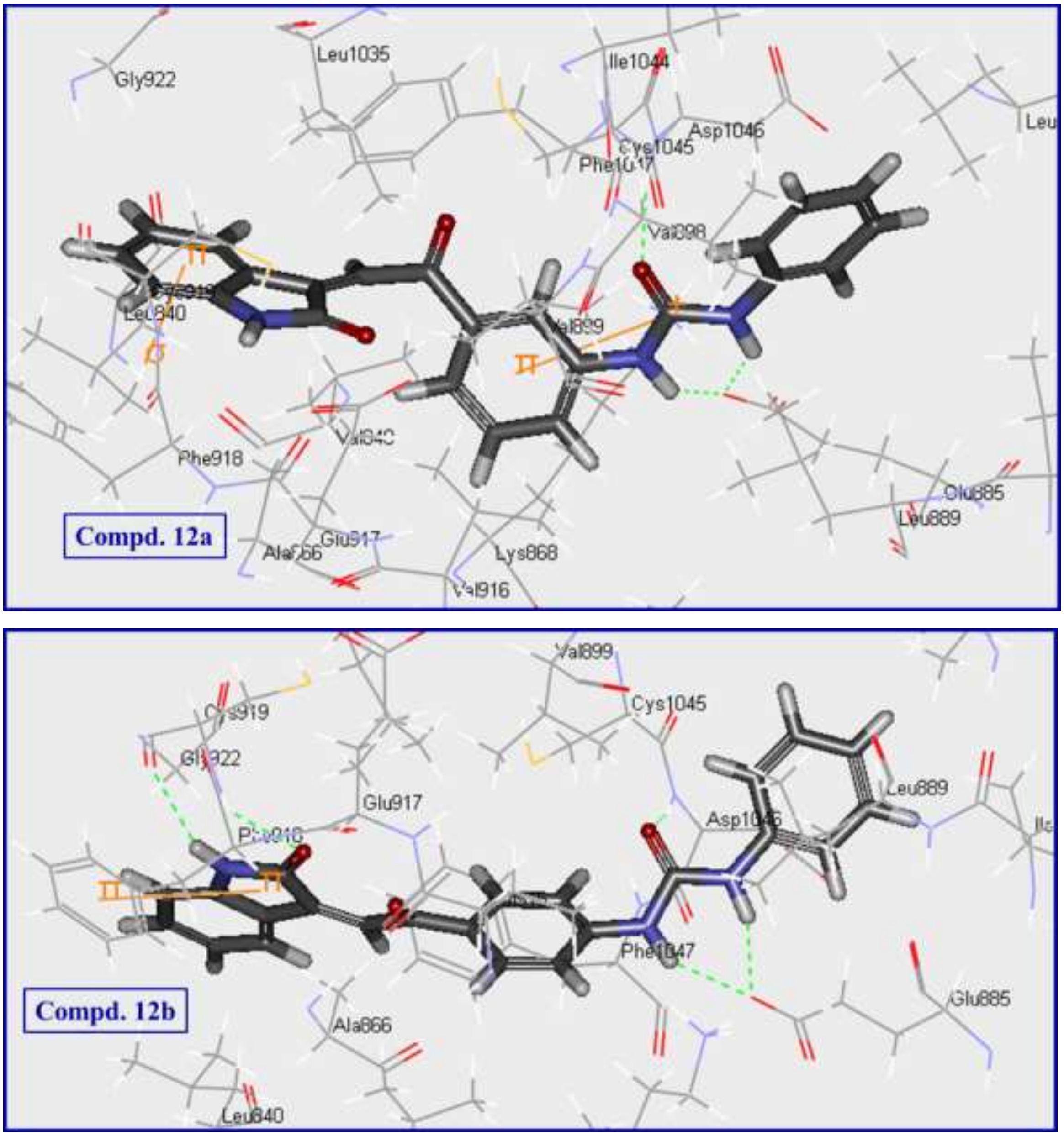
Gly922
Leu1035
Ile1044
Asp1046
Cys1045
Phe1047
Val898
Leu840
Val899
Val848
Phe918
Glu885
Leu889
Compd. 12a
Ala866
Glu917
Lys868
Val916
Val899
Cys1045
Cys919
Gly922
Leu889
Glu917
Phe918
Asp1046
Phe1047
Glu885
Compd. 12b
Ala866
Leu840

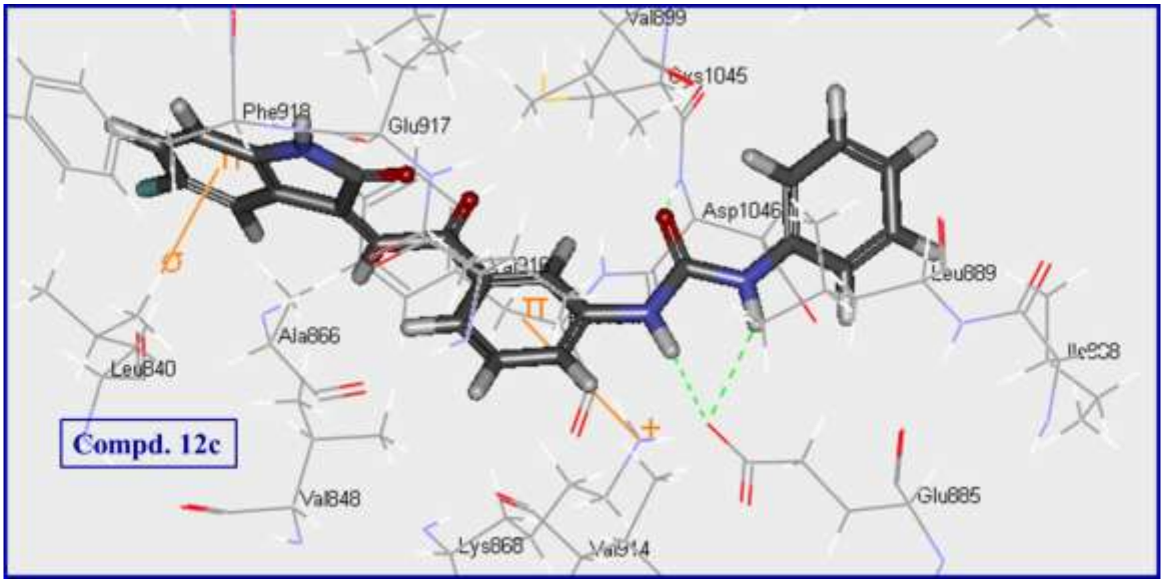

Val899
Cys1045
Phe918
Glu917
Asp1046
Leu889
Ala866
Ile888
Leu840
Compd. 12c
Val848
Glu885
Lys868
Val914

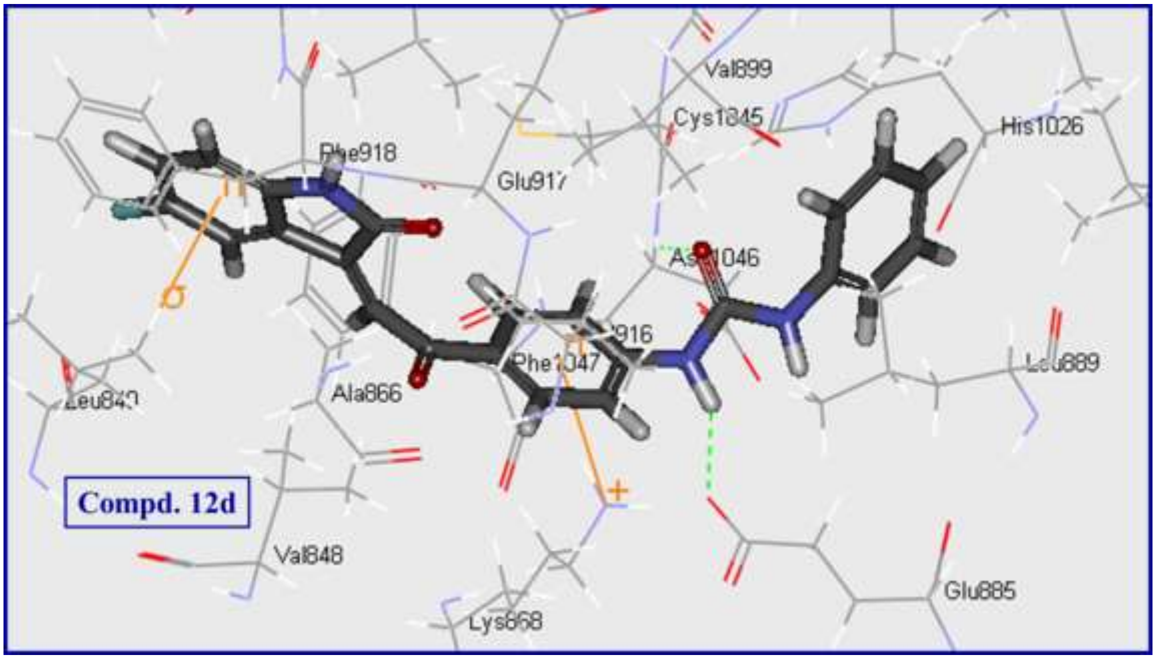

Val899
Cys1045
His1026
Phe918
Glu917
Phe1047
Leu889
Ala866
Compd. 12d
Val848
Glu885
Lys868

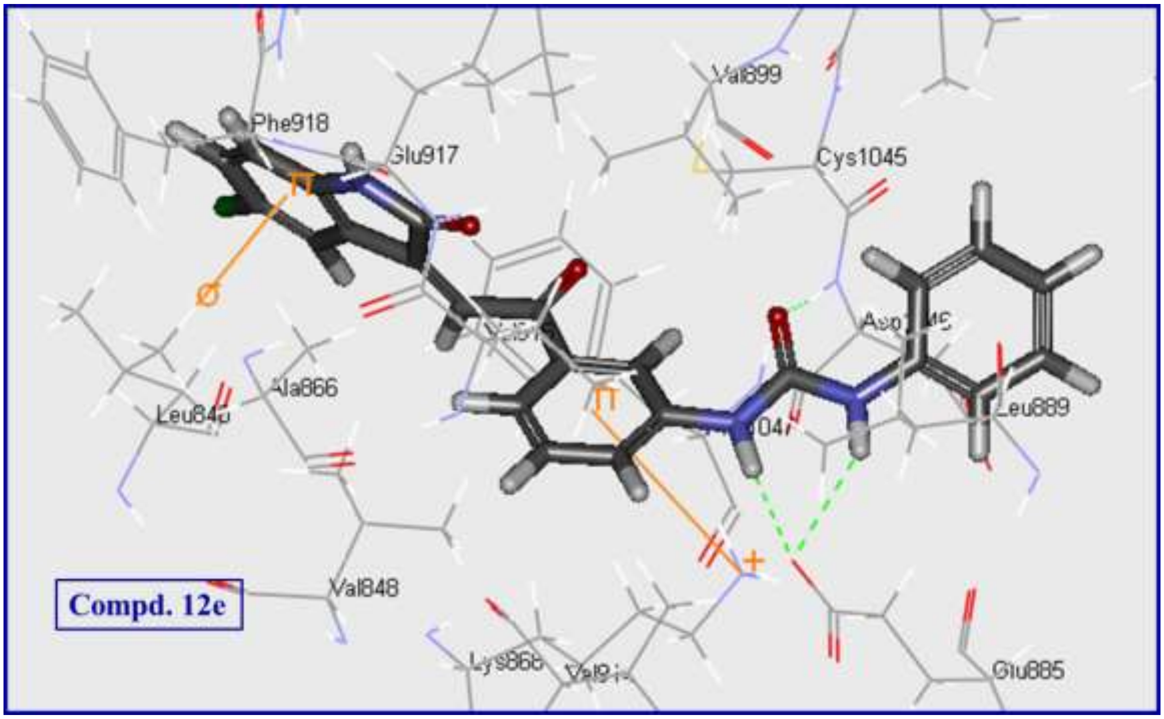
Val899
Phe918
Glu917
Cys1045
Ala866
Leu889
Val848
Glu885
Compd. 12e

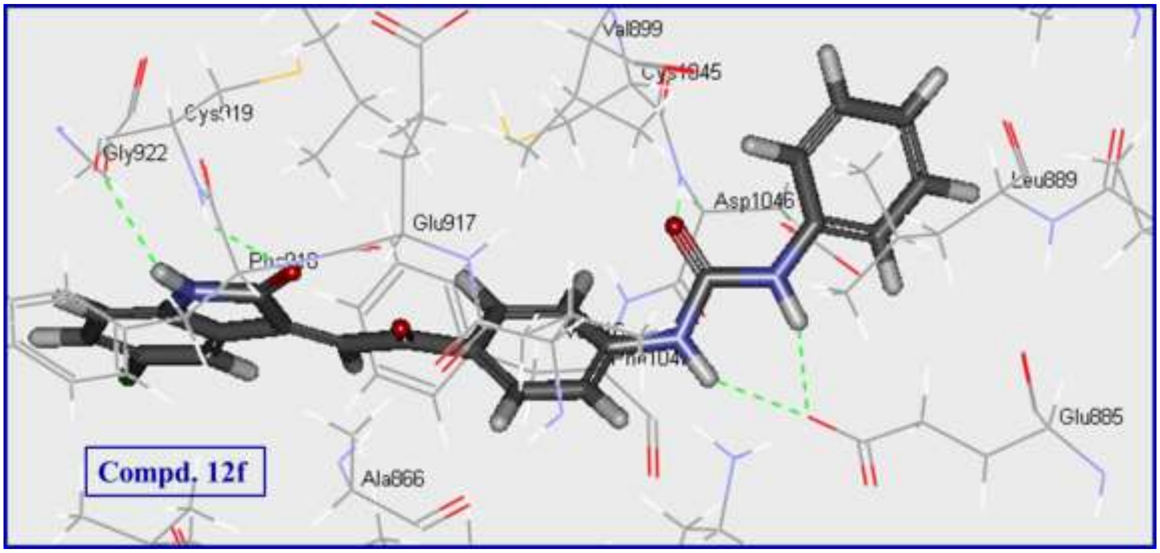
Val899
Gly922
Glu917
Asp1046
Leu889
Glu885
Ala866
Compd. 12f

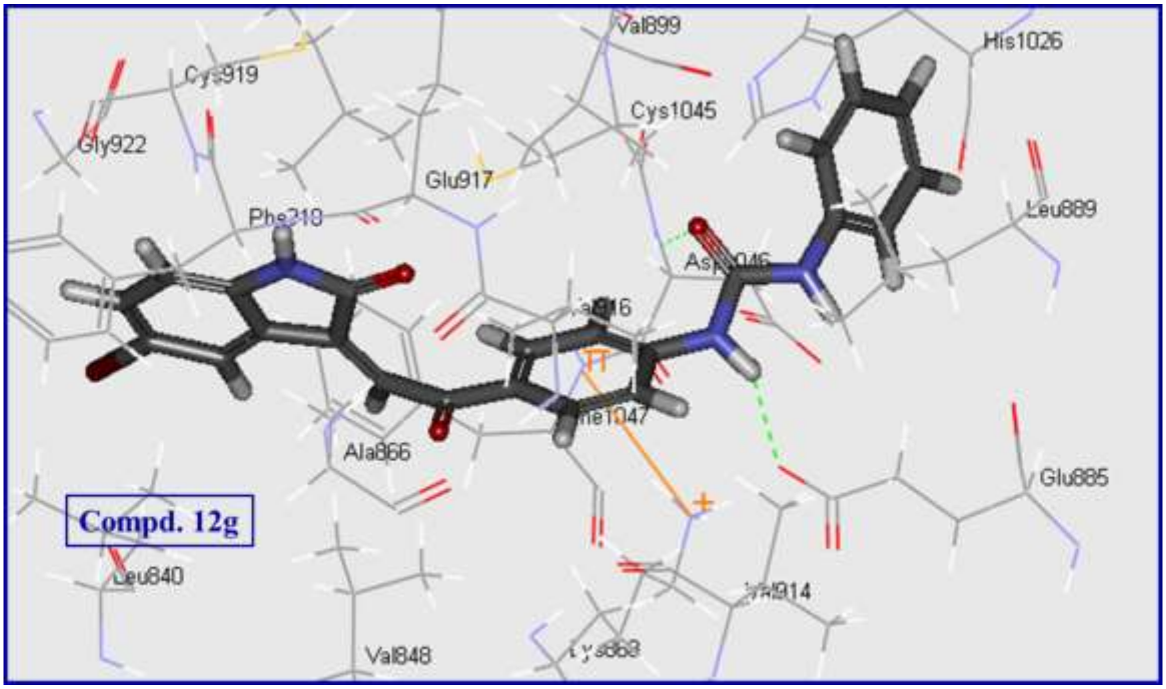
Val899
His1026
Cys919
Cys1045
Gly922
Glu917
Leu889
Phe918
Asp1046
Val916
Phe1047
Ala866
Glu885
Compd. 12g
Leu840
Val914
Val848
Lys868

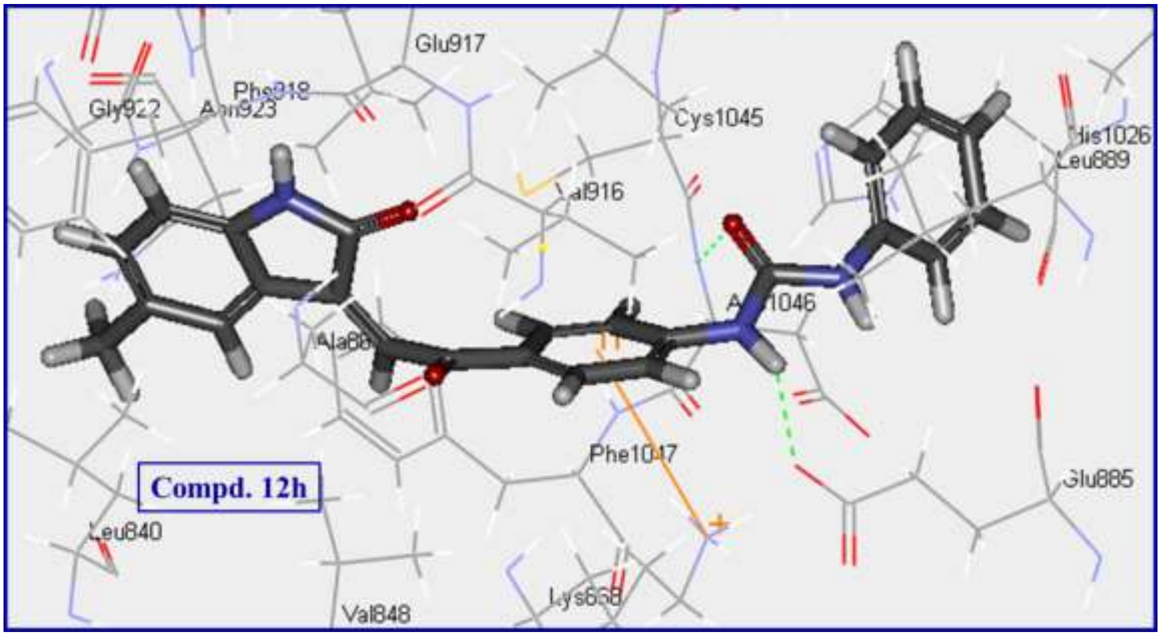
Glu917
Phe918
Gly922
Cys1045
His1026
Leu889
Val916
Phe1047
Glu885
Compd. 12h
Leu840
Lys868
Val848

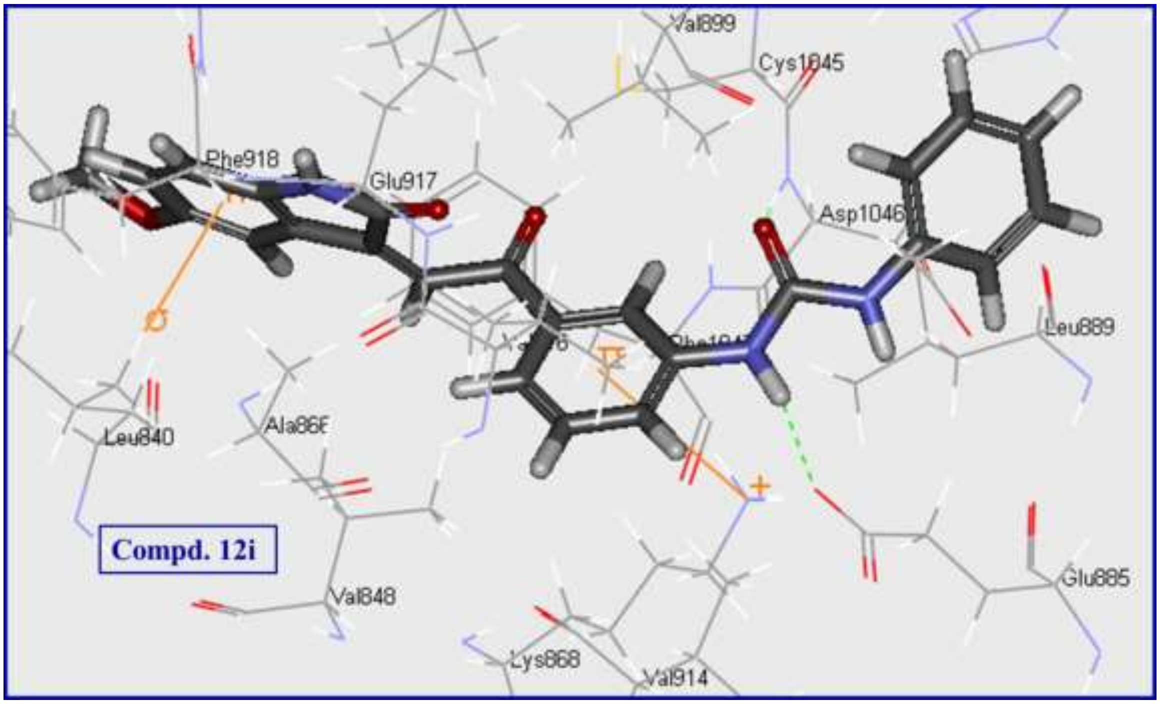
Val899
Cys1045
Phe918
Glu917
Asp1046
Leu889
Leu840
Ala866
Compd. 12i
Val848
Glu885
Lys868
Val914

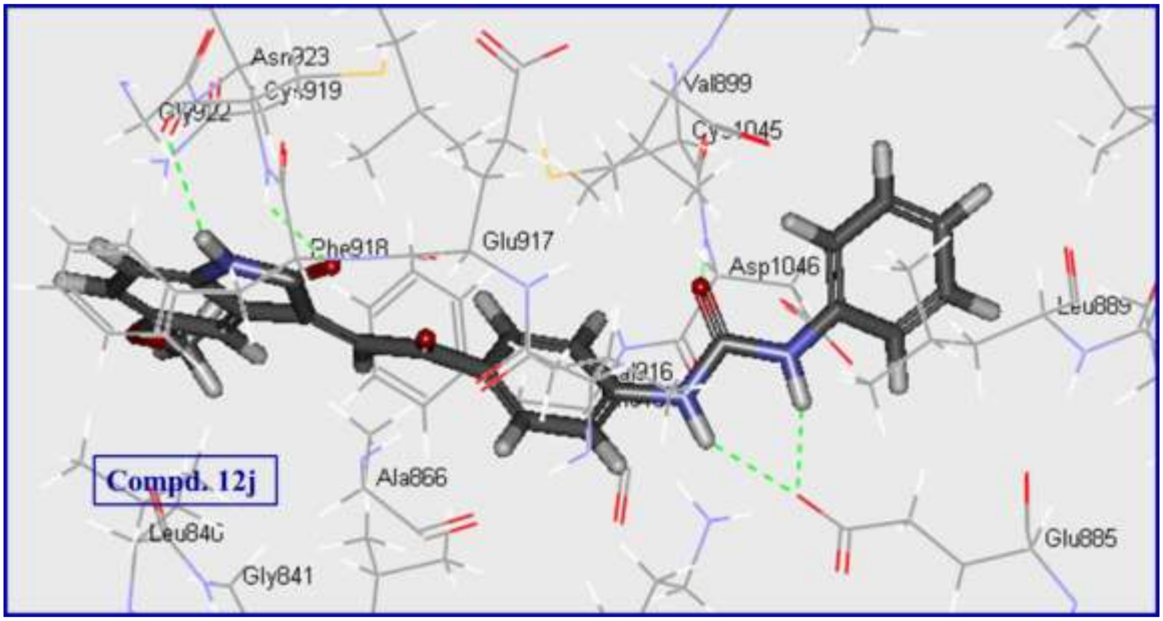
Asn923
Cys919
Val899
Phe918
Glu917
Asp1046
Leu889
Compd. 12j
Ala866
Glu885
Gly841

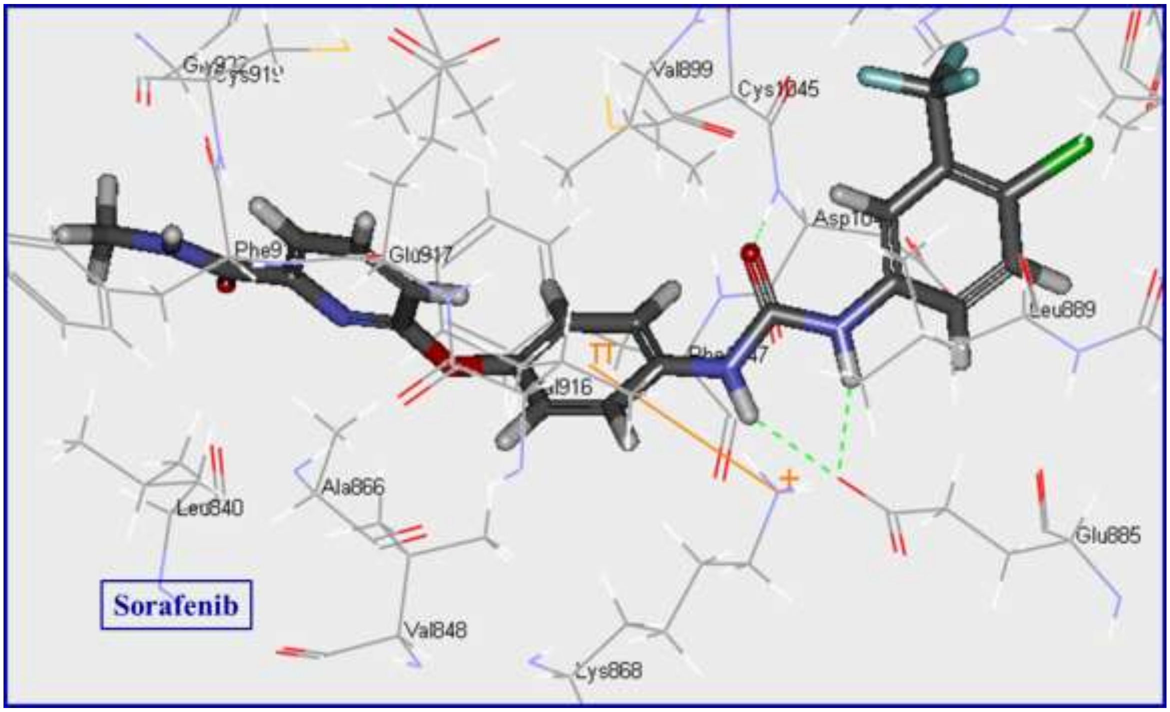

Fig. S66. Docking poses of the **12a–j** and sorafenib (co-crystallized ligand) in PDB ID: 3WZE.

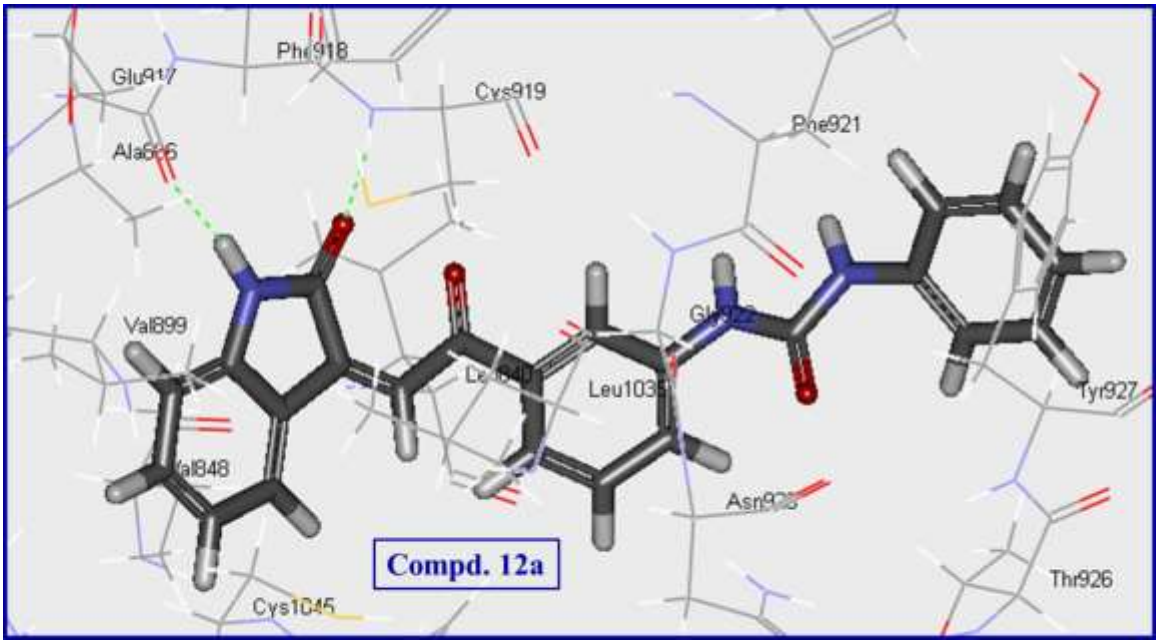
Compd. 12a
Glu917
Ala866
Cys919
Phe921
Val899
Leu1035
Tyr927
Thr926
Cys1045

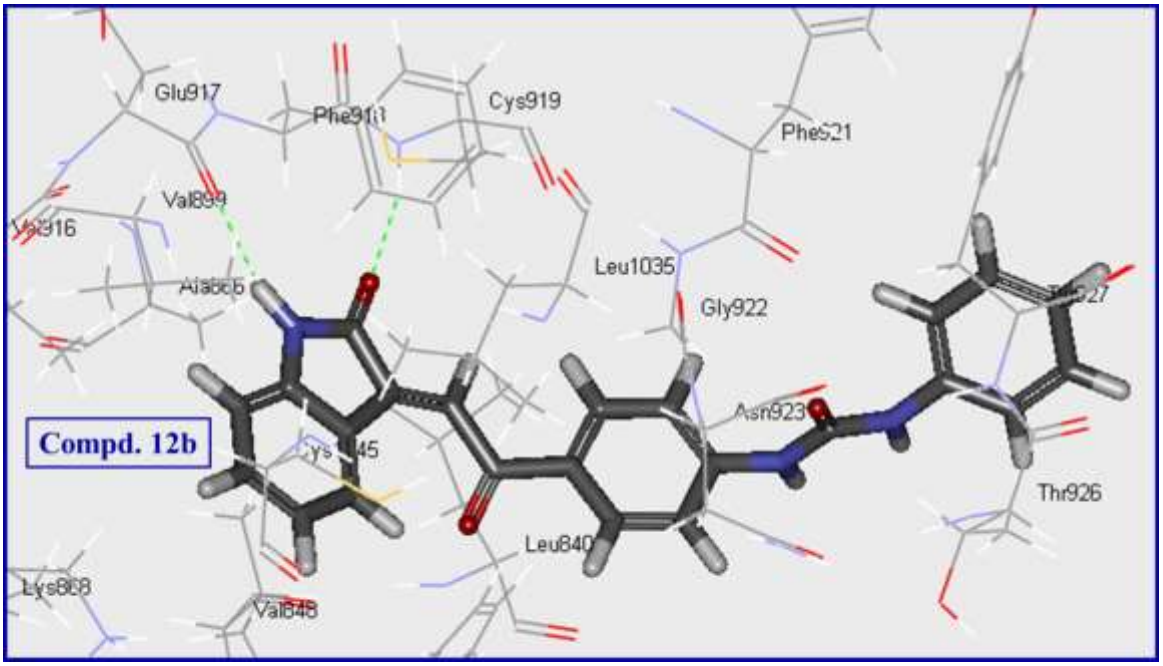
Compd. 12b
Glu917
Cys919
Val899
Leu1035
Gly922
Asn923
Thr926
Leu840
Val848

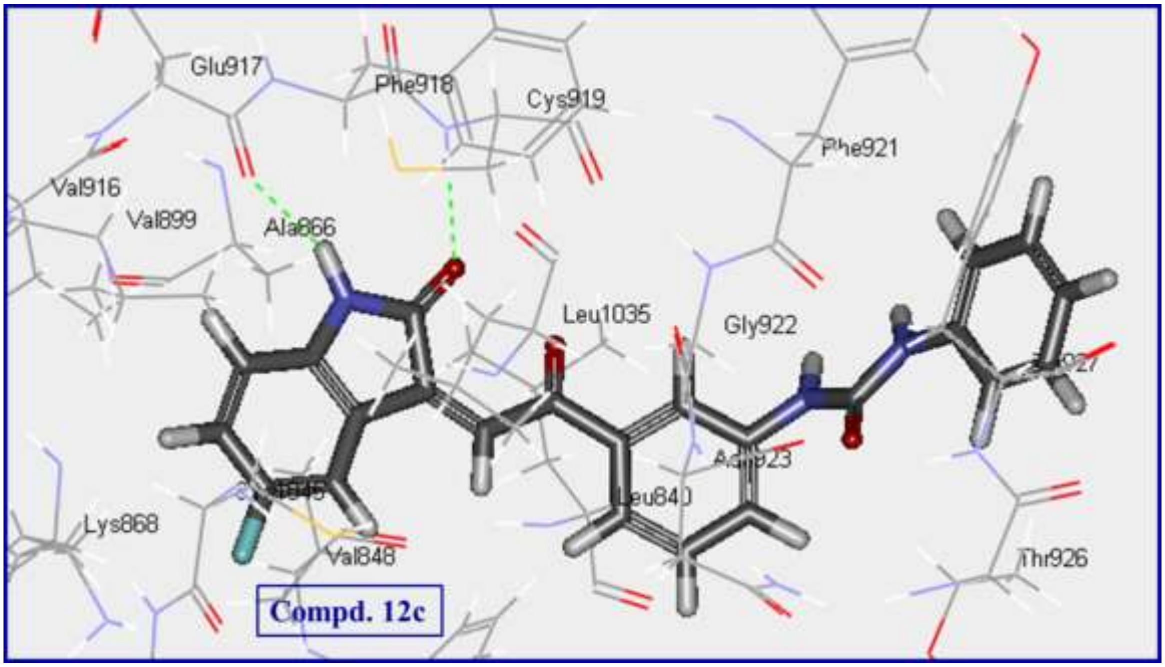
Glu917
Phe918
Cys919
Phe921
Val916
Val899
Ala866
Leu1035
Gly922
Lys868
Val848
Thr926
Compd. 12c

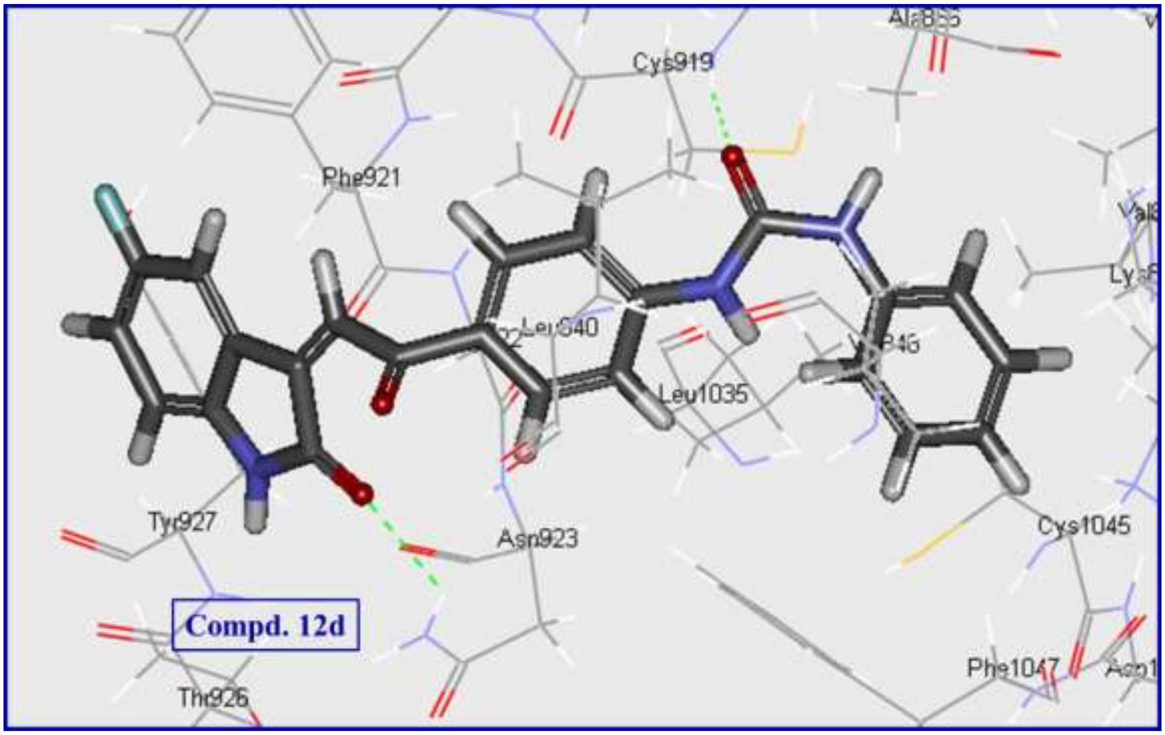
Cys919
Phe921
Leu1035
Tyr927
Asn923
Cys1045
Thr926
Phe1047
Compd. 12d

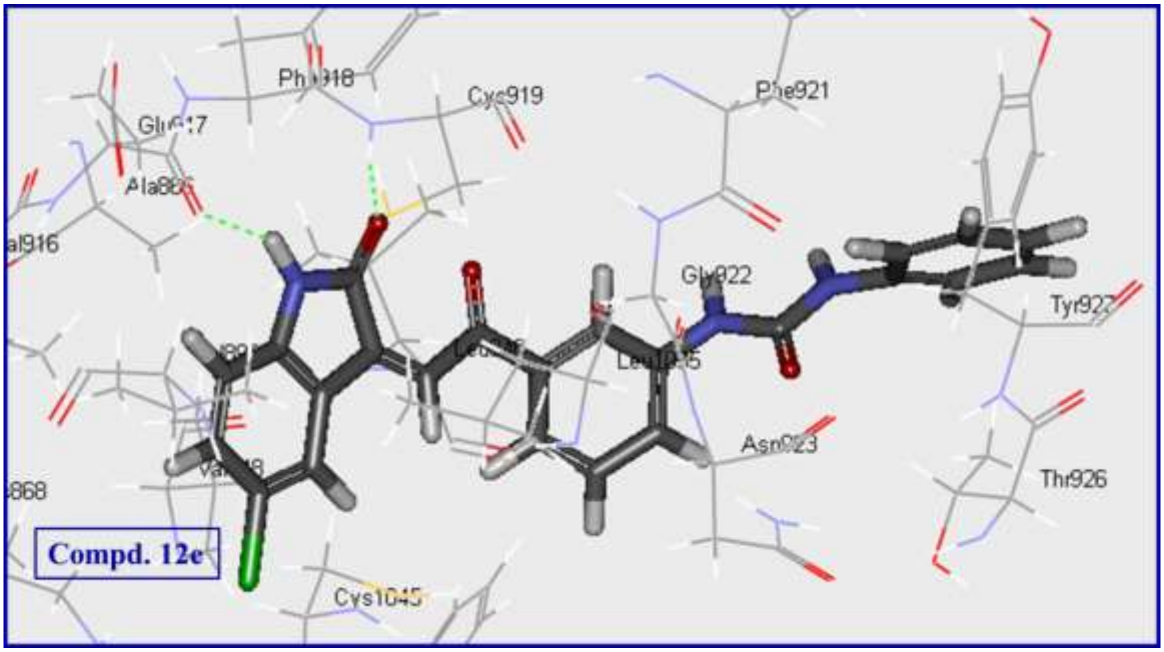
Phe921
Cys919
Tyr922
Thr926
Compd. 12e
Cys1045

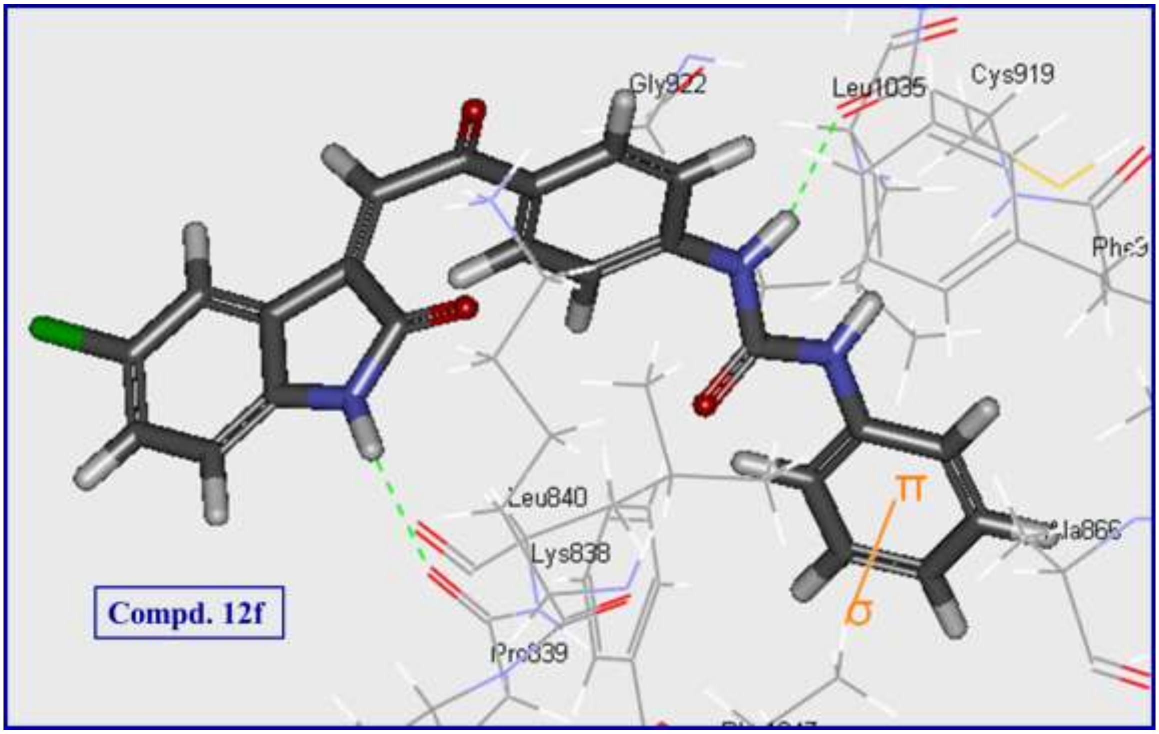
Gly922
Leu1035
Cys919
Leu840
Lys838
Compd. 12f
Pro839
π
σ

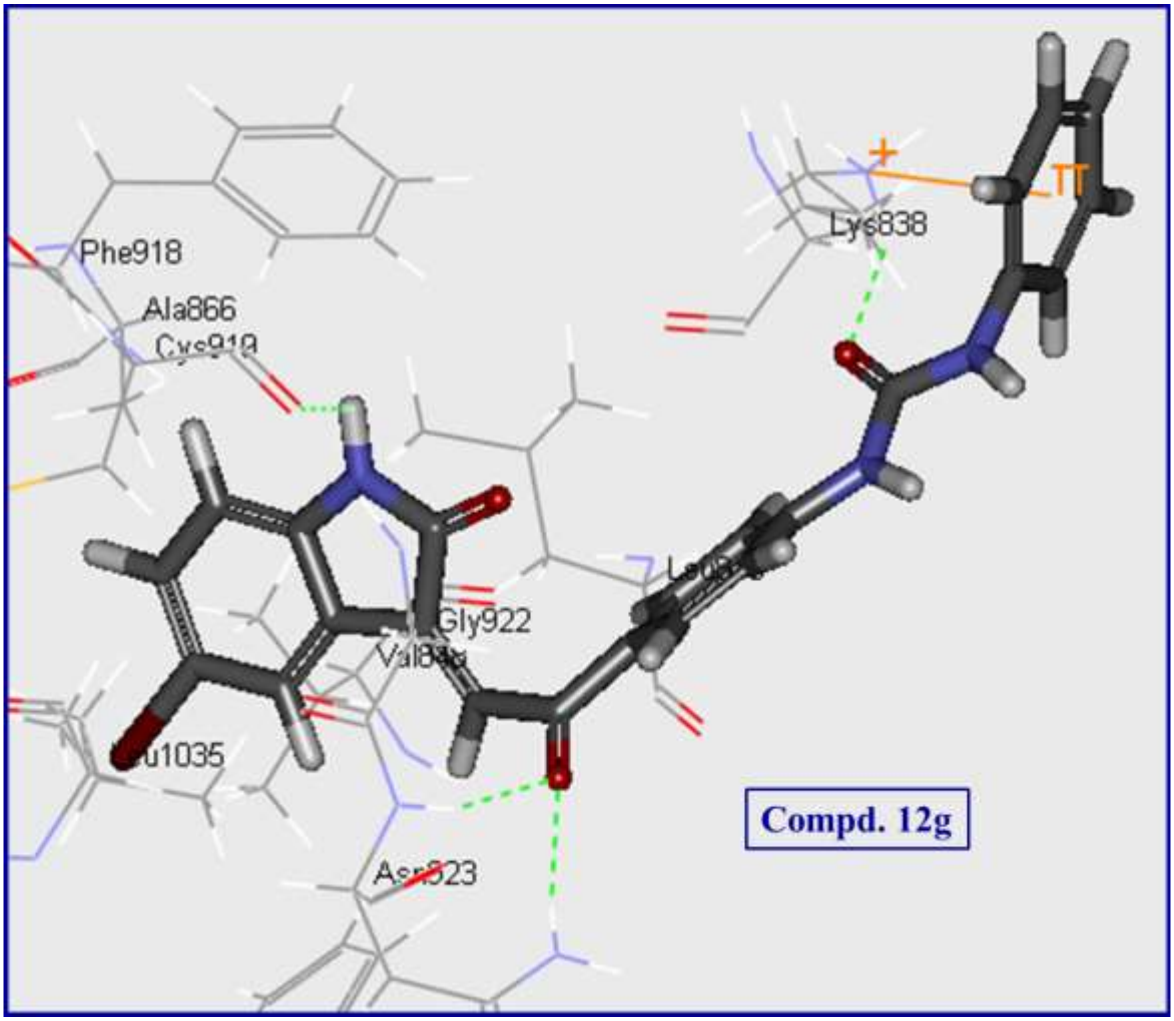
Phe918
Ala866
Lys838
Gly922
Compd. 12g

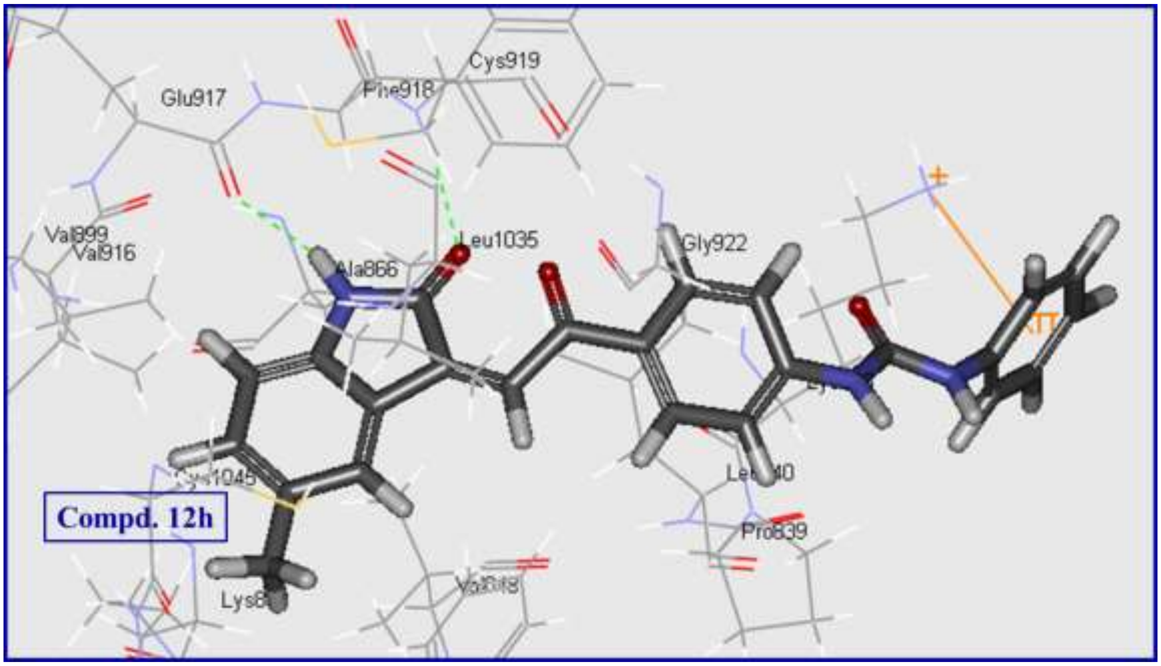
Glu917
Phe918
Cys919
Val899
Val916
Leu1035
Ala866
Gly922
Pro839
Compd. 12h

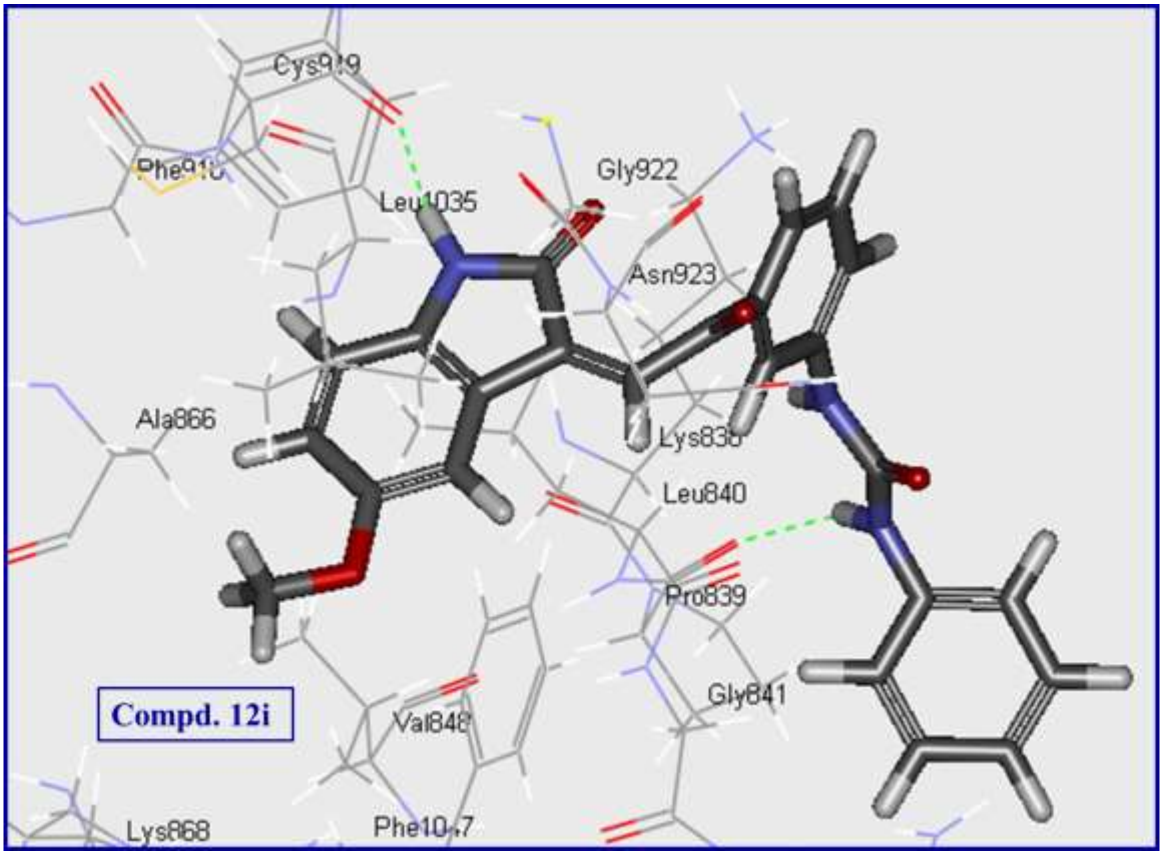

Compd. 12i

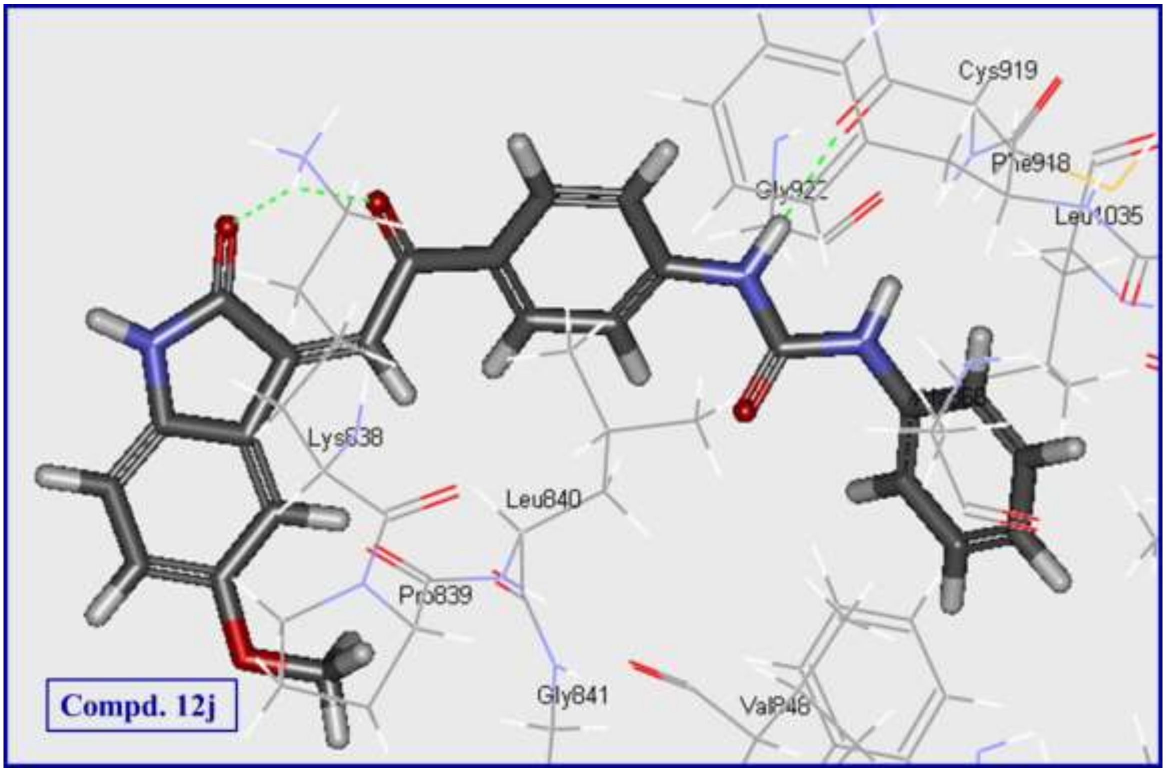

Compd. 12j

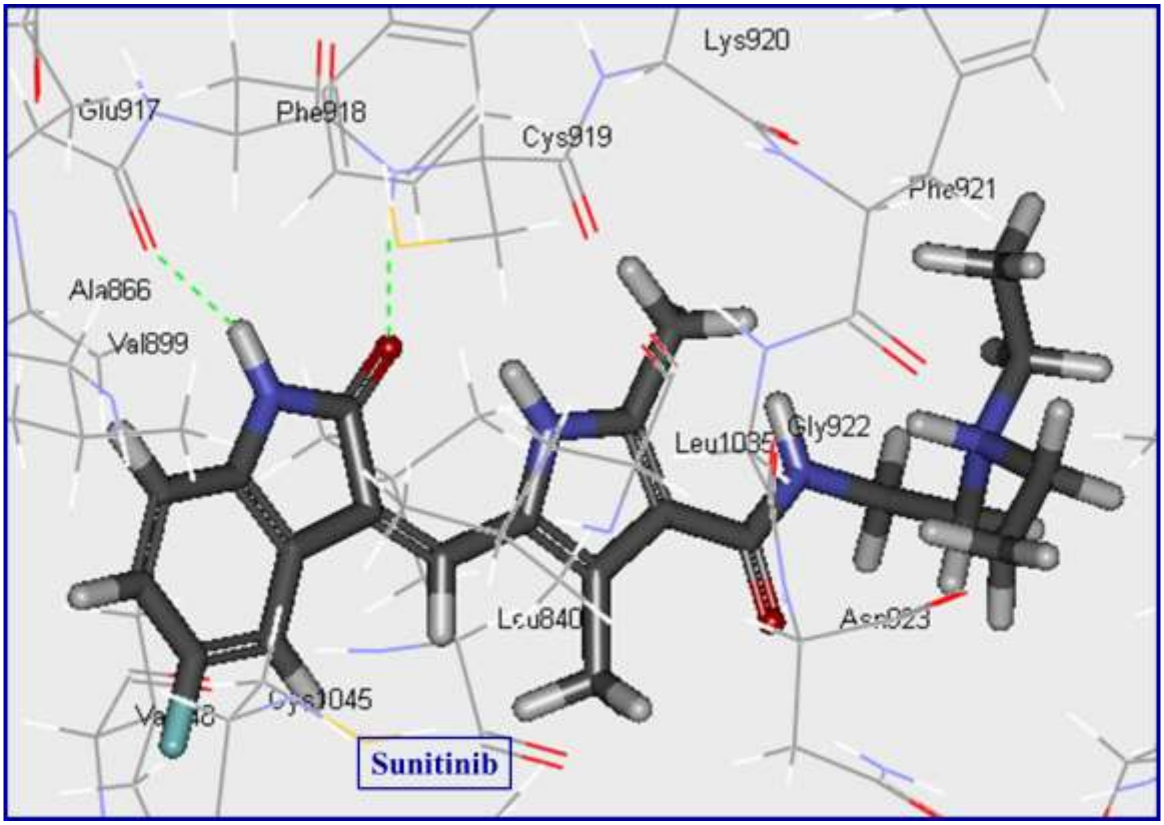

Fig. S67. Docking poses of the **12a–j** and sunitinib (co-crystallized ligand) in PDB ID: 3AGD.

.
